# Supplementary material for: Metabolic reprogramming in the spinal cord drives the transition to pain chronicity
Source: bioRxiv. 2025 Jun 19:2025.01.30.635746. Originally published 2025 Feb 1. Preprint. [Version 2] doi: 10.1101/2025.01.30.635746 (PMC11838349; doi:10.1101/2025.01.30.635746)
Supplement: 1 — Supplemental Fig. S1. (Related to Figure 1) Effects of formalin injection on gene transcription and metabolite levels in ipsilateral L4-L6 spinal cord of SD-fed mice. (A, B) Transcription of genes involved in (A) glycolysis and (B) Krebs’s cycle and oxidative phosphorylation. Data are expressed as log2 changes (formalin vs vehicle) (n = 6; multiple unpaired t test). (C, D) Concentrations of (C) glycolysis and (D) Krebs’s cycle metabolites. Data are expressed as ion counts (mean ± SEM; n = 7–10 per group; Student’s t test). Supplemental Fig. S2. (Related to Figure 1B) Serum concentrations of amino acids and amino-acid metabolites in vehicle-injected mice fed SD (gray boxes; n = 9–10) or MD-1 (blue boxes; n = 6–7) for 25 days. Data are expressed as ion counts (mean ± SEM; Student’s t test). Supplemental Fig. S3. (Related to Figure 1D, E) Concentrations of MD-1 components in ipsilateral L4-L6 spinal cord of vehicle-injected mice fed SD (gray boxes; n = 7–10) or MD-1 (blue boxes; n = 5–7) for 25 days. Data are expressed as ion counts (mean ± SEM; Student’s t test). Supplemental Fig. S4. (Related to Figure 2) Concentrations of (A) glycolysis and (B) Krebs’ cycle metabolites in ipsilateral L4-L6 spinal cord of vehicle-injected mice fed SD (gray boxes) or MD-1 (blue boxes). Data are expressed as ion counts (mean ± SEM; n = 7–10 per group; Student’s t test). Supplemental Fig. S5. (Related to Figure 2D) Effects of formalin injection on Complex IV transcription in ipsilateral L4-L6 spinal cord of vehicle-injected mice fed SD or MD-1. Red: increase; blue: decrease. Data are expressed as log2 changes (formalin vs vehicle; n = 6 per group). Supplemental Fig. S6. (Related to Figure 3) Formalin injection activates AKT/mTORC1 in ipsilateral L4-L6 spinal cord and MD-1 counters this activation. (A) Representative Western blot images showing levels of phospho-AKT (p-AKT), AKT, phospho-mTOR (p-mTOR), and mTOR in vehicle- or formalin-injected mice fed SD or MD-1. β-actin is the loading [file NIHPP2025.01.30.635746v2-supplement-1.pdf]

Supplemental Fig. S1

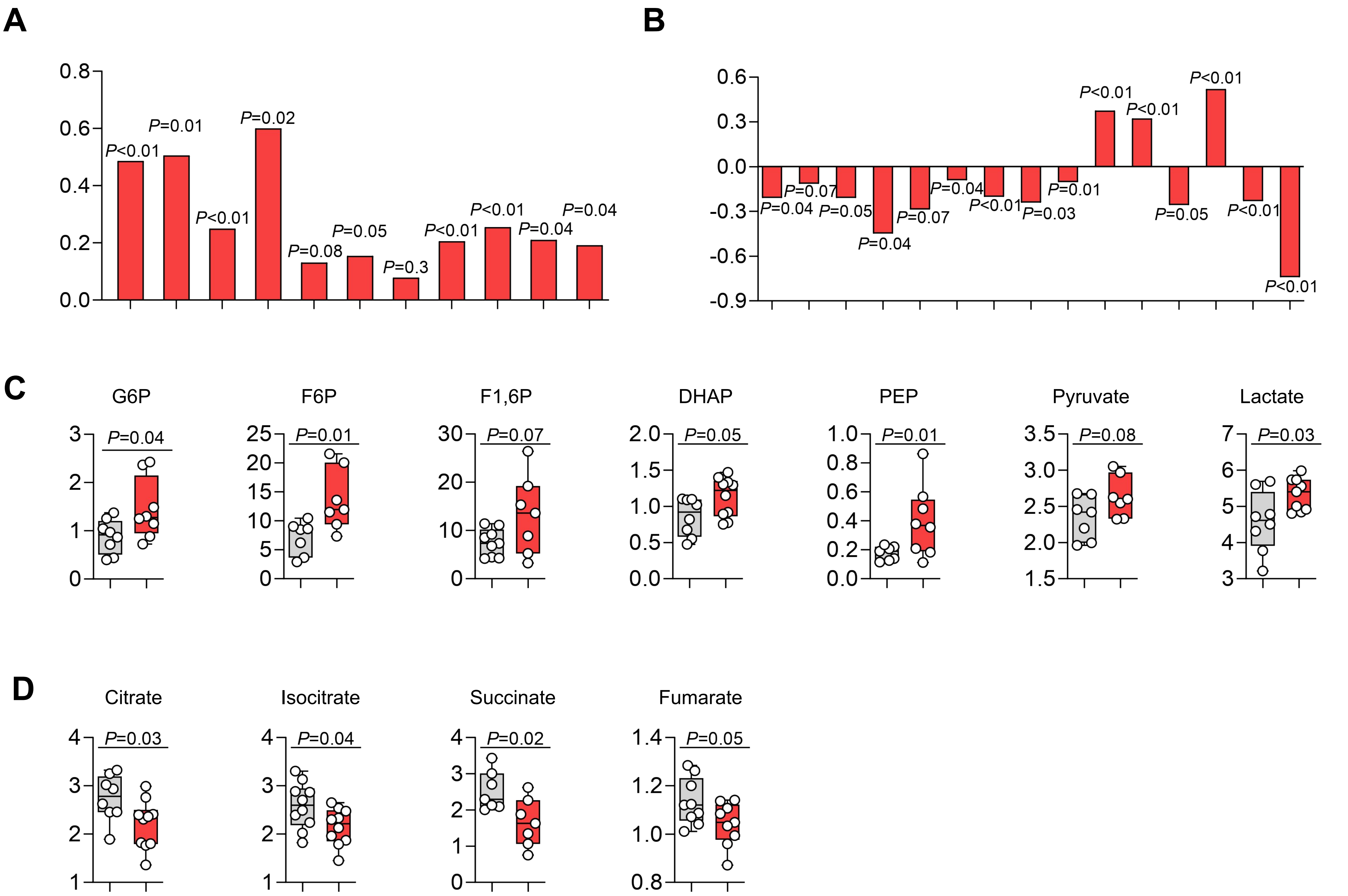

Supplemental Fig. S2

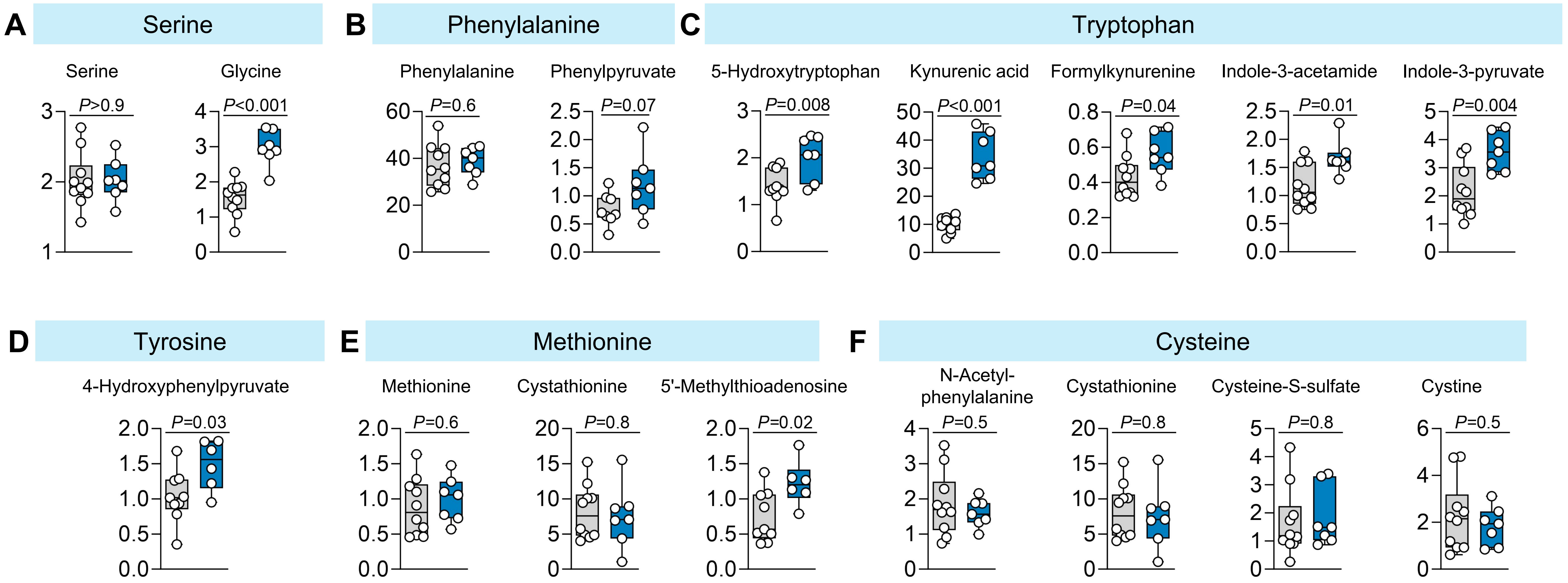

Supplemental Fig. S3

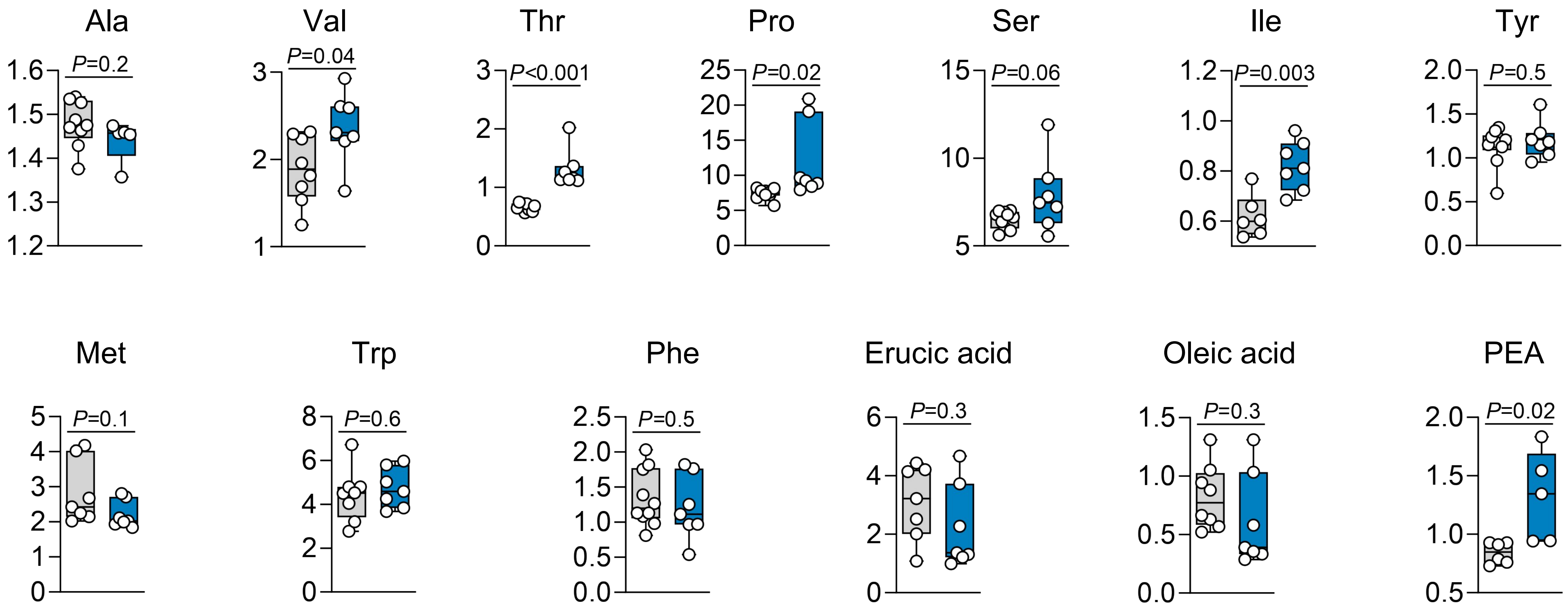

Supplemental Fig. S4

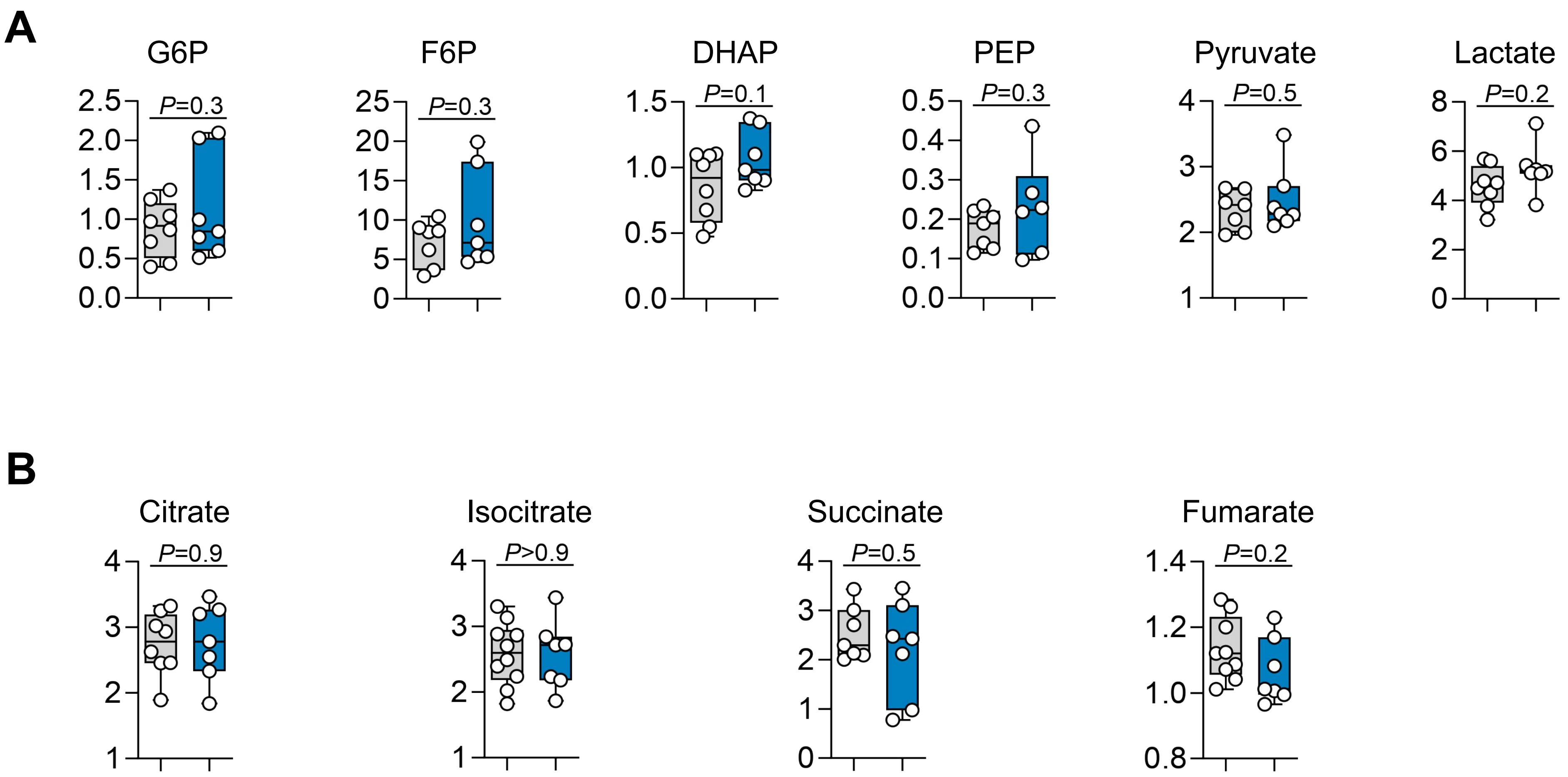

Supplemental Fig. S5

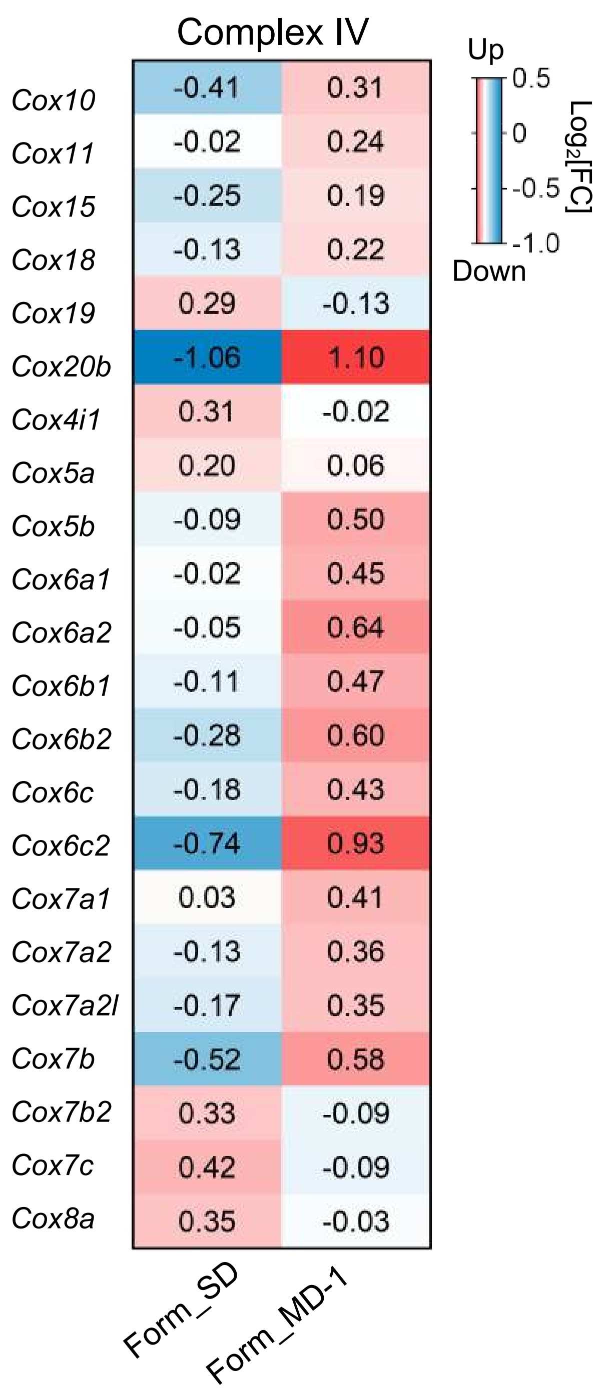

Supplemental Fig. S6

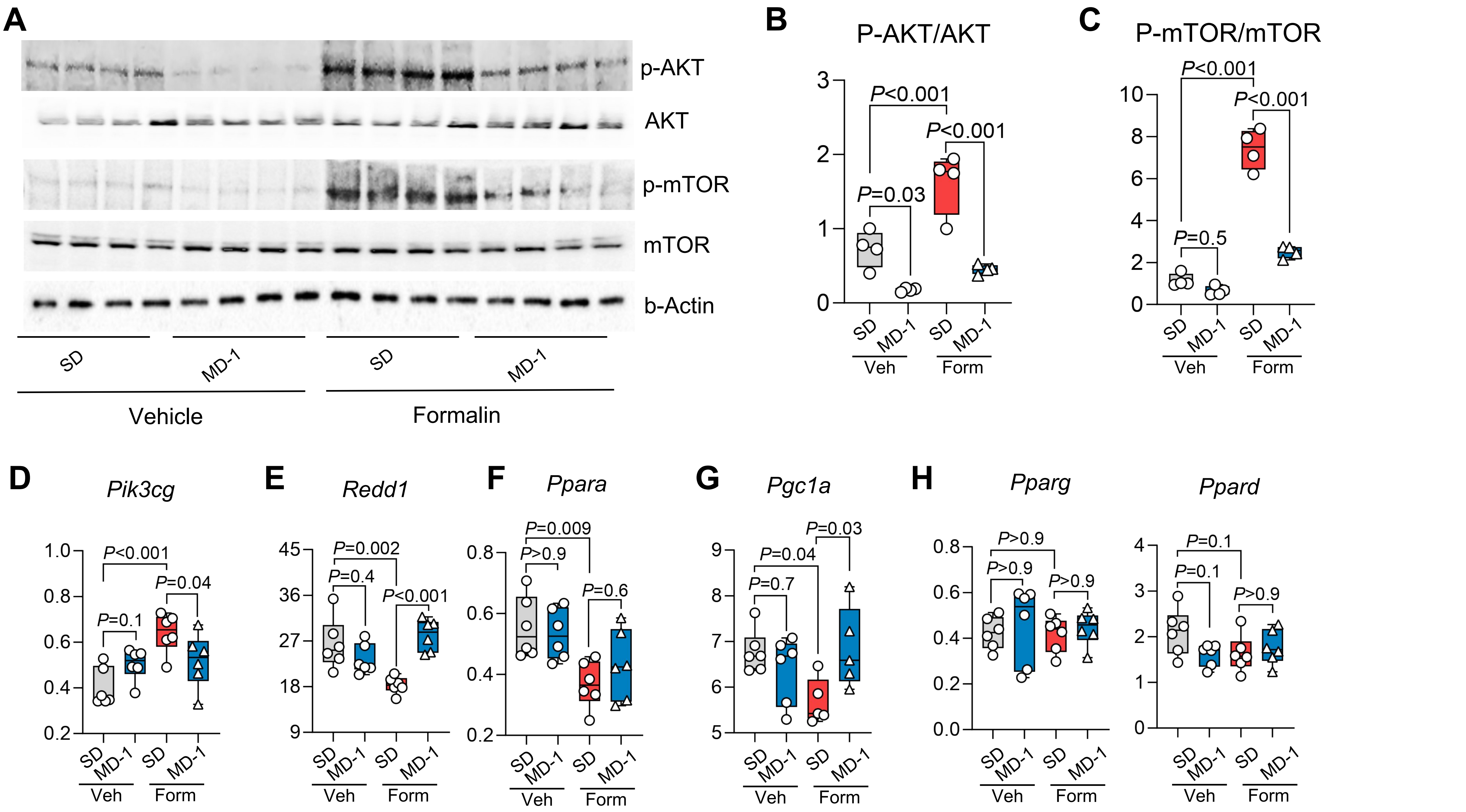

Supplemental Fig. S7

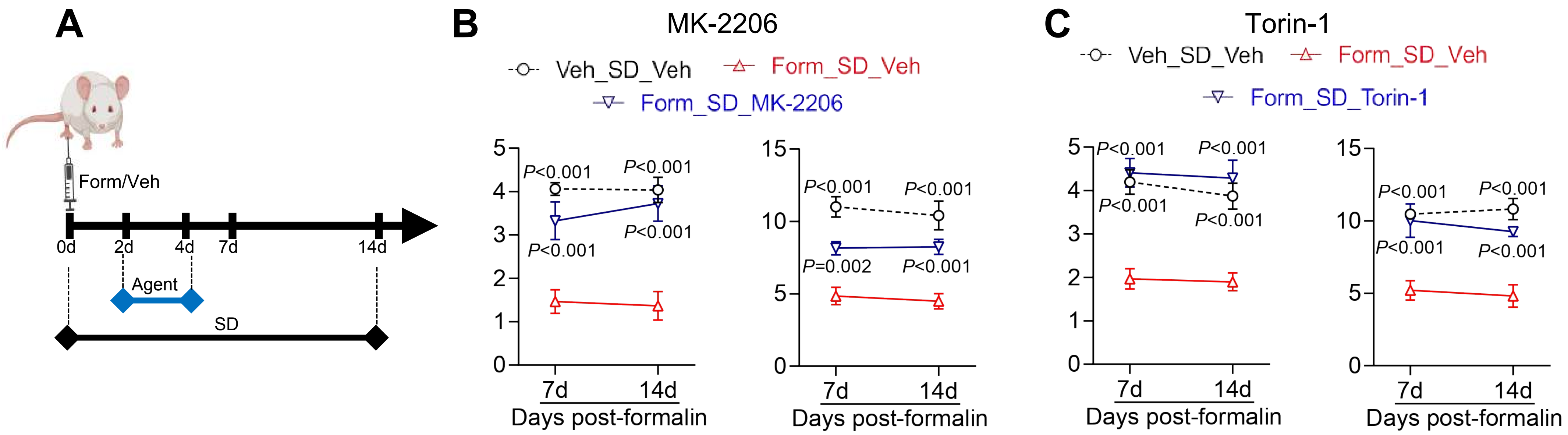

Supplemental Fig. S8

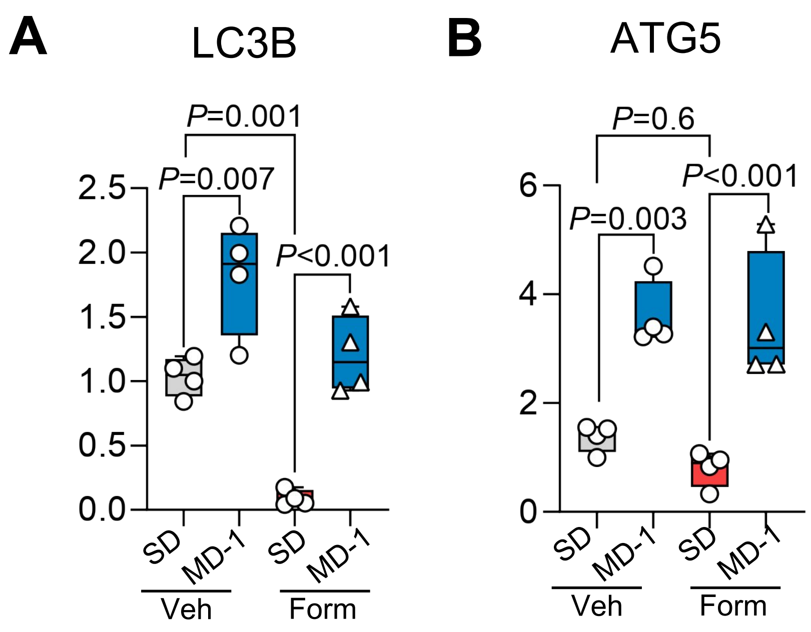

Supplemental Fig. S9

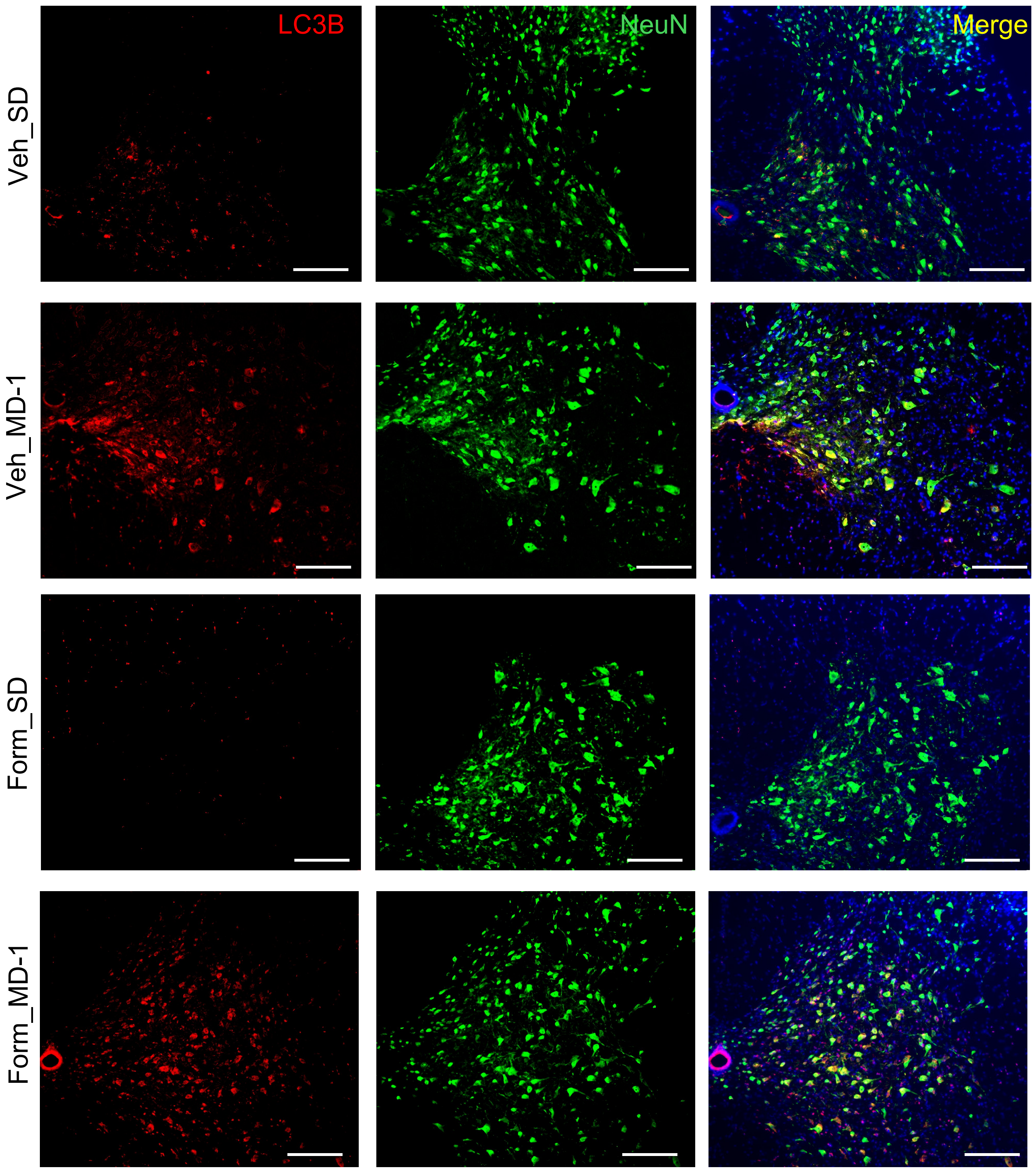

Supplemental Fig. S10

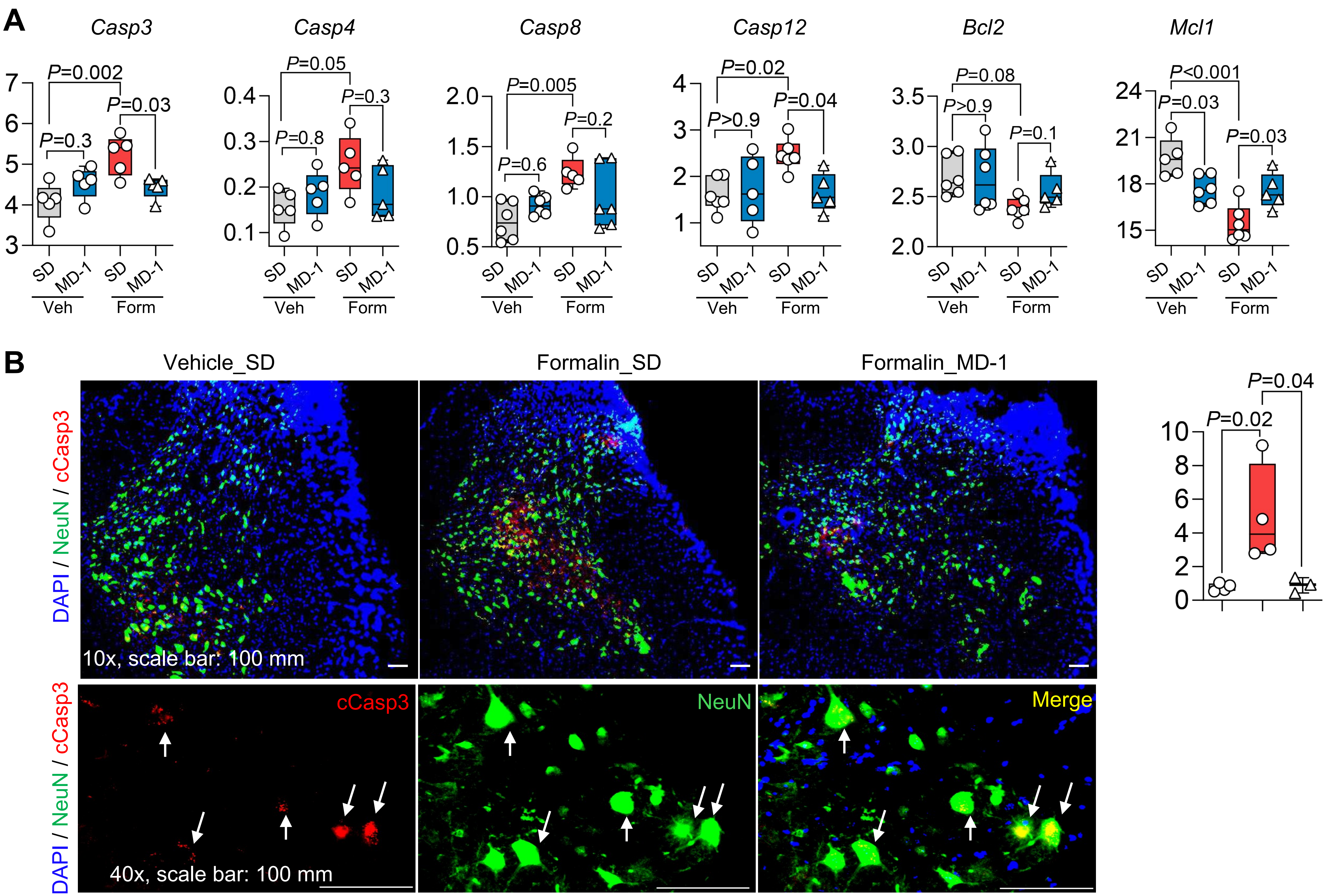

Supplemental Fig. S11

A

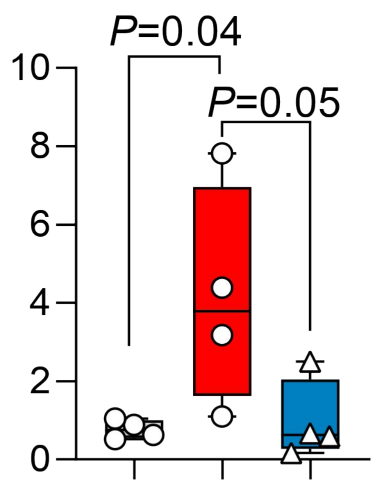

B

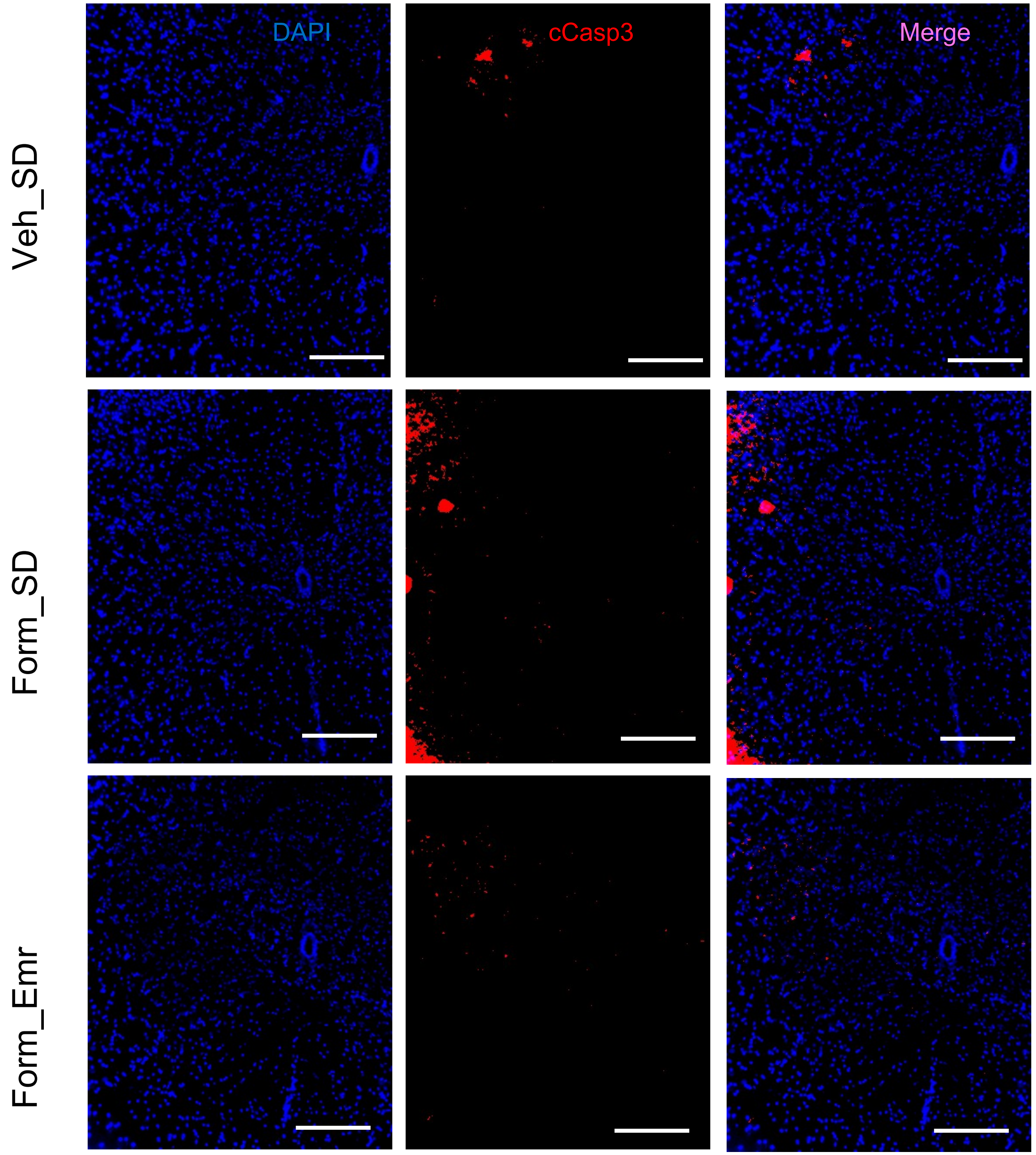

Supplemental Fig. S12

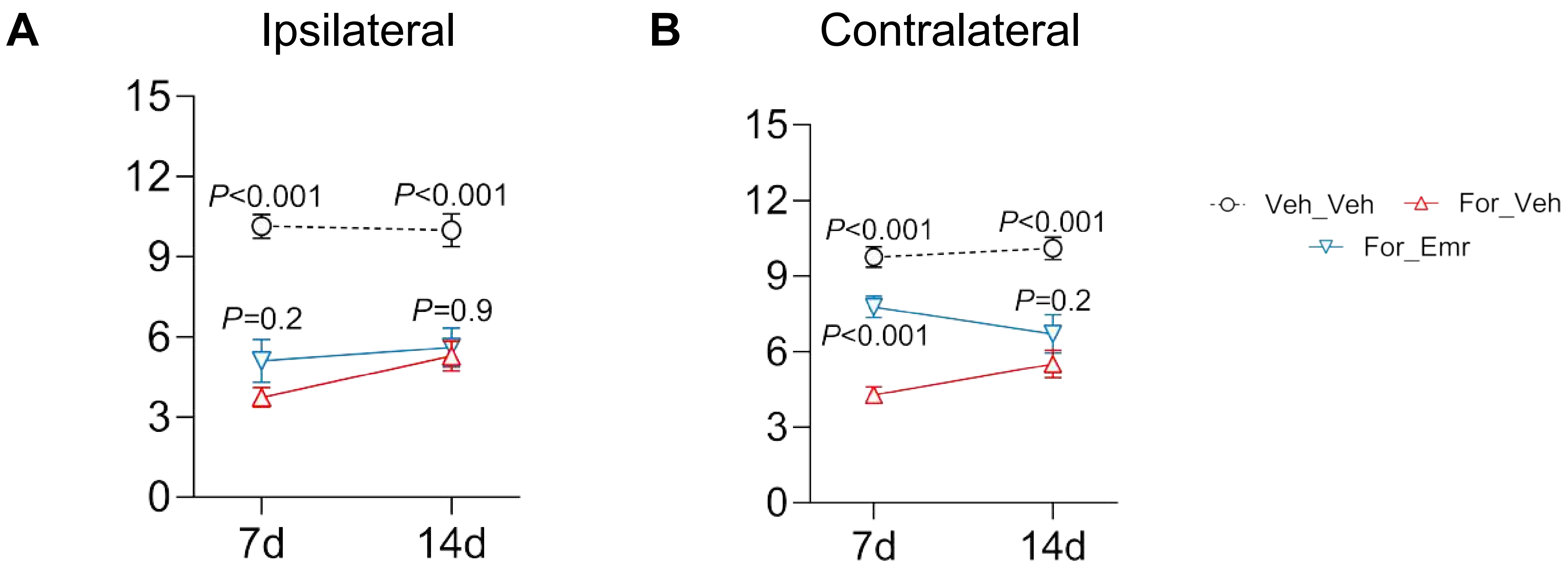

Supplemental Fig. S13

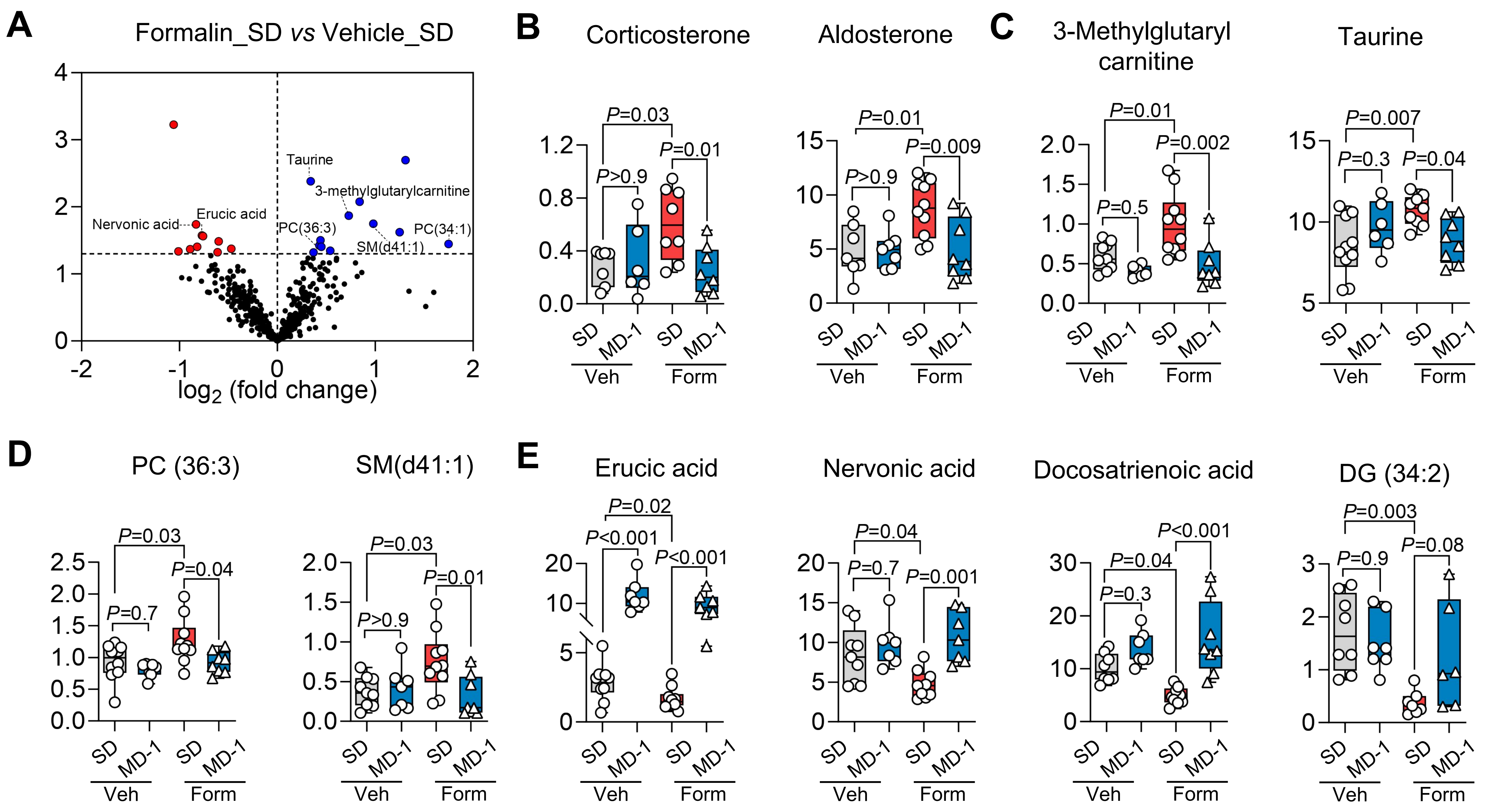

Supplemental Fig. S14

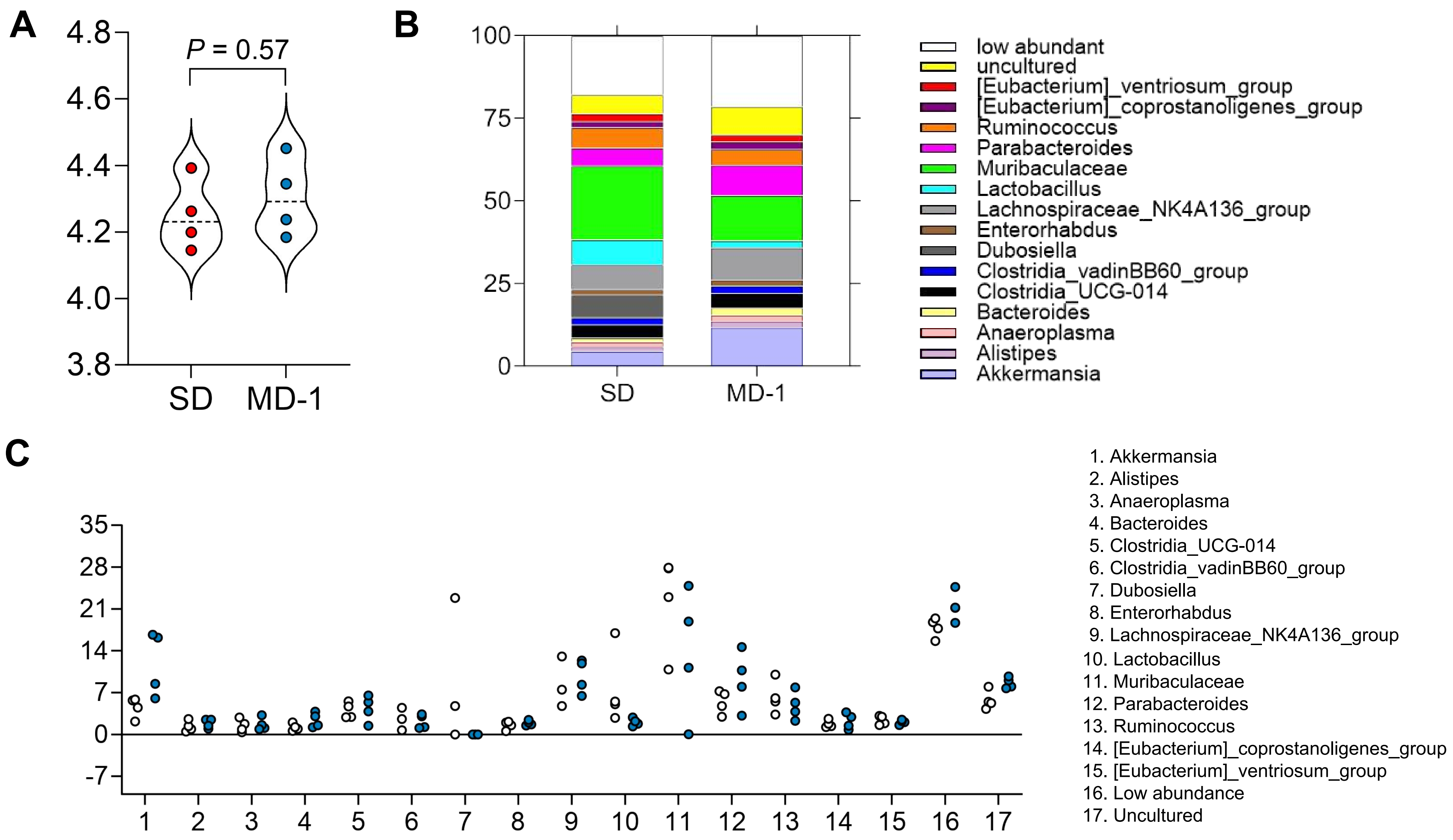

Supplemental Fig. S15

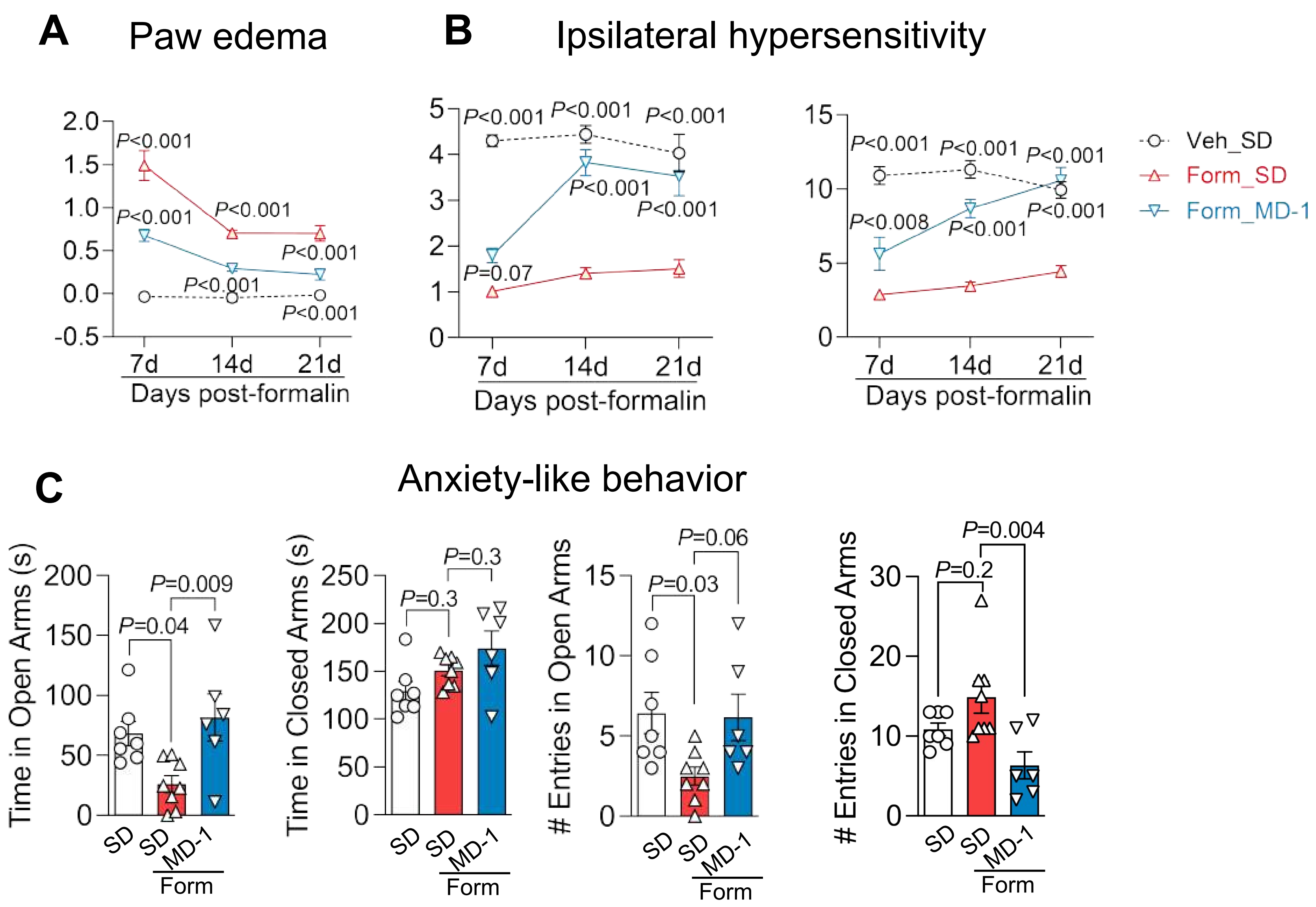

Supplemental Fig. S16

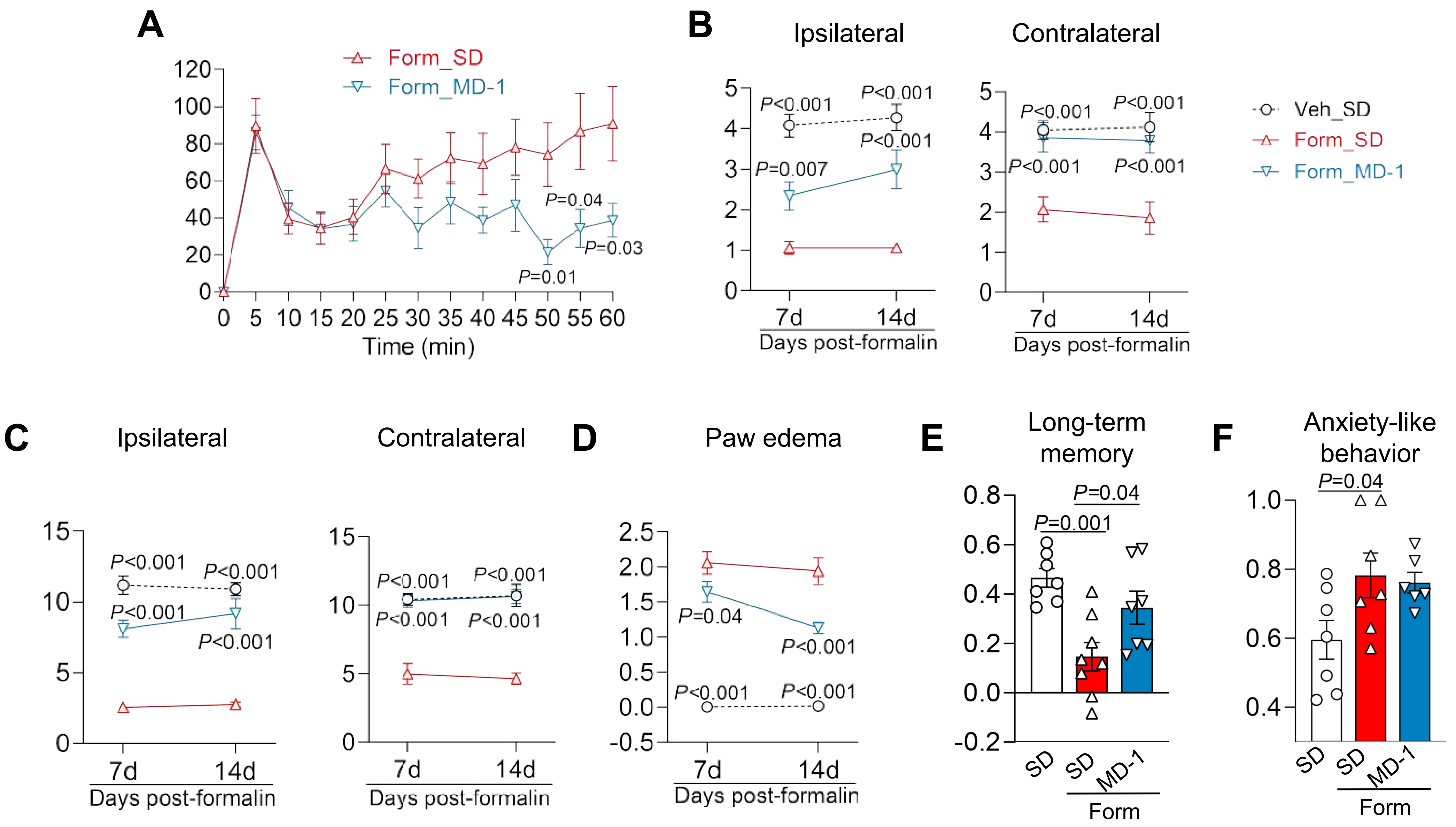

Supplemental Fig. S17

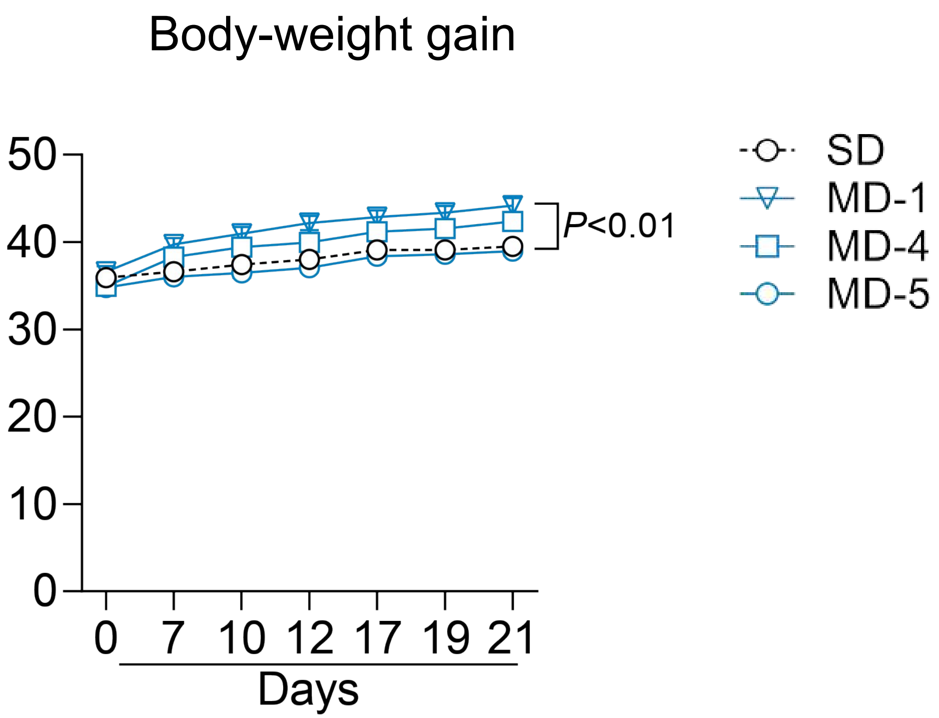

Supplemental Fig. S18

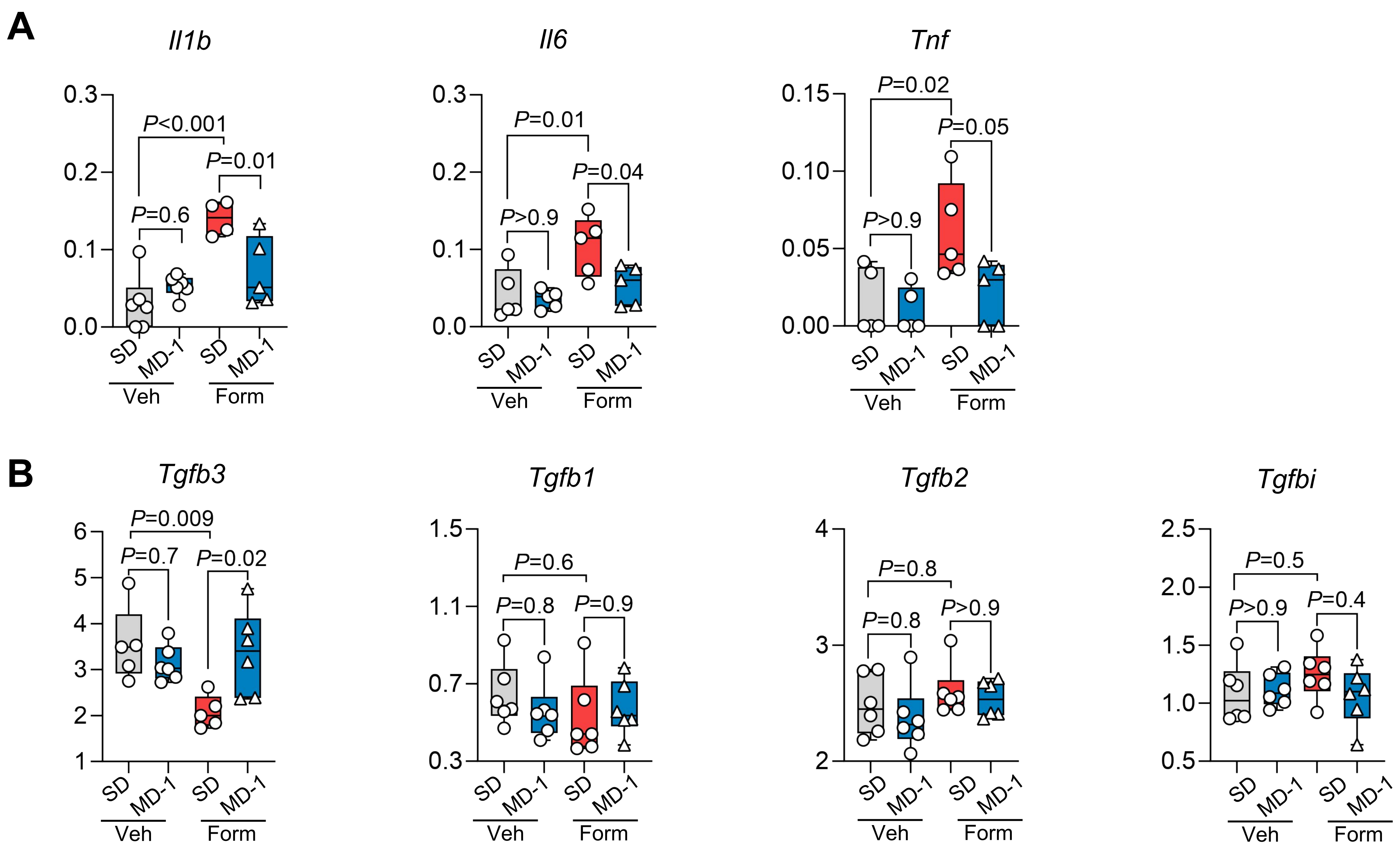

Supplemental Fig. S19

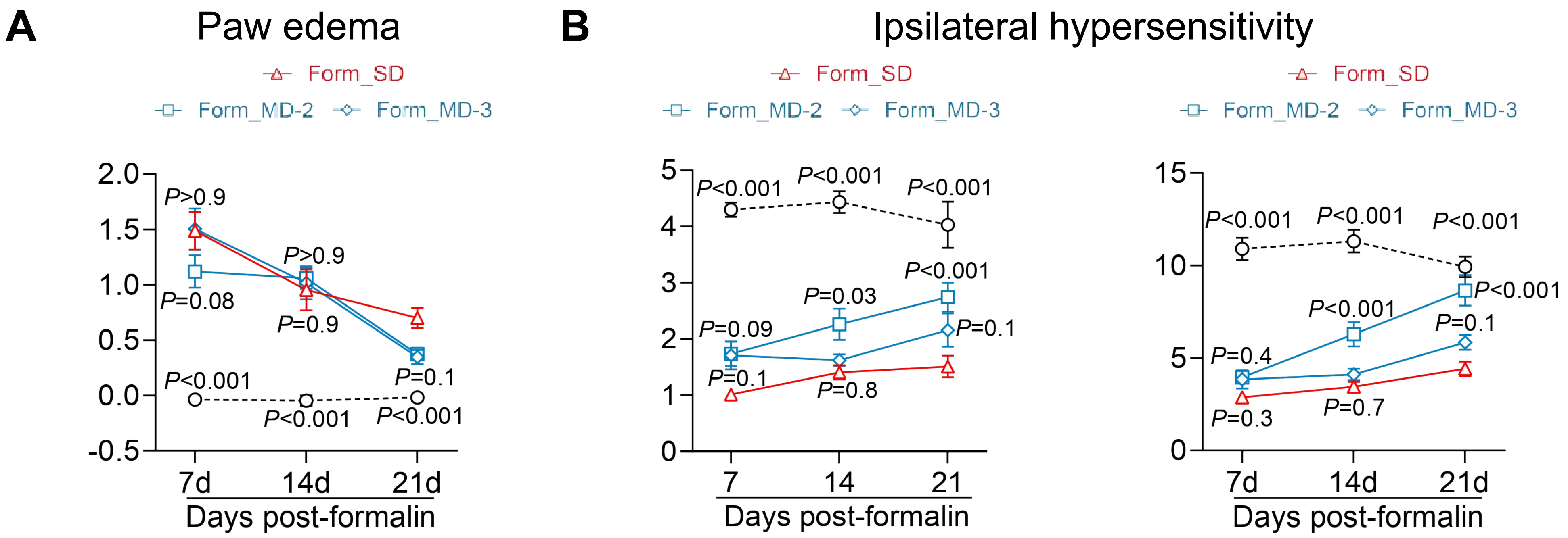

Supplemental Fig. S20

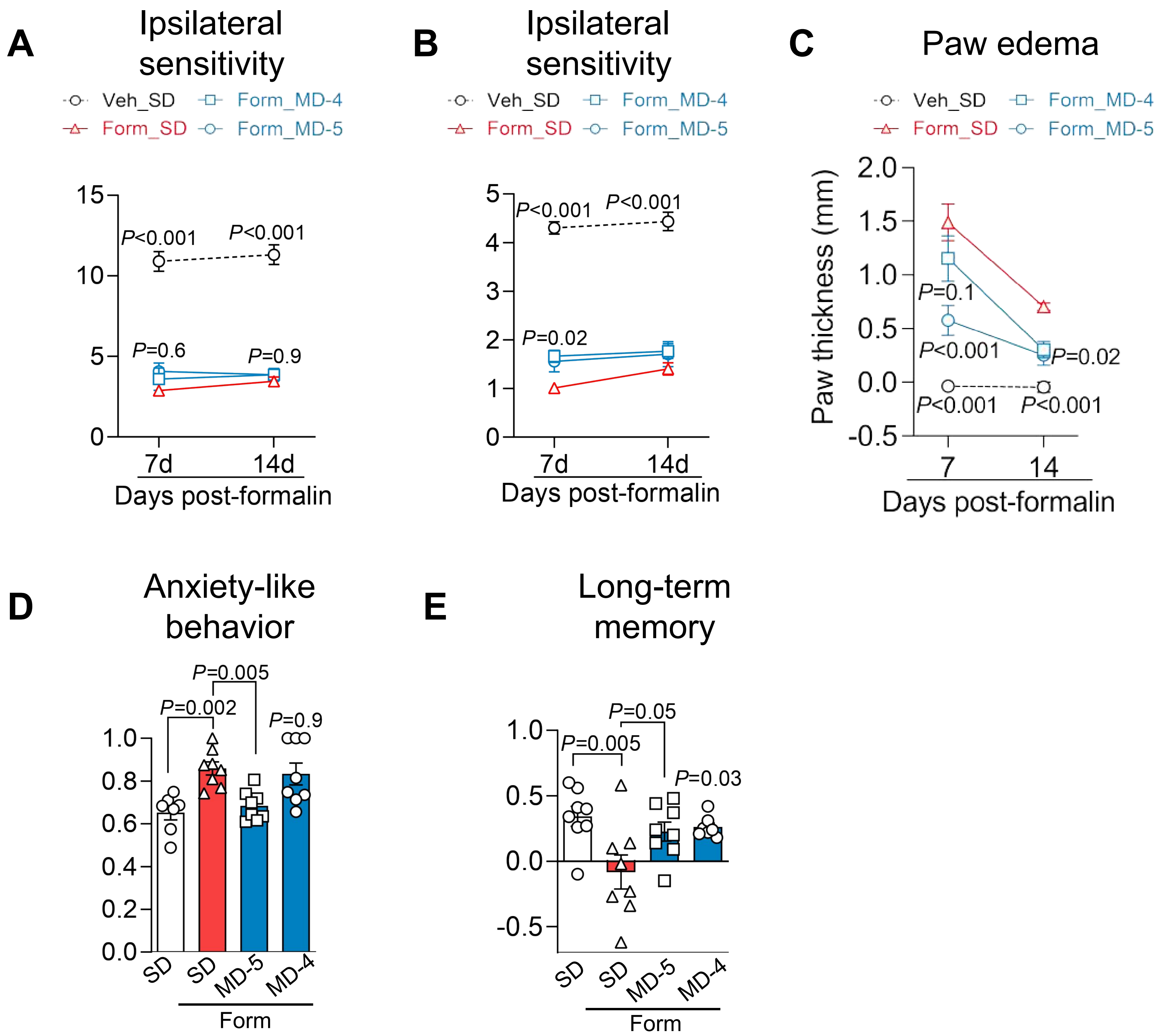

Supplemental Fig. S21

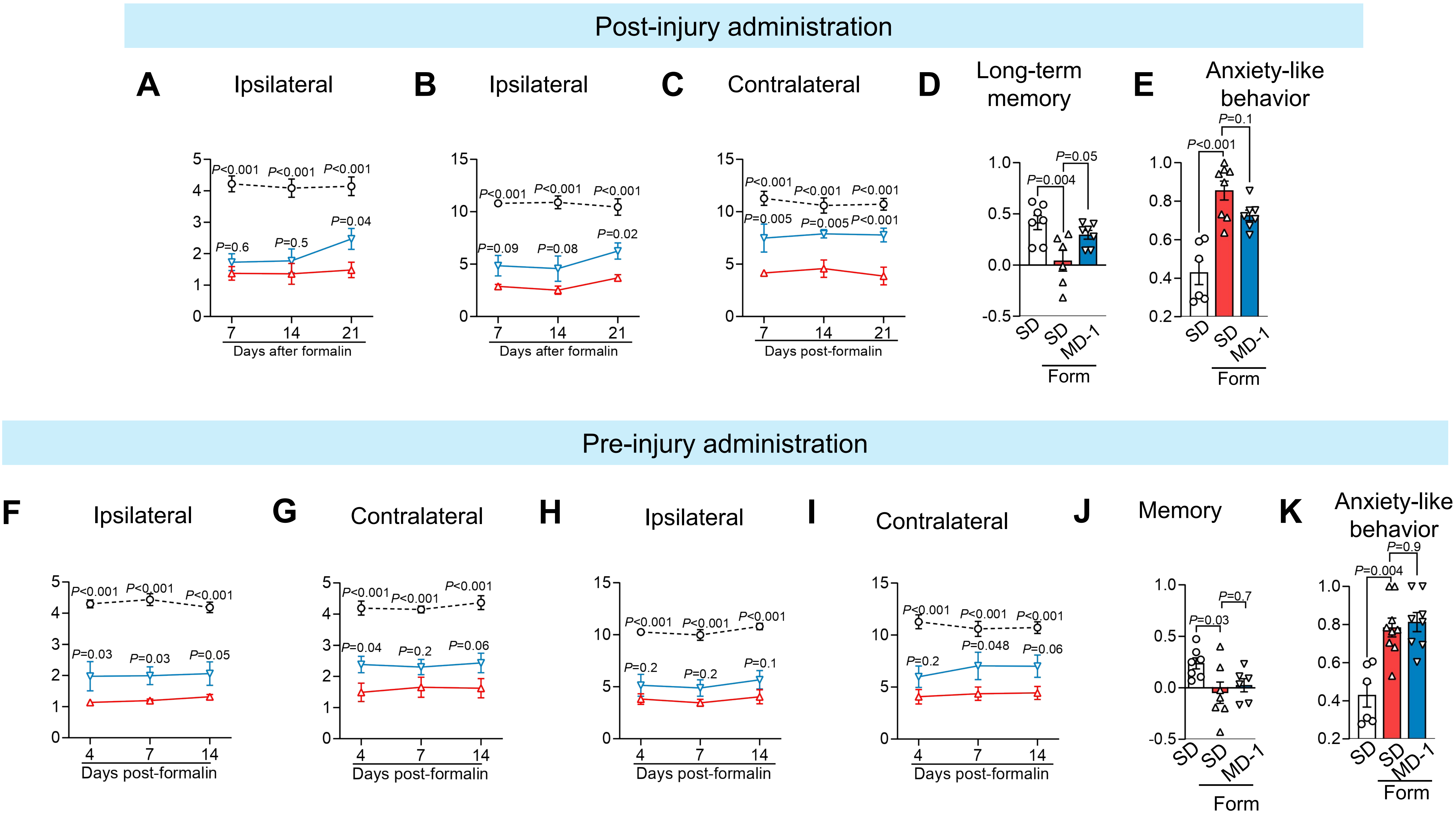

Supplemental Fig. S22

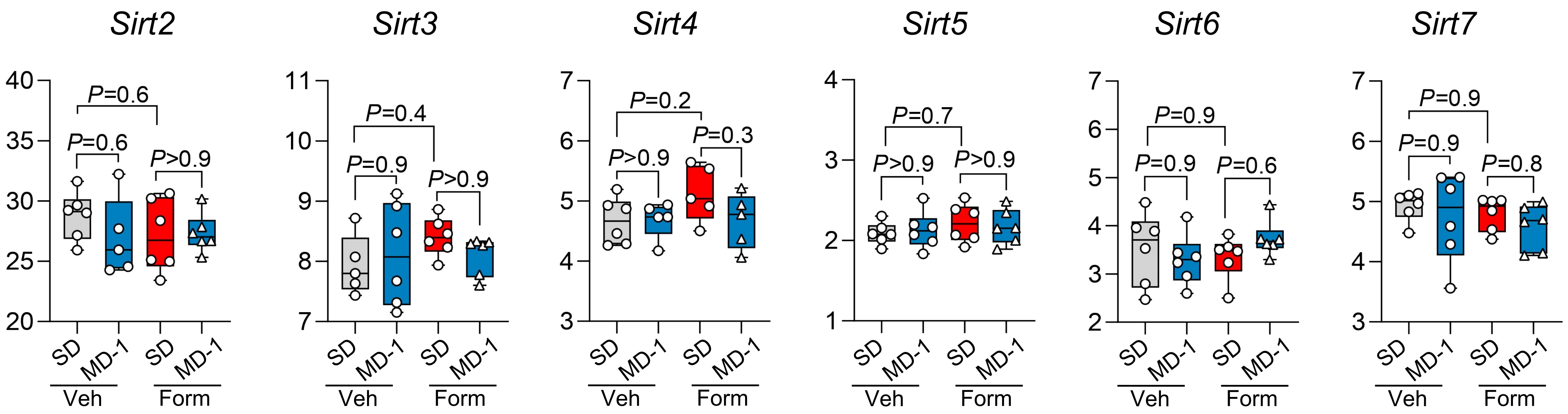

Supplemental Fig. S23

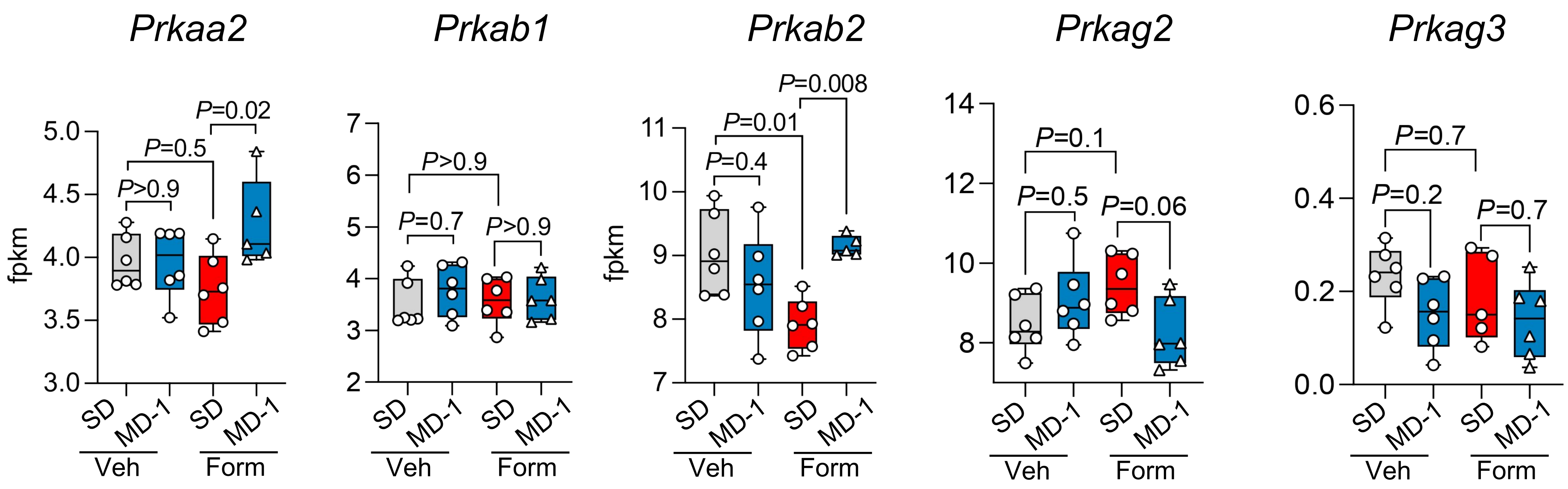

Supplemental Fig. S24

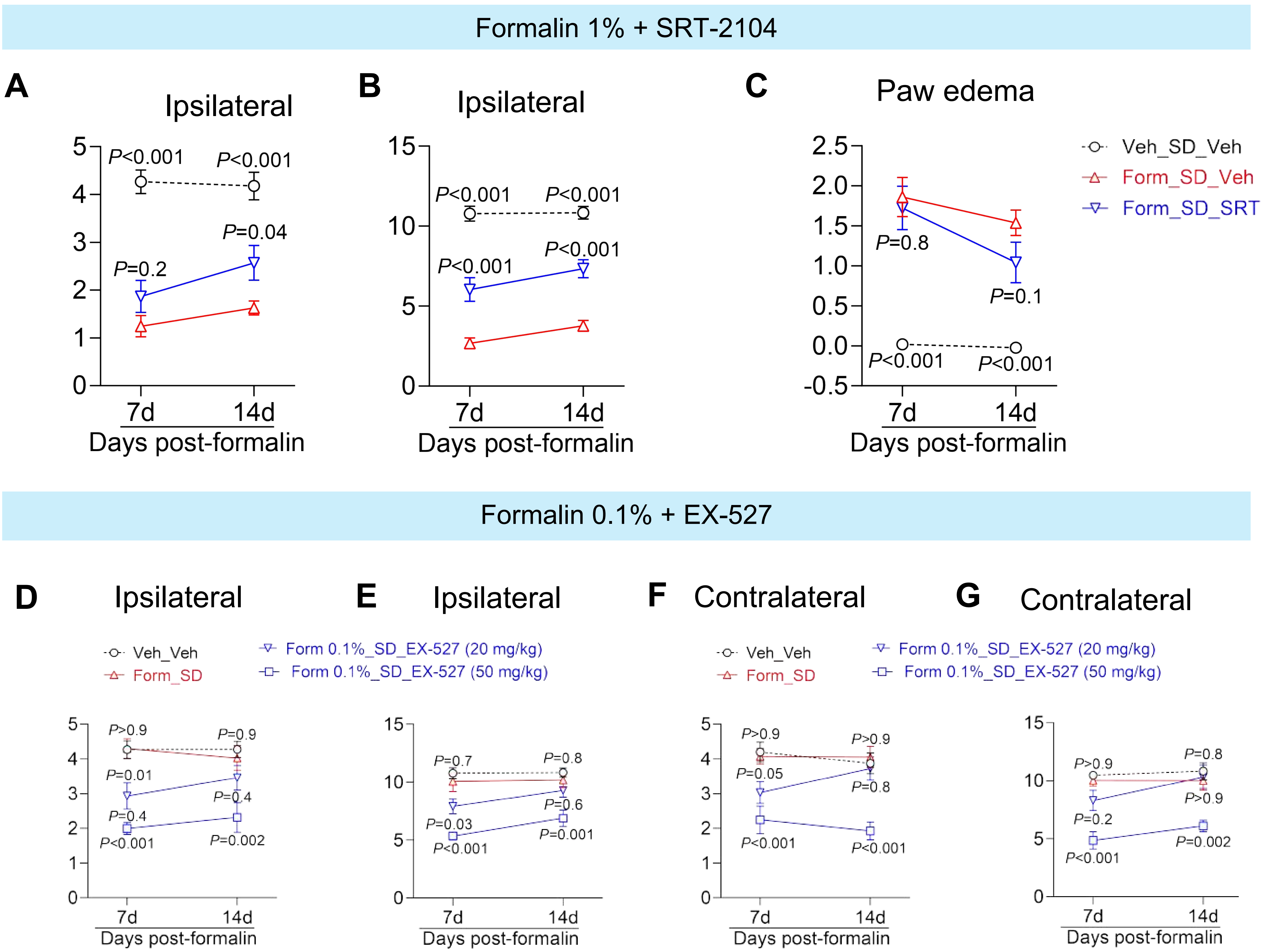

Supplemental Fig. S25

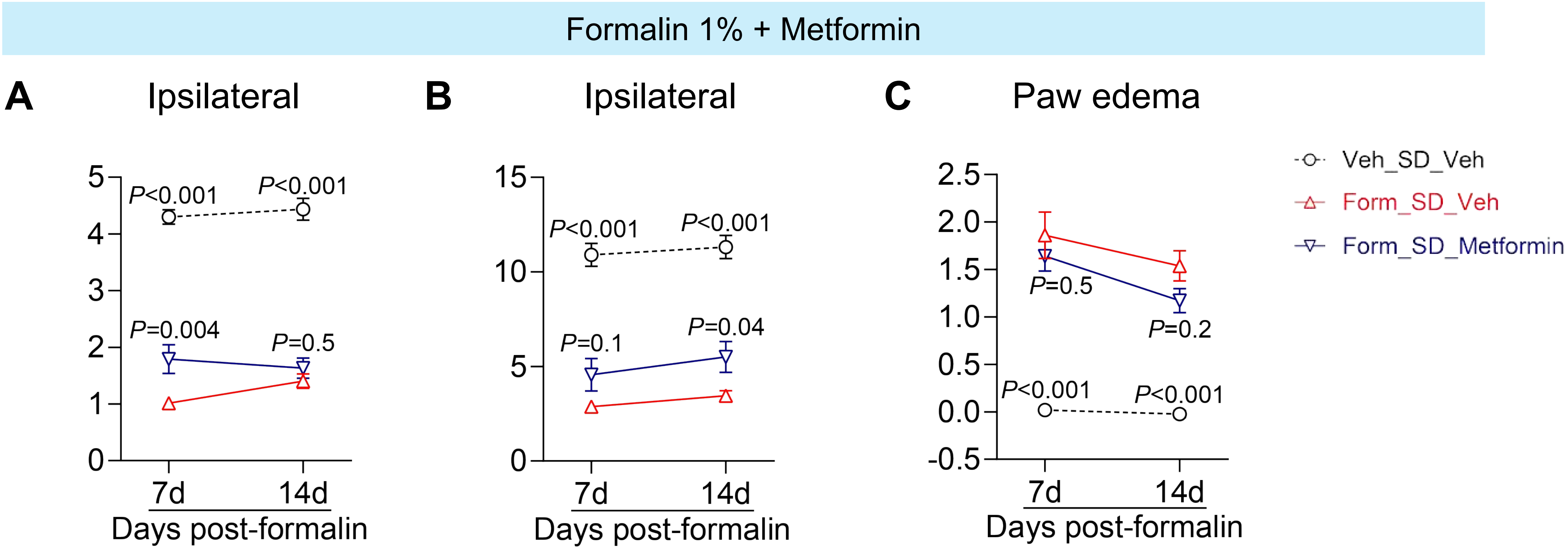

Supplemental Fig. S26

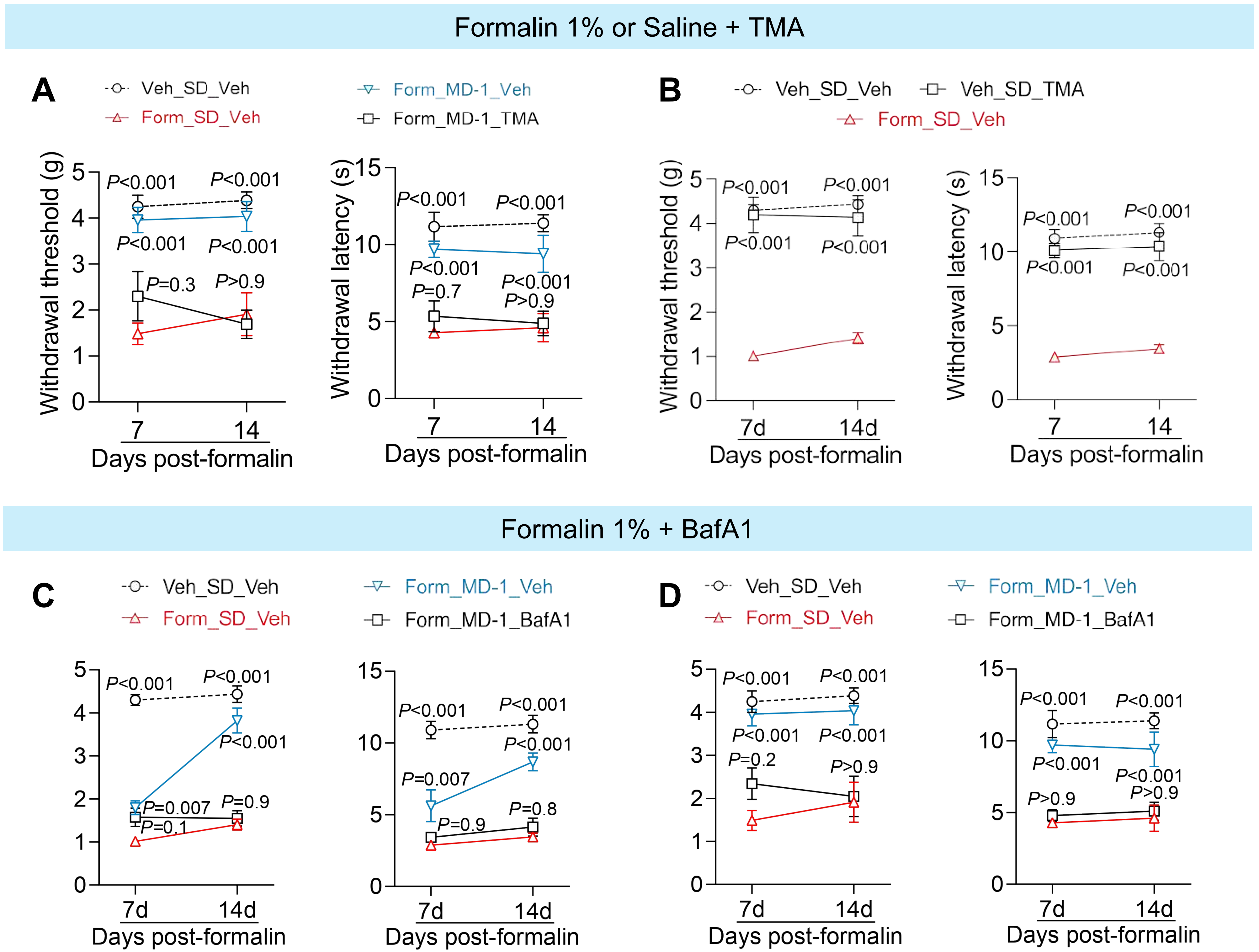

Supplemental Fig. S27

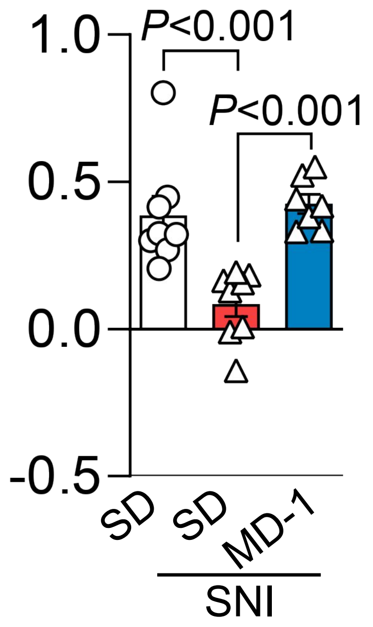

Supplemental Fig. S28

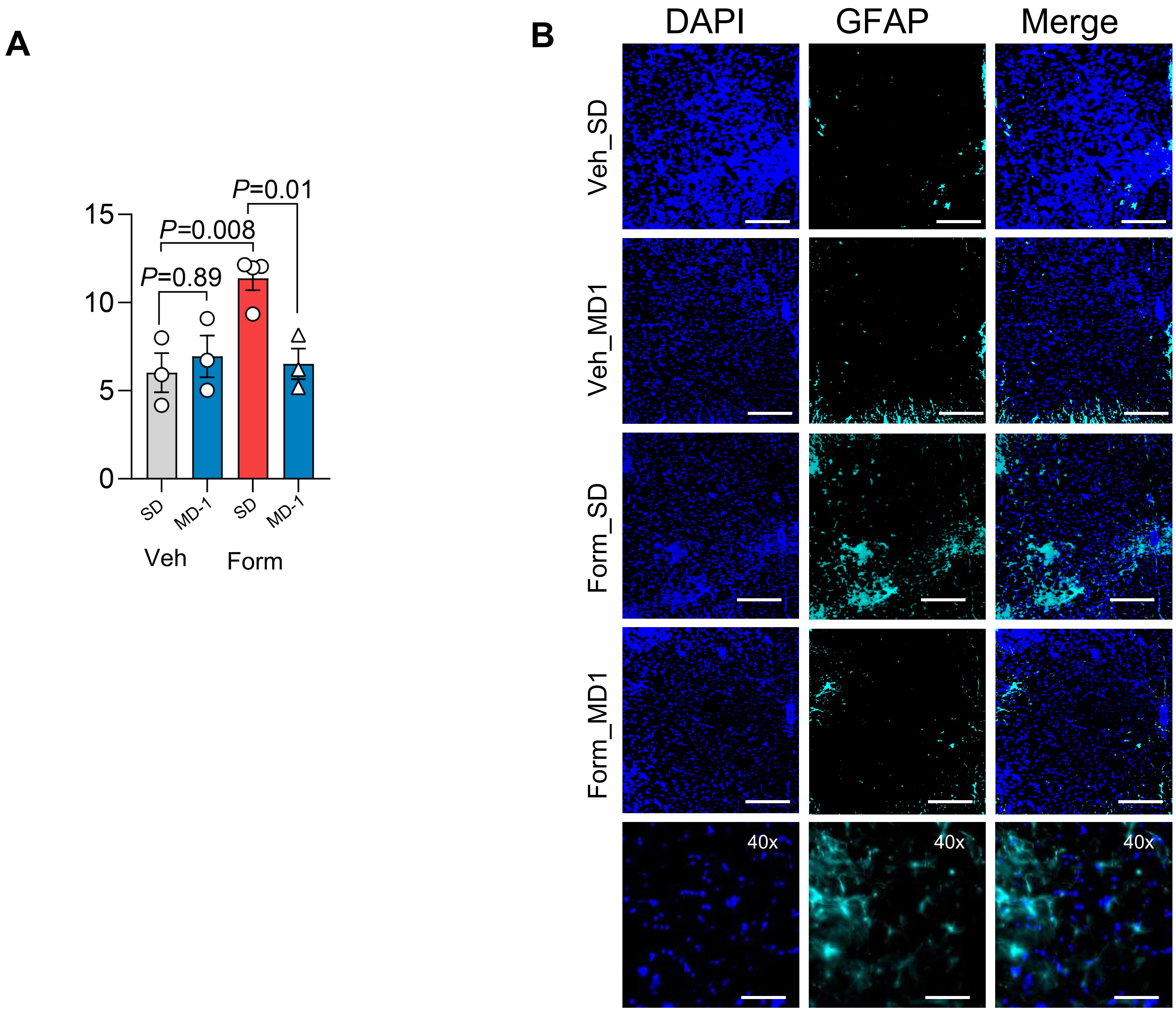

Supplemental Fig. S29

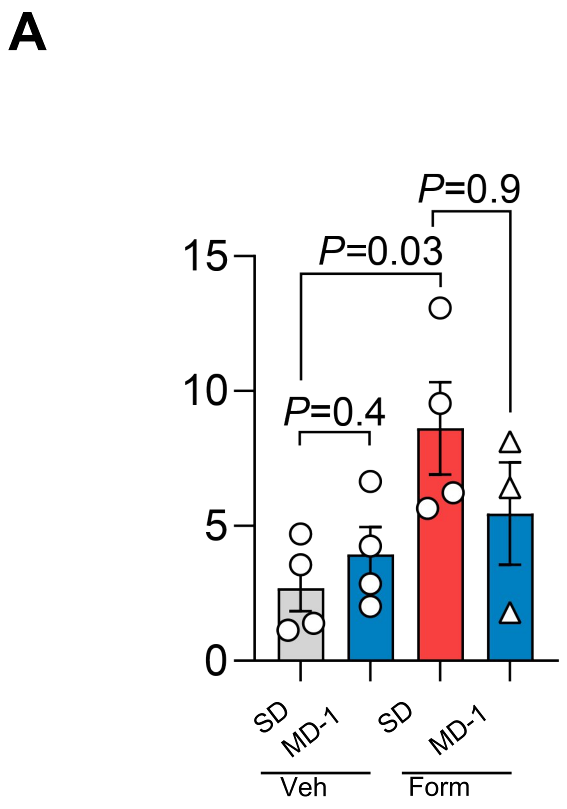

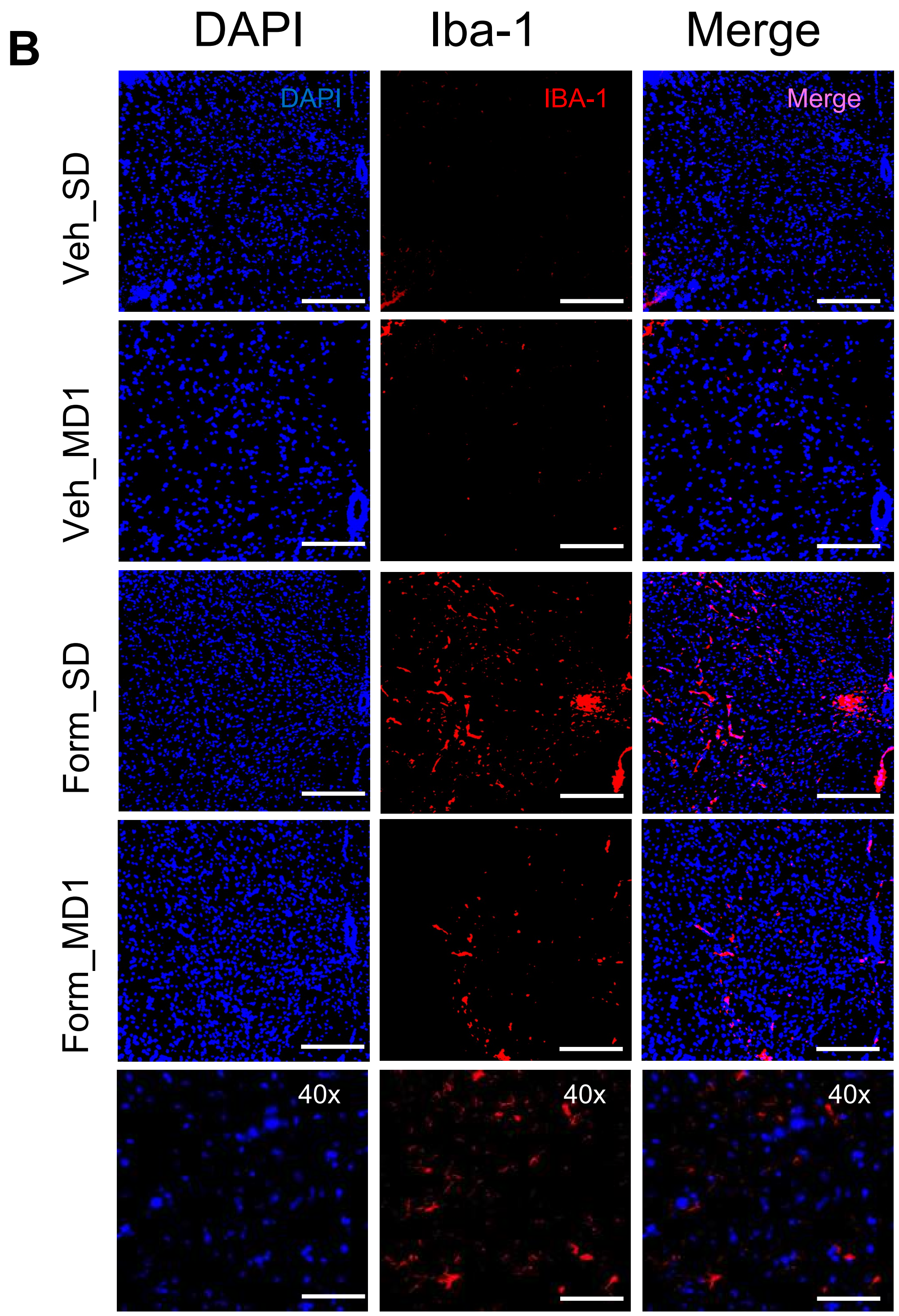

Supplemental Fig. S30

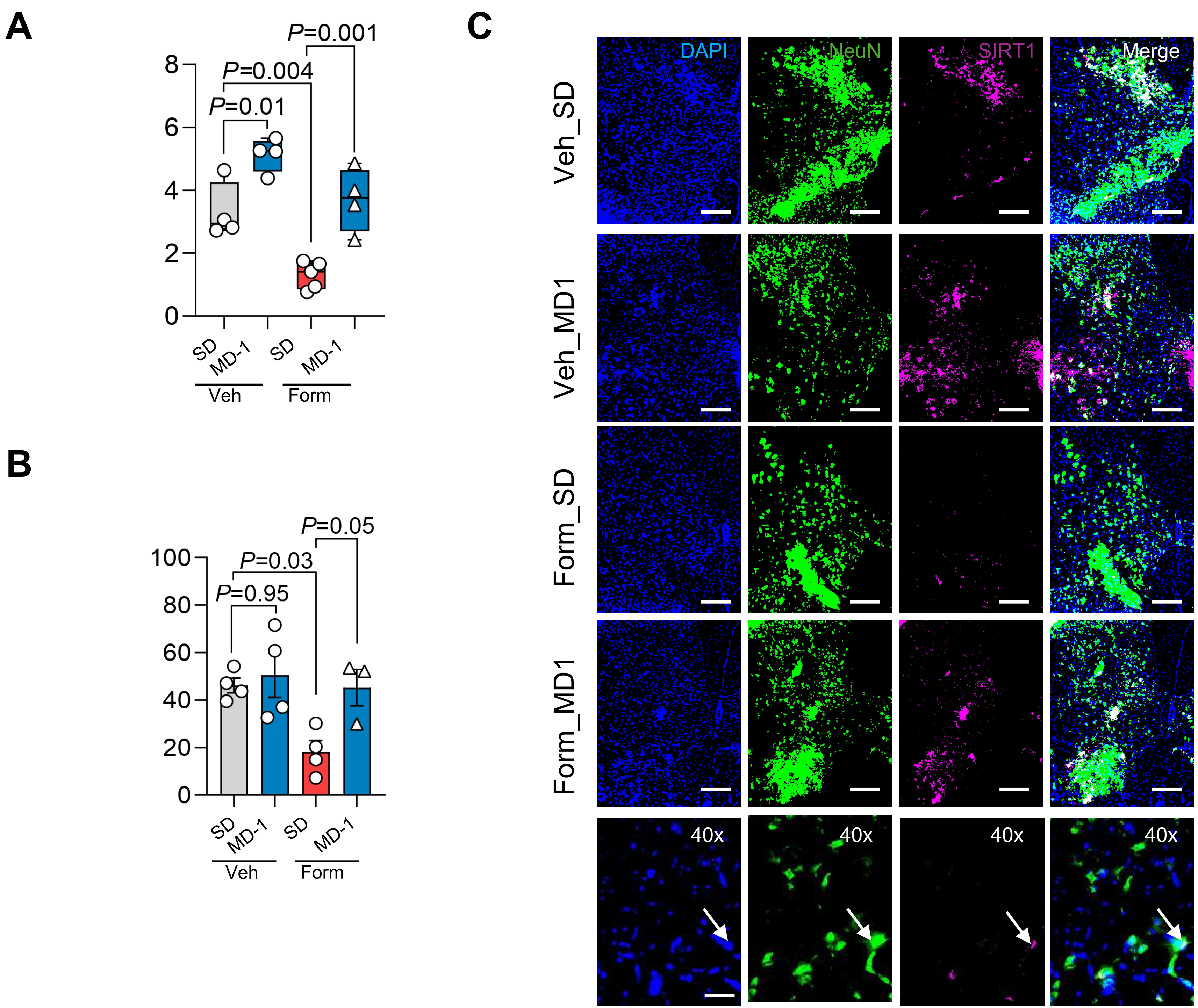

Supplemental Fig. S31

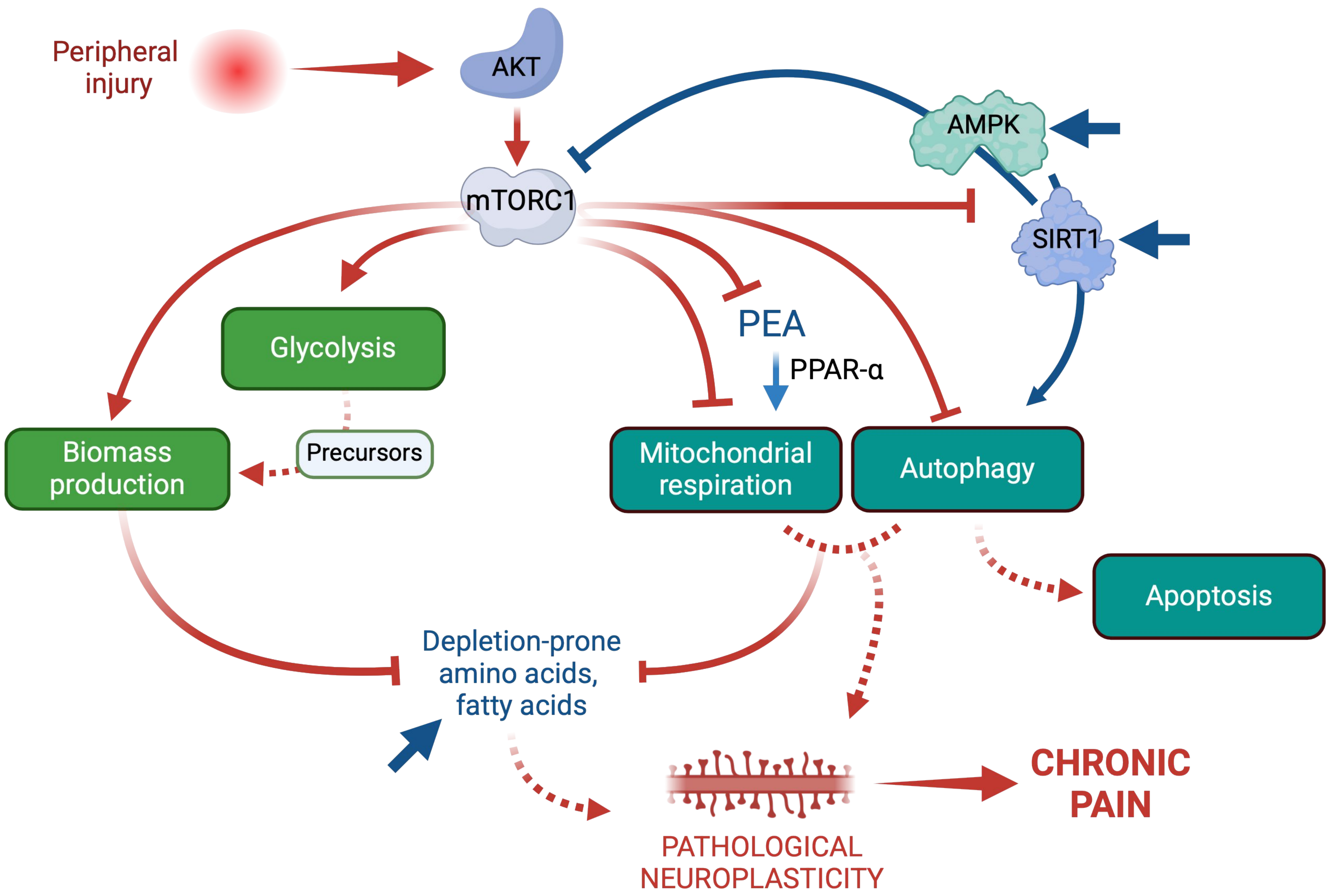

bioRxiv preprint doi: https://doi.org/10.1101/2025.01.30.635746; this version posted June 19, 2025. The copyright holder for this preprint (which was not certified by peer review) is the author/funder, who has granted bioRxiv a license to display the preprint in perpetuity. It is made available under aCC-BY 4.0 International license.

Supplemental Table S1.

A.

Ingredient	SD	MD-1	MD-2	MD-3	MD-4	MD-5
Ala	1.20	2.80	2.00	1.60	2.80	1.20
Thr	0.60	1.76	1.18	0.89	1.76	0.60
Pro	1.90	3.84	2.87	2.39	3.84	1.90
Ser	0.90	1.68	1.29	1.10	1.68	0.90
Leu	2.30	4.85	3.58	2.94	4.85	2.30
Ile	0.70	2.98	1.84	1.27	2.98	0.70
Val	0.90	2.98	1.94	1.42	2.98	0.90
Phe	1.00	2.26	1.63	1.32	2.26	1.00
Tyr	0.50	1.28	0.89	0.70	1.28	0.50
Met	0.50	0.98	0.74	0.62	0.98	0.50
Cys	0.30	0.68	0.49	0.40	0.68	0.30
Trp	0.20	0.76	0.48	0.34	0.76	0.20
Oleic acid	1.10	6.06	3.58	2.34	6.06	1.10
Erucic acid	0.00	0.25	0.13	0.06	0.25	0.00
PEA	0.00	0.08	0.04	0.02	0.00	0.08

B.

	Carbohydrate	Fat	Protein	Energy density	Daily intake (g/100g)
SD	47.0	6.5	19.1	3.2	13.5 ± 0.8
MD-1	35.7	10.2	33.0	3.7	11.8 ± 1.9
MD-2	41.4	9.1	28.4	3.6	ND
MD-3	44.2	7.8	23.7	3.4	ND
MD-4	35.7	10.2	33.0	3.7	ND
MD-5	47.0	6.6	19.1	3.2	ND

Supplemental Table S2.

	Veh_SD		Veh_MD-1			
Compound Id	Mean	SEM	Mean	SEM	Log_2 (FC)	*P*-value
DG(18:1(11Z)/20:5(5Z,8Z,11Z,14Z,17Z)/0:0)	2.50E+04	6.79E+03	8.09E+03	1.65E+03	-1.63	3.63E-02
5-Nonadecyl-1,3-benzenediol	2.56E+05	4.86E+04	9.10E+04	1.82E+04	-1.49	8.48E-03
Isocaucalol isomer	1.17E+06	2.95E+05	4.27E+05	7.37E+04	-1.45	3.46E-02
(S)-Homostachydrine	1.29E+06	1.10E+05	4.87E+05	4.26E+04	-1.41	2.29E-05
Perillic acid	1.14E+06	1.61E+05	4.45E+05	7.99E+04	-1.35	2.06E-03
Palmitoylcarnitine	1.29E+06	1.50E+05	5.10E+05	5.39E+04	-1.34	4.40E-04
9-Decenoylcarnitine	1.18E+04	1.85E+03	5.13E+03	3.62E+02	-1.20	5.62E-03
Indole-3-methyl acetate	3.36E+05	7.15E+04	1.51E+05	1.63E+04	-1.16	3.03E-02
(5Z,8Z)-Tetradecadienoylcarnitine	1.06E+05	1.67E+04	4.76E+04	4.65E+03	-1.15	7.16E-03
3',4',5'-Trimethoxycinnamyl alcohol acetate	6.00E+05	6.64E+04	2.78E+05	3.94E+04	-1.11	9.66E-04
Myristoylcarnitine	2.39E+05	3.02E+04	1.13E+05	1.75E+04	-1.08	2.90E-03
Isolinderanolide	4.65E+05	7.74E+04	2.19E+05	1.67E+04	-1.08	1.16E-02
DL-2-Aminooctanoic acid	1.17E+05	1.43E+04	5.55E+04	5.35E+03	-1.07	1.95E-03
PI(16:0/18:2(9Z,12Z))	1.55E+05	2.50E+04	7.36E+04	1.05E+04	-1.07	1.15E-02
3-Oxododecanoic acid	1.66E+06	2.75E+05	7.98E+05	1.11E+05	-1.05	1.37E-02
Linoleic acid	6.64E+08	1.28E+08	3.26E+08	3.54E+07	-1.03	2.84E-02
Tetradecanedioic acid	4.21E+04	6.59E+03	2.08E+04	2.62E+03	-1.02	1.13E-02
(2R,3R,4R)-2-Amino-4-hydroxy-3-methylpentanoic acid	3.79E+05	5.56E+04	1.91E+05	2.52E+04	-0.99	9.27E-03
(10Z,12Z)-octadeca-10,12-dienoylcarnitine	2.90E+05	4.10E+04	1.47E+05	1.45E+04	-0.98	7.20E-03
Podecdysone b isomer	4.62E+05	6.58E+04	2.38E+05	3.77E+04	-0.95	1.08E-02
Stearoylcarnitine	1.45E+05	1.25E+04	7.68E+04	9.41E+03	-0.92	5.50E-04
SM(d16:1/24:1(15Z))	7.62E+05	1.13E+05	4.05E+05	6.65E+04	-0.91	1.68E-02
2-Cyclohexen-1-one, 3-(15-hydroxypentadecyl)-2,4,4-trimethyl-	3.23E+04	5.56E+03	1.72E+04	2.44E+03	-0.91	2.81E-02
PG(16:0/18:2(9Z,12Z))	3.87E+04	5.38E+03	2.06E+04	2.38E+03	-0.91	9.65E-03

N2-Acetylornithine	6.95E+03	1.13E+03	3.73E+03	4.20E+02	-0.90	2.16E-02
DL-Stachydrine	2.02E+07	1.89E+06	1.09E+07	7.67E+05	-0.89	6.85E-04
Proline betaine	2.20E+06	1.46E+05	1.23E+06	6.22E+04	-0.84	5.31E-05
5-Dodecenoylcarnitine	4.75E+04	7.27E+03	2.68E+04	4.01E+03	-0.83	2.59E-02
Octadeca-2,4,6,8-tetraenoic acid	2.05E+05	3.71E+04	1.19E+05	1.00E+04	-0.79	4.80E-02
PE(P-18:0/22:4(7Z,10Z,13Z,16Z))	3.35E+04	5.74E+03	1.94E+04	3.07E+03	-0.79	4.94E-02
PE(20:2(11Z,14Z)/15:0)	3.88E+05	3.22E+04	2.31E+05	1.93E+04	-0.75	9.10E-04
Adrenic acid	9.05E+06	1.51E+06	5.46E+06	3.98E+05	-0.73	4.39E-02
Tetracosahexaenoic acid	1.66E+06	2.17E+05	1.03E+06	1.37E+05	-0.69	2.74E-02
4-Guanidinobutanoic acid	2.32E+05	2.88E+04	1.46E+05	2.04E+04	-0.68	2.68E-02
9-Hexadecenoylcarnitine	1.85E+05	2.89E+04	1.16E+05	1.29E+04	-0.67	4.96E-02
Ferulic acid 4-O-sulfate	1.83E+06	1.75E+05	1.18E+06	1.57E+05	-0.63	1.51E-02
2-Methoxyestradiol-3-methylether	2.31E+04	2.47E+03	1.54E+04	1.80E+03	-0.58	2.44E-02
PG(18:0/16:0)	4.60E+04	5.31E+03	3.14E+04	3.48E+03	-0.55	3.71E-02
PC(18:2(9Z,12Z)/14:0)	1.98E+06	2.10E+05	1.41E+06	8.64E+04	-0.49	2.83E-02
Argininosuccinic acid	4.79E+03	5.09E+02	3.47E+03	3.42E+02	-0.46	4.96E-02
L-Carnitine	2.22E+08	1.40E+07	1.61E+08	1.06E+07	-0.46	3.52E-03
PC(18:2(9Z,12Z)/16:1(9Z))	4.59E+06	4.18E+05	3.54E+06	2.36E+05	-0.37	4.74E-02
Indoleacrylic acid	3.01E+07	2.46E+06	3.69E+07	1.98E+06	0.29	4.91E-02
8-Hydroxyquinoline	2.74E+06	2.64E+05	3.44E+06	1.99E+05	0.33	4.84E-02
1,5-Naphthalenediamine	2.02E+06	1.84E+05	2.61E+06	1.30E+05	0.37	1.89E-02
Methylindole isomer	5.97E+05	5.26E+04	8.06E+05	5.22E+04	0.43	1.34E-02
3-Indoleacetonitrile	6.23E+05	6.05E+04	8.62E+05	5.16E+04	0.47	8.84E-03
PC(22:5(4Z,7Z,10Z,13Z,16Z)/16:0)	1.26E+07	1.67E+06	1.77E+07	9.69E+05	0.48	2.11E-02
D-(+)-Proline	2.47E+07	1.60E+06	3.46E+07	2.53E+06	0.49	7.33E-03
PE(22:2(13Z,16Z)/18:1(11Z))	3.62E+05	3.49E+04	5.22E+05	4.13E+04	0.53	1.09E-02
LysoPC(24:1(15Z)/0:0)	1.55E+05	2.13E+04	2.27E+05	2.11E+04	0.56	2.87E-02
PE(18:2(9Z,12Z)/18:1(9Z))	1.61E+05	2.21E+04	2.40E+05	2.47E+04	0.58	3.07E-02

Galactitol	8.91E+05	6.94E+04	1.36E+06	1.62E+05	0.62	2.67E-02
Cer(d18:1/24:1(15Z))	1.12E+05	1.54E+04	1.74E+05	1.47E+04	0.64	1.06E-02
Elaidic acid	5.63E+08	9.07E+07	8.87E+08	1.09E+08	0.66	3.93E-02
Deoxyadenosine monophosphate	2.89E+03	3.87E+02	4.60E+03	4.51E+02	0.67	1.25E-02
3-Methylcrotonylglycine	5.48E+04	8.34E+03	8.80E+04	8.73E+03	0.68	1.54E-02
Docosatrienoic acid	8.51E+05	1.31E+05	1.37E+06	1.22E+05	0.69	1.10E-02
PC(18:2(9Z,12Z)/18:1(11Z))	3.13E+07	4.65E+06	5.08E+07	2.63E+06	0.70	2.66E-03
Indole-3-carboxilic acid-sulphate isomer	7.13E+05	1.08E+05	1.19E+06	1.62E+05	0.74	3.07E-02
L-Allothreonine	6.40E+06	4.48E+05	1.09E+07	7.04E+05	0.76	2.61E-04
LysoPC(P-18:0/0:0)	3.06E+05	3.74E+04	5.39E+05	4.85E+04	0.82	2.40E-03
lysops(22:0)	5.58E+06	8.13E+05	9.83E+06	7.56E+05	0.82	1.68E-03
LysoPC(18:1(9Z)/0:0)	5.45E+07	7.92E+06	9.64E+07	1.01E+07	0.82	6.45E-03
Annoglabasin F	1.22E+06	1.82E+05	2.15E+06	2.31E+05	0.82	7.46E-03
3-Hydroxybutyrylcarnitine	2.18E+05	1.70E+04	3.95E+05	4.51E+04	0.86	6.60E-03
PC(18:1(9Z)/15:0)	2.47E+05	2.87E+04	4.53E+05	4.49E+04	0.87	2.75E-03
DG(18:2(9Z,12Z)/18:3(6Z,9Z,12Z)/0:0)	4.21E+04	8.44E+03	7.72E+04	1.29E+04	0.88	4.36E-02
Indoxyl sulfate	6.87E+06	8.78E+05	1.26E+07	1.39E+06	0.88	5.39E-03
LysoPC(22:1(13Z)/0:0)	3.00E+05	4.43E+04	5.62E+05	5.74E+04	0.91	3.39E-03
5-Aminovaleric acid	5.37E+07	4.68E+06	1.01E+08	1.17E+07	0.91	5.58E-03
PI(20:4(8Z,11Z,14Z,17Z)/18:1(9Z))	8.97E+04	1.72E+04	1.71E+05	2.43E+04	0.93	1.90E-02
PE(18:0/18:1(9Z))	3.06E+05	2.98E+04	5.95E+05	3.31E+04	0.96	1.62E-05
LysoPE(0:0/18:1(11Z))	1.01E+06	1.29E+05	2.02E+06	1.99E+05	1.01	1.32E-03
5-Hydroxyindole	1.15E+05	1.32E+04	2.31E+05	2.58E+04	1.01	2.90E-03
N-Acetyltryptophan	3.22E+05	1.61E+04	6.60E+05	6.54E+04	1.04	1.71E-03
Leu asp isomer	1.23E+05	1.05E+04	2.73E+05	3.14E+04	1.15	2.34E-03
Pemoline	5.26E+03	5.88E+02	1.29E+04	1.15E+03	1.29	2.19E-04
Tiglylcarnitine	6.70E+03	7.17E+02	1.75E+04	1.44E+03	1.39	8.75E-05
PE(18:2(9Z,12Z)/24:1(15Z))	4.39E+04	8.23E+03	1.19E+05	2.54E+04	1.44	2.53E-02

PE(16:0/18:1(9Z))	2.64E+04	3.78E+03	7.42E+04	1.44E+04	1.49	1.54E-02
PC(18:1(9Z)/20:2(11Z,14Z))	1.75E+06	4.36E+05	5.63E+06	4.73E+05	1.69	3.15E-05
PE(16:1(9Z)/22:0)	2.59E+05	2.69E+04	8.78E+05	7.72E+04	1.76	9.25E-05
Cresol sulfate isomer	3.84E+06	1.15E+06	1.41E+07	1.56E+06	1.87	2.01E-04
Guanosine	2.44E+04	7.51E+03	1.07E+05	2.73E+04	2.14	2.26E-02
1-Pentanesulfenothioic acid	1.77E+06	4.19E+05	1.39E+07	2.77E+06	2.98	4.45E-03
Inosine	1.31E+06	3.22E+05	1.16E+07	2.32E+06	3.15	4.24E-03
2-(3,4-Dihydroxybenzoyloxy)-4,6-dihydroxybenzoate	3.22E+04	8.81E+03	3.96E+05	9.37E+04	3.62	8.00E-03
PC(18:1(9Z)/16:0)	7.02E+05	2.85E+05	1.59E+07	2.17E+06	4.50	3.76E-04
Xanthine	1.07E+06	4.62E+05	3.17E+07	1.17E+07	4.89	4.04E-02
PE(18:0/22:1(13Z))	4.41E+03	9.38E+02	2.48E+05	3.72E+04	5.81	6.14E-04

Supplemental Table S3.

	Veh_SD		Veh_MD-1			
Compound Id	Mean	SEM	Mean	SEM	Log_2 (FC)	*P*-value
Methionine sulfoxide	3.87E+03	8.83E+02	8.62E+02	1.14E+02	-2.17	7.78E-03
Glutarylcarnitine	5.12E+05	6.26E+04	2.76E+05	3.53E+04	-0.89	5.71E-03
Saccharopine	3.66E+06	4.28E+05	2.01E+06	3.21E+05	-0.87	7.40E-03
N2-Acetylornithine	4.03E+03	3.38E+02	2.31E+03	5.67E+02	-0.80	2.60E-02
Proline betaine	1.18E+05	7.84E+03	7.30E+04	7.45E+03	-0.69	9.38E-04
PS(18:0/18:2(9Z,12Z))	2.22E+06	2.13E+05	1.39E+06	1.00E+05	-0.67	3.88E-03
DL-2-Aminooctanoic acid	1.10E+05	5.12E+03	6.96E+04	5.14E+03	-0.67	5.66E-05
LysoPE(0:0/20:2(11Z,14Z))	4.02E+04	3.34E+03	2.73E+04	3.35E+03	-0.56	1.65E-02
(10Z,12Z)-octadeca-10,12-dienoylcarnitine	4.79E+05	1.99E+04	3.26E+05	2.50E+04	-0.55	3.88E-04
L-Pipecolic acid	4.22E+04	1.18E+03	2.88E+04	1.26E+03	-0.55	2.04E-06
3,4-Dihydroxyhydrocinnamic acid	1.42E+06	1.33E+05	9.90E+05	1.08E+05	-0.52	2.52E-02
D-Glucuronic acid	7.82E+03	3.99E+02	5.49E+03	5.94E+02	-0.51	7.41E-03
PE(18:0/18:3(9Z,12Z,15Z))	3.05E+05	2.05E+04	2.14E+05	1.36E+04	-0.51	2.27E-03
Taurodeoxycholic acid	1.68E+04	1.72E+03	1.19E+04	1.35E+03	-0.50	4.08E-02
Isolinderanolide	2.17E+06	1.35E+05	1.56E+06	1.17E+05	-0.48	3.47E-03
4-Hydroxyproline	2.71E+05	2.03E+04	2.00E+05	1.86E+04	-0.44	2.17E-02
(Ã‚Â±)-2-Hydroxy-4-(methylthio)butanoic acid	1.56E+04	1.09E+03	1.16E+04	1.01E+03	-0.43	1.63E-02
Allantoin	8.62E+05	4.91E+04	6.47E+05	3.12E+04	-0.41	2.38E-03
Stoloniferone c isomer	7.10E+05	5.60E+04	5.44E+05	3.14E+04	-0.38	2.26E-02
LysoPE(20:1(11Z)/0:0)	2.96E+06	1.82E+05	2.38E+06	1.89E+05	-0.32	4.29E-02
Normetanephrine	8.02E+03	5.12E+02	6.46E+03	4.92E+02	-0.31	4.43E-02
L-Fucose	1.00E+05	7.60E+03	8.11E+04	3.89E+03	-0.31	4.18E-02
Pantothenic acid	2.41E+05	8.26E+03	1.96E+05	1.46E+04	-0.30	2.30E-02
Saringosterol isomer	4.04E+06	1.97E+05	3.30E+06	2.20E+05	-0.29	2.68E-02
Uridine diphosphate glucuronic acid	9.93E+04	3.54E+03	8.13E+04	4.74E+03	-0.29	1.02E-02

(2R,3R,4R)-2-Amino-4-hydroxy-3-methylpentanoic acid	4.16E+05	2.12E+04	3.41E+05	1.88E+04	-0.28	1.91E-02
LysoPC(20:2(11Z,14Z)/0:0)	1.56E+05	6.07E+03	1.29E+05	6.25E+03	-0.27	8.87E-03
Uridine diphosphate-N-acetylgalactosamine	3.22E+06	9.54E+04	2.84E+06	1.29E+05	-0.18	3.80E-02
N-Acetyl-1-aspartylglutamic acid	2.14E+08	6.50E+06	1.91E+08	6.66E+06	-0.16	2.65E-02
PE(20:4(6E,8Z,11Z,14Z)+=O(5)/P-18:1(9Z))	2.34E+06	8.05E+04	2.68E+06	1.09E+05	0.20	2.72E-02
Acetylcholine	6.14E+07	2.38E+06	7.05E+07	3.27E+06	0.20	4.32E-02
PE(41:7)	3.75E+05	2.59E+04	4.41E+05	1.17E+04	0.23	3.95E-02
PE(18:0/18:1(9Z))	7.36E+05	3.38E+04	8.67E+05	3.07E+04	0.24	1.17E-02
PE(16:1(9Z)/22:0)	7.03E+05	3.05E+04	8.43E+05	2.90E+04	0.26	4.77E-03
2-Arachidonoyl glycerol	5.19E+06	2.01E+05	6.27E+06	4.05E+05	0.27	4.13E-02
PE(20:4(8Z,11Z,14Z,17Z)/22:6(4Z,7Z,10Z,13Z,16Z,19Z))	1.06E+05	7.78E+03	1.29E+05	5.71E+03	0.28	3.23E-02
2-Methylglutaric acid	2.71E+04	1.10E+03	3.31E+04	1.65E+03	0.29	1.20E-02
Butyric acid	1.95E+04	1.11E+03	2.38E+04	7.97E+02	0.29	6.87E-03
SM(d18:1/18:0)	4.22E+06	3.48E+05	5.28E+06	2.16E+05	0.33	2.05E-02
trans-Aconitic acid	2.32E+06	1.62E+05	3.01E+06	1.61E+05	0.38	8.56E-03
PC(22:5(4Z,7Z,10Z,13Z,16Z)/16:0)	1.11E+06	7.14E+04	1.46E+06	1.08E+05	0.40	1.91E-02
N-Acetylglutamine	2.15E+06	1.02E+05	2.96E+06	2.70E+05	0.46	2.33E-02
PC(16:0/15:0)	4.78E+04	5.67E+03	6.62E+04	4.85E+03	0.47	2.64E-02
Guanidinosuccinic acid	3.22E+03	2.19E+02	4.60E+03	4.17E+02	0.51	1.66E-02
3-Hydroxybutyric acid	1.60E+05	8.45E+03	2.31E+05	2.22E+04	0.52	1.89E-02
Adenine	1.25E+08	1.49E+07	1.87E+08	2.24E+07	0.59	3.99E-02
PE(22:2(13Z,16Z)/20:4(8Z,11Z,14Z,17Z))	1.77E+05	2.36E+04	2.69E+05	1.55E+04	0.60	5.49E-03
Tiglylcarnitine	1.48E+04	1.17E+03	2.39E+04	3.65E+03	0.69	4.80E-02
Deoxycytidine	3.07E+03	4.17E+02	5.06E+03	7.56E+02	0.72	4.49E-02
Diaminopimelic acid	1.37E+03	1.74E+02	2.36E+03	2.57E+02	0.79	8.36E-03
Biotin amide	5.47E+05	3.13E+04	1.01E+06	8.86E+04	0.88	1.46E-03
(S,E)-Zearalenone	1.60E+05	9.83E+03	3.06E+05	3.21E+04	0.93	3.31E-03
Alanylproline	4.12E+03	5.71E+02	7.89E+03	1.17E+03	0.94	1.78E-02

N,N-Dimethylformamide	2.31E+06	8.78E+04	4.60E+06	4.89E+05	1.00	3.07E-03
Enterodiol	1.44E+06	9.31E+04	2.92E+06	2.99E+05	1.02	1.93E-03
Melleolide M	1.92E+05	2.50E+04	4.11E+05	6.67E+04	1.09	1.62E-02
N-alpha-Acetyl-L-citrulline	3.70E+05	1.74E+04	7.91E+05	7.20E+04	1.10	8.66E-04
L-Allothreonine	8.33E+06	4.88E+05	1.92E+07	2.06E+06	1.20	1.58E-03
L-Ergothioneine	1.55E+06	4.48E+04	3.74E+06	5.13E+05	1.27	5.23E-03
2-Hydroxybutyric acid	8.46E+03	1.06E+03	2.20E+04	3.57E+03	1.38	8.15E-03
N1-Methyl-4-pyridone-3-carboxamide	9.73E+04	1.53E+04	2.61E+05	2.13E+04	1.43	4.69E-05
Val asp isomer	2.00E+05	5.67E+03	5.56E+05	4.15E+04	1.48	1.18E-04

Supplemental Table S6.

	Veh_SD		Form_SD			
Compound Id	Mean	SEM	Mean	SEM	Log_2 FC	*P*-value
DG(16:0/18:2(9Z,12Z)/0:0)	1.70E+04	2.56E+03	3.75E+03	8.34E+02	-2.18	9.79E-04
Docosatrienoic acid	1.01E+06	9.66E+04	4.85E+05	5.40E+04	-1.06	5.94E-04
Indole-3-methyl acetate	3.36E+05	7.15E+04	1.67E+05	2.28E+04	-1.01	4.62E-02
5-Nonadecyl-1,3-benzenediol	2.56E+05	4.86E+04	1.38E+05	1.80E+04	-0.89	4.28E-02
Nervonic acid	8.40E+05	1.20E+05	4.72E+05	5.96E+04	-0.83	1.82E-02
Docosadienoate (22:2n6)	6.16E+05	1.06E+05	3.48E+05	4.68E+04	-0.82	3.93E-02
Erucic acid	2.88E+06	4.13E+05	1.69E+06	2.53E+05	-0.77	2.68E-02
Arachidic acid	1.39E+06	2.03E+05	8.19E+05	1.13E+05	-0.76	2.73E-02
10Z-Pentadecenoic acid	1.41E+05	2.00E+04	9.23E+04	9.83E+03	-0.61	4.77E-02
Tetracosahexaenoic acid	1.66E+06	2.17E+05	1.10E+06	8.29E+04	-0.6	3.28E-02
PG(18:0/16:0)	4.60E+04	5.31E+03	3.33E+04	1.46E+03	-0.47	4.22E-02
Taurine	8.47E+07	5.72E+06	1.07E+08	3.13E+06	0.34	4.15E-03
Butyric acid	4.34E+04	4.60E+03	5.61E+04	3.83E+03	0.37	4.79E-02
PC(18:0/18:3(9Z,12Z,15Z))	9.29E+05	9.06E+04	1.25E+06	1.13E+05	0.43	3.77E-02
Pseudouridine	3.61E+06	3.52E+05	4.90E+06	4.28E+05	0.44	3.16E-02
Glutarylcarnitine	2.53E+04	2.08E+03	3.46E+04	3.52E+03	0.45	3.93E-02
Capryloylglycine	1.89E+05	2.31E+04	2.74E+05	3.18E+04	0.54	4.50E-02
3-methylglutarylcarnitine	5.94E+04	5.87E+03	9.89E+04	1.25E+04	0.73	1.35E-02
Aldosterone	4.87E+05	8.77E+04	8.71E+05	8.22E+04	0.84	8.42E-03
SM(d18:1/23:0)	3.78E+04	6.33E+03	7.48E+04	1.22E+04	0.98	1.78E-02
Corticosterone	2.81E+04	5.11E+03	6.68E+04	1.39E+04	1.25	2.39E-02
Indole-3-carboxilic acid-sulphate isomer	7.13E+05	1.08E+05	1.77E+06	2.47E+05	1.31	2.01E-03
PC(18:1(9Z)/16:0)	7.02E+05	2.85E+05	2.36E+06	6.46E+05	1.75	3.59E-02

bioRxiv preprint doi: https://doi.org/10.1101/2025.01.30.635746; this version posted June 19, 2025. The copyright holder for this preprint (which was not certified by peer review) is the author/funder, who has granted bioRxiv a license to display the preprint in perpetuity. It is made available under aCC-BY 4.0 International license.

Supplemental Table S7.

Dependent Variable	Source	Type III Sum of Squares	df	Mean Square	F	Sig.	Partial Eta Squared
Thermal (Ipsilateral)	Corrected Model	554.178	6	92.363	29.654	<.001	0.813
	Intercept	2358.363	1	2358.363	757.175	<.001	0.949
	BW	10.658	1	10.658	3.422	0.072	0.077
	MD-1	481.944	2	240.972	77.366	<.001	0.791
	PFD	22.214	1	22.214	7.132	0.011	0.148
	MD-1 * PFD	17.788	2	8.894	2.856	0.069	0.122
	Error	127.702	41	3.115			
	Total	3129.472	48				
	Corrected Total	681.88	47				
Thermal (Contralateral)	Corrected Model	410.877	6	68.479	13.637	<.001	0.666
	Intercept	3381.238	1	3381.238	673.337	<.001	0.943
	BW	5.594	1	5.594	1.114	0.297	0.026
	MD-1	336.72	2	168.36	33.527	<.001	0.621
	PFD	0.068	1	0.068	0.014	0.908	0
	MD-1 * PFD	0.919	2	0.459	0.091	0.913	0.004
	Error	205.886	41	5.022			
	Total	4024.143	48				
	Corrected Total	616.763	47				
Mechanical (Ipsilateral)	Corrected Model	96.994	6	16.166	65.436	<.001	0.905
	Intercept	367.227	1	367.227	1486.476	<.001	0.973
	BW	0.027	1	0.027	0.109	0.744	0.003
	MD-1	69.051	2	34.526	139.754	<.001	0.872
	PFD	8.695	1	8.695	35.198	<.001	0.462
	MD-1 * PFD	8.428	2	4.214	17.058	<.001	0.454
	Error	10.129	41	0.247			
	Total	482.211	48				
	Corrected Total	107.122	47				
Mechanical (Contralateral)	Corrected Model	67.359	6	11.227	15.798	<.001	0.698
	Intercept	517.257	1	517.257	727.874	<.001	0.947
	BW	1.272	1	1.272	1.79	0.188	0.042
	MD-1	65.028	2	32.514	45.753	<.001	0.691
	PFD	0.562	1	0.562	0.791	0.379	0.019
	MD-1 * PFD	0.261	2	0.13	0.184	0.833	0.009
	Error	29.136	41	0.711			
	Total	630.762	48				
	Corrected Total	96.495	47				

bioRxiv preprint doi: https://doi.org/10.1101/2025.01.30.635746; this version posted June 19, 2025. The copyright holder for this preprint (which was not certified by peer review) is the author/funder, who has granted bioRxiv a license to display the preprint in perpetuity. It is made available under aCC-BY 4.0 International license.

Supplemental Table S8.

	MD-1	MD-2	MD-3	MD-4	MD-5
Phase I	—	—	—	—	—
Phase II	– –	—	—	– –	—
Edema	– –	—	—	—	– –
IPSI T	– – –	– – –	—	—	—
M	– – –	– –	—	—	—
CON T	– – –	– – –	—	—	—
M	– – –	– – –	—	—	—
EPM	– – –	ND	ND	—	– –
NOR	– – –	ND	ND	– – –	—

Supplemental Table S4.

	Form_SD		Veh_SD				Veh_MD-1		Veh_SD				Form_MD-1		Form_SD			
gene_name	Form_SD	SEM	Veh_SD	SEM	Log_2([Form]/[Veh])	*P*-value	Veh_MD-1	SEM	Veh_SD	SEM	Log_2([MD-1]/[SD])	*P*-value	Form_MD-1	SEM	Form_SD	SEM	Log_2([MD-1]/[SD])	*P*-value
Aco2	6.02E+03	2.66E+02	6.97E+03	3.29E+02	-2.10E-01	4.86E-02	6.07E+03	3.74E+02	6.97E+03	3.29E+02	-1.98E-01	1.01E-01	6.93E+03	2.82E+02	6.02E+03	2.66E+02	2.03E-01	4.08E-02
Aldob	4.68E+01	3.25E+00	3.08E+01	4.89E+00	6.01E-01	2.14E-02	4.98E+01	3.48E+00	3.08E+01	4.89E+00	6.92E-01	1.01E-02	3.20E+01	3.14E+00	4.68E+01	3.25E+00	-5.46E-01	8.63E-03
Atp5h	4.81E+03	5.86E+02	5.53E+03	3.03E+02	-2.02E-01	3.07E-01	6.78E+03	8.60E+02	5.53E+03	3.03E+02	2.93E-01	2.19E-01	6.84E+03	3.96E+02	4.81E+03	5.86E+02	5.08E-01	1.90E-02
Atp5j	3.27E+03	2.50E+02	3.50E+03	1.52E+02	-1.01E-01	4.40E-01	4.14E+03	4.01E+02	3.50E+03	1.52E+02	2.40E-01	1.86E-01	4.21E+03	2.46E+02	3.27E+03	2.50E+02	3.67E-01	2.27E-02
Atp5j2	4.21E+03	4.00E+02	4.60E+03	2.32E+02	-1.28E-01	4.23E-01	5.56E+03	7.65E+02	4.60E+03	2.32E+02	2.72E-01	2.78E-01	5.84E+03	3.19E+02	4.21E+03	4.00E+02	4.71E-01	1.05E-02
Atp5k	1.09E+03	9.81E+01	1.01E+03	4.09E+01	1.09E-01	4.81E-01	1.01E+03	1.39E+02	1.01E+03	4.09E+01	-5.14E-03	9.81E-01	1.49E+03	9.77E+01	1.09E+03	9.81E+01	4.45E-01	1.74E-02
Atp5l	2.08E+03	1.78E+02	2.44E+03	1.09E+02	-2.30E-01	1.23E-01	3.04E+03	3.49E+02	2.44E+03	1.09E+02	3.19E-01	1.52E-01	2.89E+03	1.43E+02	2.08E+03	1.78E+02	4.75E-01	5.65E-03
Atp5md	3.18E+03	3.18E+02	3.54E+03	1.70E+02	-1.53E-01	3.53E-01	4.49E+03	5.54E+02	3.54E+03	1.70E+02	3.44E-01	1.52E-01	4.18E+03	2.21E+02	3.18E+03	3.18E+02	3.95E-01	2.97E-02
Atp5mpl	2.20E+03	2.30E+02	2.30E+03	1.18E+02	-6.50E-02	7.06E-01	2.74E+03	3.19E+02	2.30E+03	1.18E+02	2.50E-01	2.46E-01	2.83E+03	1.46E+02	2.20E+03	2.30E+02	3.64E-01	4.76E-02
Atp5	3.0	2.8	3.4	1.7	-1.63E-	3.0	4.01	4.6	3.4	1.7	2.29E-	2.8	4.23	2.2	3.0	2.8	4.71E-	8.7

o	5E+03	2E+02	2E+03	7E+02	01	3E-01	E+03	8E+02	2E+03	7E+02	01	1E-01	E+03	1E+02	5E+03	2E+02	01	2E-03
Atp5pb	6.76E+03	5.87E+02	7.42E+03	2.76E+02	-1.34E-01	3.44E-01	8.65E+03	7.57E+02	7.42E+03	2.76E+02	2.22E-01	1.75E-01	8.21E+03	4.28E+02	6.76E+03	5.87E+02	2.81E-01	7.63E-02
Cox10	2.00E+02	4.36E+00	2.66E+02	4.01E+01	-4.10E-01	1.63E-01	2.18E+02	1.95E+01	2.66E+02	4.01E+01	-2.90E-01	3.12E-01	2.49E+02	3.08E+01	2.00E+02	4.36E+00	3.14E-01	1.76E-01
Cox11	2.61E+02	1.30E+01	2.65E+02	2.03E+01	-1.73E-02	8.99E-01	3.00E+02	1.90E+01	2.65E+02	2.03E+01	1.83E-01	2.28E-01	3.10E+02	3.38E+01	2.61E+02	1.30E+01	2.44E-01	2.29E-01
Cox15	3.45E+02	1.10E+01	4.09E+02	6.21E+01	-2.45E-01	3.55E-01	3.69E+02	2.72E+01	4.09E+02	6.21E+01	-1.47E-01	5.78E-01	3.93E+02	4.71E+01	3.45E+02	1.10E+01	1.87E-01	3.65E-01
Cox18	2.37E+02	9.65E+00	2.59E+02	3.23E+01	-1.27E-01	5.41E-01	2.76E+02	1.20E+01	2.59E+02	3.23E+01	8.87E-02	6.50E-01	2.76E+02	2.92E+01	2.37E+02	9.65E+00	2.17E-01	2.57E-01
Cox19	8.20E+02	4.05E+01	6.69E+02	4.45E+01	2.93E-01	3.14E-02	8.08E+02	4.12E+01	6.69E+02	4.45E+01	2.72E-01	4.51E-02	7.48E+02	6.21E+01	8.20E+02	4.05E+01	-1.32E-01	3.61E-01
Cox20b	4.80E-01	2.11E-01	1.00E+00	6.83E-01	-1.06E+00	4.94E-01	1.31E+00	7.49E-01	1.00E+00	6.83E-01	3.89E-01	7.67E-01	1.03E+00	4.77E-01	4.80E-01	2.11E-01	1.10E+00	3.31E-01
Cox4i1	1.29E+04	7.03E+02	1.04E+04	4.68E+02	3.14E-01	1.59E-02	1.41E+04	1.07E+03	1.04E+04	4.68E+02	4.49E-01	1.45E-02	1.27E+04	8.70E+02	1.29E+04	7.03E+02	-1.61E-02	9.01E-01
Cox5a	2.38E+03	1.32E+02	2.07E+03	1.44E+02	2.01E-01	1.45E-01	2.27E+03	1.39E+02	2.07E+03	1.44E+02	1.38E-01	3.25E-01	2.48E+03	1.62E+02	2.38E+03	1.32E+02	6.18E-02	6.31E-01
Cox5b	3.73E+03	3.49E+02	3.97E+03	1.79E+02	-8.95E-02	5.61E-01	4.30E+03	6.01E+02	3.97E+03	1.79E+02	1.15E-01	6.19E-01	5.27E+03	2.87E+02	3.73E+03	3.49E+02	4.96E-01	7.18E-03
Cox6a1	7.67E+03	9.34E+02	7.80E+03	4.66E+02	-2.41E-02	9.05E-01	8.19E+03	1.19E+03	7.80E+03	4.66E+02	6.95E-02	7.72E-01	1.04E+04	7.20E+02	7.67E+03	9.34E+02	4.45E-01	4.21E-02
Cox	4.3	1.2	4.5	1.5	-4.51E-	9.4	4.65	1.5	4.5	1.5	4.83E-	9.4	6.78	2.6	4.3	1.2	6.37E-	4.3

6a2	6E+00	1E+00	0E+00	9E+00	02	6E-01	E+00	4E+00	0E+00	9E+00	02	6E-01	E+00	6E+00	6E+00	1E+00	01	5E-01
Cox 6b1	4.55E+03	4.40E+02	4.91E+03	2.25E+02	-1.10E-01	4.90E-01	5.49E+03	8.32E+02	4.91E+03	2.25E+02	1.63E-01	5.23E-01	6.32E+03	3.47E+02	4.55E+03	4.40E+02	4.74E-01	1.09E-02
Cox 6b2	2.91E+01	3.09E+00	3.53E+01	5.23E+00	-2.78E-01	3.38E-01	3.02E+01	4.93E+00	3.53E+01	5.23E+00	-2.25E-01	4.93E-01	4.41E+01	3.32E+00	2.91E+01	3.09E+00	5.98E-01	8.14E-03
Cox 6c	5.12E+03	4.51E+02	5.79E+03	3.25E+02	-1.77E-01	2.58E-01	6.39E+03	8.58E+02	5.79E+03	3.25E+02	1.42E-01	5.37E-01	6.91E+03	2.11E+02	5.12E+03	4.51E+02	4.33E-01	8.56E-03
Cox 6c2	3.40E+00	1.12E+00	5.67E+00	1.23E+00	-7.35E-01	2.03E-01	5.05E+00	1.98E+00	5.67E+00	1.23E+00	-1.67E-01	7.97E-01	6.50E+00	2.16E+00	3.40E+00	1.12E+00	9.33E-01	2.41E-01
Cox 7a1	7.78E+01	1.04E+01	7.62E+01	5.95E+00	3.00E-02	8.97E-01	9.08E+01	1.29E+01	7.62E+01	5.95E+00	2.53E-01	3.39E-01	1.04E+02	1.36E+01	7.78E+01	1.04E+01	4.13E-01	1.65E-01
Cox 7a2	3.18E+03	2.42E+02	3.47E+03	1.39E+02	-1.26E-01	3.28E-01	3.87E+03	4.61E+02	3.47E+03	1.39E+02	1.55E-01	4.44E-01	4.10E+03	2.24E+02	3.18E+03	2.42E+02	3.63E-01	2.00E-02
Cox 7a2l	2.53E+03	2.12E+02	2.85E+03	1.16E+02	-1.73E-01	2.20E-01	3.11E+03	3.09E+02	2.85E+03	1.16E+02	1.26E-01	4.59E-01	3.22E+03	1.79E+02	2.53E+03	2.12E+02	3.51E-01	3.15E-02
Cox 7b	2.54E+03	6.88E+02	3.66E+03	6.09E+02	-5.24E-01	2.53E-01	3.01E+03	4.11E+02	3.66E+03	6.09E+02	-2.82E-01	4.00E-01	3.81E+03	1.70E+02	2.54E+03	6.88E+02	5.82E-01	1.28E-01
Cox 7b2	2.66E+01	4.50E+00	2.12E+01	2.98E+00	3.30E-01	3.41E-01	2.81E+01	1.47E+00	2.12E+01	2.98E+00	4.10E-01	7.28E-02	2.50E+01	2.21E+00	2.66E+01	4.50E+00	-8.85E-02	7.61E-01
Cox 7c	5.22E+03	4.05E+02	3.90E+03	1.97E+02	4.22E-01	2.08E-02	6.08E+03	6.37E+02	3.90E+03	1.97E+02	6.40E-01	1.74E-02	4.89E+03	2.75E+02	5.22E+03	4.05E+02	-9.44E-02	5.16E-01
Cox 8a	8.75E+03	5.31E+02	6.88E+03	3.22E+02	3.47E-01	1.61E-02	9.56E+03	8.81E+02	6.88E+03	3.22E+02	4.75E-01	2.74E-02	8.59E+03	4.31E+02	8.75E+03	5.31E+02	-2.59E-02	8.24E-01
Cs	3.0	1.3	3.4	1.4	-1.66E-	4.1	3.10	7.5	3.4	1.4	-1.67E-	4.5	3.44	1.4	3.0	1.3	1.94E-	5.8

	1E+03	6E+02	8E+03	8E+02	01	5E-02	E+03	0E+01	8E+03	8E+02	01	0E-02	E+03	9E+02	1E+03	6E+02	01	9E-02
Cyb5r3	1.17E+03	6.28E+01	1.17E+03	1.49E+02	-3.83E-03	9.85E-01	1.21E+03	4.79E+01	1.17E+03	1.49E+02	4.46E-02	8.22E-01	1.37E+03	1.47E+02	1.17E+03	6.28E+01	2.30E-01	2.47E-01
Dlat	1.66E+03	5.34E+01	1.90E+03	5.90E+01	-2.01E-01	1.30E-02	1.85E+03	4.88E+01	1.90E+03	5.90E+01	-3.90E-02	5.29E-01	1.78E+03	3.28E+01	1.66E+03	5.34E+01	1.04E-01	8.45E-02
Eno3	9.71E+01	4.32E+00	9.20E+01	2.26E+00	7.82E-02	3.20E-01	9.07E+01	7.77E+00	9.20E+01	2.26E+00	-2.04E-02	9.22E-01	8.53E+01	2.04E+00	9.71E+01	4.32E+00	-1.88E-01	3.88E-02
Gapdh	1.56E+03	6.71E+02	1.03E+03	3.81E+02	6.00E-01	5.11E-01	7.71E+02	5.97E+02	1.03E+03	3.81E+02	-4.19E-01	7.23E-01	1.03E+03	6.37E+02	1.56E+03	6.71E+02	-6.00E-01	5.78E-01
Hk3	3.01E+01	1.26E+00	2.15E+01	2.34E+00	4.86E-01	8.90E-03	2.30E+01	3.49E+00	2.15E+01	2.34E+00	9.63E-02	7.29E-01	2.17E+01	2.63E+00	3.01E+01	1.26E+00	-4.76E-01	1.86E-02
Ldhd	2.56E+02	5.02E+00	2.22E+02	1.34E+01	2.10E-01	3.89E-02	2.45E+02	4.81E+00	2.22E+02	1.34E+01	-5.65E-02	1.37E-01	2.35E+02	7.84E+00	2.56E+02	5.02E+00	-1.26E-01	4.34E-02
Mdh2	4.21E+03	1.56E+02	4.05E+03	4.08E+02	5.53E-02	7.29E-01	4.18E+03	2.14E+02	4.05E+03	4.08E+02	4.56E-02	7.85E-01	4.63E+03	3.65E+02	4.21E+03	1.56E+02	1.36E-01	3.30E-01
Ndufa1	8.84E+02	7.07E+01	9.50E+02	4.01E+01	-1.05E-01	4.38E-01	1.35E+03	1.22E+02	9.50E+02	4.01E+01	5.05E-01	2.05E-02	1.13E+03	7.12E+01	8.84E+02	7.07E+01	3.56E-01	3.36E-02
Ndufa11	2.06E+03	1.97E+02	2.28E+03	9.22E+01	-1.43E-01	3.56E-01	3.31E+03	2.98E+02	2.28E+03	9.22E+01	5.41E-01	1.63E-02	2.96E+03	1.88E+02	2.06E+03	1.97E+02	5.21E-01	8.00E-03
Ndufa2	1.84E+03	1.53E+02	1.91E+03	8.39E+01	-5.65E-02	6.86E-01	2.89E+03	2.88E+02	1.91E+03	8.39E+01	5.96E-01	1.80E-02	2.46E+03	1.40E+02	1.84E+03	1.53E+02	4.20E-01	1.36E-02
Ndufa3	2.15E+03	2.94E+02	2.14E+03	1.73E+02	1.89E-03	9.94E-01	4.04E+03	6.14E+02	2.14E+03	1.73E+02	9.16E-01	2.58E-02	3.19E+03	2.50E+02	2.15E+03	2.94E+02	5.70E-01	2.31E-02
Nduf	5.0	4.6	5.7	2.3	-1.86E-	2.1	8.20	7.7	5.7	2.3	5.06E-	2.4	6.59	2.8	5.0	4.6	3.76E-	2.3

a4	8E+03	3E+02	8E+03	9E+02	01	8E-01	E+03	6E+02	8E+03	9E+02	01	7E-02	E+03	6E+02	8E+03	3E+02	01	2E-02
Ndufa5	1.99E+03	1.70E+02	2.14E+03	8.03E+01	-1.04E-01	4.54E-01	3.12E+03	2.91E+02	2.14E+03	8.03E+01	5.44E-01	1.86E-02	2.55E+03	1.43E+02	1.99E+03	1.70E+02	3.56E-01	3.17E-02
Ndufa6	2.60E+03	1.69E+02	2.73E+03	1.52E+02	-6.98E-02	5.83E-01	4.17E+03	4.34E+02	2.73E+03	1.52E+02	6.11E-01	1.94E-02	3.27E+03	1.54E+02	2.60E+03	1.69E+02	3.30E-01	1.53E-02
Ndufa7	1.52E+03	2.04E+02	1.62E+03	7.94E+01	-9.34E-02	6.58E-01	2.78E+03	3.50E+02	1.62E+03	7.94E+01	7.79E-01	2.01E-02	2.24E+03	1.76E+02	1.52E+03	2.04E+02	5.63E-01	2.30E-02
Ndufa9	2.76E+03	1.14E+02	2.93E+03	1.38E+02	-8.61E-02	3.66E-01	4.02E+03	3.07E+02	2.93E+03	1.38E+02	4.57E-01	1.44E-02	3.35E+03	2.39E+02	2.76E+03	1.14E+02	2.79E-01	6.07E-02
Ogdh	2.47E+03	1.08E+02	3.36E+03	3.68E+02	-4.48E-01	4.27E-02	2.80E+03	1.15E+02	3.36E+03	3.68E+02	-2.62E-01	1.77E-01	2.88E+03	1.52E+02	2.47E+03	1.08E+02	2.25E-01	5.25E-02
Pfkfb2	9.64E+02	4.86E+01	8.11E+02	1.40E+01	2.50E-01	1.28E-02	8.39E+02	2.17E+01	8.11E+02	1.40E+01	5.49E-02	3.04E-01	8.88E+02	6.80E+01	9.64E+02	4.86E+01	-1.18E-01	3.89E-01
Pgm2	2.15E+02	1.03E+01	1.91E+02	9.77E+00	1.72E-01	1.20E-01	1.67E+02	1.20E+01	1.91E+02	9.77E+00	-1.94E-01	1.53E-01	2.04E+02	1.53E+01	2.15E+02	1.03E+01	-7.82E-02	5.54E-01
Pgm5	3.89E+02	4.23E+01	3.91E+02	4.29E+01	-7.58E-03	9.74E-01	3.19E+02	1.88E+01	3.91E+02	4.29E+01	-2.92E-01	1.71E-01	3.15E+02	3.23E+01	3.89E+02	4.23E+01	-3.04E-01	1.97E-01
Pink1	5.18E+03	1.01E+02	6.12E+03	3.58E+02	-2.40E-01	3.00E-02	6.06E+03	4.06E+02	6.12E+03	3.58E+02	-1.38E-02	9.14E-01	5.76E+03	2.18E+02	5.18E+03	1.01E+02	1.55E-01	3.64E-02
Pmm2	4.21E+02	1.71E+01	3.86E+02	2.88E+01	1.23E-01	3.33E-01	3.82E+02	3.12E+01	3.86E+02	2.88E+01	-1.79E-02	9.13E-01	3.83E+02	4.36E+01	4.21E+02	1.71E+01	-1.36E-01	4.47E-01
Sdha	9.74E+03	3.50E+02	1.01E+04	7.86E+02	-5.65E-02	6.65E-01	1.13E+04	5.54E+02	1.01E+04	7.86E+02	1.54E-01	2.66E-01	1.09E+04	9.25E+02	9.74E+03	3.50E+02	1.57E-01	2.99E-01
Sdh	5.2	3.3	4.0	1.4	3.70E-	1.6	5.55	4.1	4.0	1.4	4.61E-	1.2	4.88	2.9	5.2	3.3	-9.43E-	4.8

b	1E+03	9E+02	3E+03	0E+02	01	0E-02	E+03	3E+02	3E+03	0E+02	01	7E-02	E+03	6E+02	1E+03	9E+02	02	2E-01
Sdhc	2.04E+03	9.04E+01	2.04E+03	1.65E+02	1.13E-03	9.93E-01	2.22E+03	1.81E+02	2.04E+03	1.65E+02	1.18E-01	4.93E-01	2.15E+03	1.85E+02	2.04E+03	9.04E+01	7.52E-02	6.10E-01
Sdhd	3.07E+03	1.02E+02	2.46E+03	1.04E+02	3.17E-01	1.96E-03	3.28E+03	2.45E+02	2.46E+03	1.04E+02	4.13E-01	1.89E-02	2.82E+03	1.66E+02	3.07E+03	1.02E+02	-1.23E-01	2.32E-01
Suclg2	6.23E+02	3.01E+01	5.98E+02	2.26E+01	5.79E-02	5.31E-01	7.04E+02	4.87E+01	5.98E+02	2.26E+01	2.35E-01	8.97E-02	6.58E+02	4.57E+01	6.23E+02	3.01E+01	8.00E-02	5.33E-01
Uqcrc2	6.23E+03	3.80E+02	5.13E+03	2.99E+02	2.81E-01	4.73E-02	6.74E+03	4.01E+02	5.13E+03	2.99E+02	3.96E-01	9.87E-03	5.61E+03	3.09E+02	6.23E+03	3.80E+02	-1.50E-01	2.40E-01
Uqcrfs1	3.84E+03	1.83E+02	3.29E+03	1.56E+02	2.23E-01	4.54E-02	4.27E+03	3.00E+02	3.29E+03	1.56E+02	3.77E-01	2.11E-02	3.78E+03	2.26E+02	3.84E+03	1.83E+02	-2.17E-02	8.47E-01
Uqcrq	6.59E+03	4.51E+02	4.59E+03	2.42E+02	5.20E-01	4.95E-03	7.35E+03	8.67E+02	4.59E+03	2.42E+02	6.79E-01	2.32E-02	6.23E+03	4.32E+02	6.59E+03	4.51E+02	-8.18E-02	5.74E-01

Supplemental Table S5.

	Form_SD		Veh_SD				Veh_MD-1		Veh_SD				Form_MD-1		Form_SD			
gene_name	Mean	SEM	Mean	SEM	Log_2 (FC)	*P*-value	Mean	SEM	Mean	SEM	Log_2 (FC)	*P*-value	Mean	SEM	Mean	SEM	Log_2 (FC)	*P*-value
Cacna1a	1.85E+03	6.55E+01	1.82E+03	3.78E+02	0.03	9.32E-01	1.39E+03	1.23E+02	1.82E+03	3.78E+02	-0.39	3.21E-01	1.51E+03	2.17E+02	1.85E+03	6.55E+01	-0.29	1.85E-01
Cacna1b	1.34E+03	6.68E+01	1.82E+03	3.36E+02	-0.44	2.21E-01	1.31E+03	1.45E+02	1.82E+03	3.36E+02	-0.47	2.12E-01	1.41E+03	2.33E+02	1.34E+03	6.68E+01	0.06	8.09E-01
Cacna1c	1.04E+03	3.73E+01	1.01E+03	1.70E+02	0.03	9.00E-01	8.14E+02	6.86E+01	1.01E+03	1.70E+02	-0.32	3.14E-01	8.85E+02	1.12E+02	1.04E+03	3.73E+01	-0.23	2.43E-01
Cacna1d	7.90E+02	4.37E+01	8.49E+02	1.06E+02	-0.10	6.22E-01	6.78E+02	6.66E+01	8.49E+02	1.06E+02	-0.33	2.07E-01	7.54E+02	9.72E+01	7.90E+02	4.37E+01	-0.07	7.47E-01
Cacna1e	2.70E+03	1.34E+02	2.86E+03	3.80E+02	-0.08	7.06E-01	2.31E+03	1.86E+02	2.86E+03	3.80E+02	-0.31	2.32E-01	2.40E+03	2.33E+02	2.70E+03	1.34E+02	-0.17	2.87E-01
Cacna1f	1.27E+01	1.10E+00	1.18E+01	2.54E+00	0.10	7.59E-01	1.05E+01	1.57E+00	1.18E+01	2.54E+00	-0.17	6.67E-01	9.32E+00	2.04E+00	1.27E+01	1.10E+00	-0.45	1.83E-01
Cacna1i	8.52E+02	2.90E+01	7.18E+02	1.57E+02	0.25	4.39E-01	5.03E+02	4.88E+01	7.18E+02	1.57E+02	-0.51	2.40E-01	5.57E+02	8.16E+01	8.52E+02	2.90E+01	-0.61	1.36E-02
Cacna2d2	1.11E+03	3.97E+01	1.05E+03	2.24E+02	0.08	7.99E-01	8.15E+02	7.77E+01	1.05E+03	2.24E+02	-0.37	3.58E-01	8.73E+02	1.29E+02	1.11E+03	3.97E+01	-0.35	1.29E-01
Cacna2d3	1.34E+03	4.92E+01	1.13E+03	1.25E+02	0.25	1.66E-01	1.19E+03	6.65E+01	1.13E+03	1.25E+02	0.08	6.68E-01	1.12E+03	1.10E+02	1.34E+03	4.92E+01	-0.25	1.17E-01
Cacnb1	4.39E+0	2.38E+0	5.59E+0	1.04E+0	-0.35	3.06E-	4.32E+0	4.03E+0	5.59E+0	1.04E+0	-0.37	2.95E-	4.80E+0	8.06E+0	4.39E+0	2.38E+0	0.13	6.43E-

	2	1	2	2		01	2	1	2	2		01	2	1	2	1		01
Cacnb2	9.75E+02	5.50E+01	8.64E+02	8.19E+01	0.17	2.91E-01	8.33E+02	4.22E+01	8.64E+02	8.19E+01	-0.05	7.43E-01	8.69E+02	7.23E+01	9.75E+02	5.50E+01	-0.17	2.70E-01
Cacnb3	4.67E+02	1.32E+01	4.38E+02	7.96E+01	0.09	7.33E-01	3.49E+02	3.42E+01	4.38E+02	7.96E+01	-0.33	3.42E-01	3.52E+02	5.93E+01	4.67E+02	1.32E+01	-0.41	1.13E-01
Cacnb4	5.51E+03	4.04E+02	5.02E+03	2.76E+02	0.13	3.42E-01	5.13E+03	2.80E+02	5.02E+03	2.76E+02	0.03	7.84E-01	5.18E+03	2.76E+02	5.51E+03	4.04E+02	-0.09	5.17E-01
Cacng1	2.62E+00	4.22E-01	2.17E+00	9.10E-01	0.28	6.62E-01	2.05E+00	6.15E-01	2.17E+00	9.10E-01	-0.08	9.15E-01	2.08E+00	6.71E-01	2.62E+00	4.22E-01	-0.34	5.09E-01
Cacng2	6.50E+02	5.05E+01	1.05E+03	2.39E+02	-0.69	1.61E-01	7.19E+02	7.66E+01	1.05E+03	2.39E+02	-0.54	2.40E-01	8.17E+02	1.46E+02	6.50E+02	5.05E+01	0.33	3.18E-01
Cacng3	2.06E+02	4.95E+00	1.96E+02	3.58E+01	0.07	7.86E-01	1.68E+02	1.22E+01	1.96E+02	3.58E+01	-0.22	4.92E-01	1.61E+02	2.59E+01	2.06E+02	4.95E+00	-0.35	1.47E-01
Cacng4	4.84E+02	8.86E+00	4.30E+02	9.71E+01	0.17	6.04E-01	3.44E+02	3.51E+01	4.30E+02	9.71E+01	-0.32	4.37E-01	3.19E+02	3.69E+01	4.84E+02	8.86E+00	-0.60	5.76E-03
Cacng5	3.04E+02	8.38E+00	2.70E+02	6.14E+01	0.17	6.09E-01	1.87E+02	1.27E+01	2.70E+02	6.14E+01	-0.53	2.40E-01	1.93E+02	2.25E+01	3.04E+02	8.38E+00	-0.65	3.12E-03
Cacng6	1.91E+00	9.89E-01	1.50E+00	4.28E-01	0.35	7.13E-01	1.63E+00	9.46E-01	1.50E+00	4.28E-01	0.12	9.02E-01	5.00E-01	2.24E-01	1.91E+00	9.89E-01	-1.94	2.17E-01
Cacng7	2.47E+03	5.48E+01	2.19E+03	4.01E+02	0.17	5.17E-01	1.91E+03	1.28E+02	2.19E+03	4.01E+02	-0.20	5.33E-01	1.80E+03	2.59E+02	2.47E+03	5.48E+01	-0.46	4.84E-02
Cacng8	3.72E+02	1.13E+01	3.14E+02	4.79E+01	0.24	2.87E-01	2.50E+02	1.69E+01	3.14E+02	4.79E+01	-0.33	2.55E-01	2.56E+02	3.81E+01	3.72E+02	1.13E+01	-0.54	2.77E-02
Cept1	1.68E+0	9.01E+0	1.42E+0	7.90E+0	0.24	5.71E-	1.34E+0	1.24E+0	1.42E+0	7.90E+0	-0.09	5.66E-	1.51E+0	9.17E+0	1.68E+0	9.01E+0	-0.16	2.04E-

	3	1	3	1		02	3	2	3	1		01	3	1	3	1		01
Cers5	1.06E+03	2.91E+01	8.84E+02	6.35E+01	0.26	4.32E-02	8.26E+02	6.42E+01	8.84E+02	6.35E+01	-0.10	5.38E-01	9.13E+02	5.92E+01	1.06E+03	2.91E+01	-0.21	6.58E-02
Dnah10	7.86E+01	7.44E+00	7.62E+01	1.33E+01	0.04	8.79E-01	7.55E+01	9.54E+00	7.62E+01	1.33E+01	-0.01	9.66E-01	8.39E+01	1.99E+01	7.86E+01	7.44E+00	0.10	8.09E-01
Dnah2	1.19E+02	7.48E+00	1.02E+02	1.35E+01	0.22	3.04E-01	1.10E+02	1.02E+01	1.02E+02	1.35E+01	0.11	6.51E-01	1.10E+02	2.39E+01	1.19E+02	7.48E+00	-0.11	7.49E-01
Dnah6	1.19E+02	1.49E+01	1.25E+02	1.35E+01	-0.07	7.87E-01	1.08E+02	1.67E+01	1.25E+02	1.35E+01	-0.20	4.64E-01	1.16E+02	2.28E+01	1.19E+02	1.49E+01	-0.04	9.05E-01
Dnah7a	1.87E+02	1.70E+01	1.47E+02	1.43E+01	0.35	1.02E-01	1.57E+02	7.67E+00	1.47E+02	1.43E+01	0.09	5.61E-01	1.48E+02	1.15E+01	1.87E+02	1.70E+01	-0.34	9.01E-02
Dnah7b	3.76E+02	2.56E+01	3.42E+02	3.60E+01	0.14	4.60E-01	3.88E+02	4.59E+01	3.42E+02	3.60E+01	0.18	4.54E-01	3.23E+02	6.17E+01	3.76E+02	2.56E+01	-0.22	4.49E-01
Dnah7c	4.03E+01	3.60E+00	3.55E+01	2.84E+00	0.18	3.20E-01	3.32E+01	4.37E+00	3.55E+01	2.84E+00	-0.10	6.71E-01	3.03E+01	4.59E+00	4.03E+01	3.60E+00	-0.41	1.20E-01
Dync1h1	1.08E+04	2.62E+02	8.82E+03	1.74E+03	0.29	3.16E-01	7.44E+03	6.28E+02	8.82E+03	1.74E+03	-0.24	4.84E-01	7.41E+03	1.19E+03	1.08E+04	2.62E+02	-0.54	3.56E-02
Dync1i2	4.31E+03	3.25E+02	3.89E+03	2.27E+02	0.15	3.19E-01	4.13E+03	1.92E+02	3.89E+03	2.27E+02	0.09	4.42E-01	4.05E+03	2.65E+02	4.31E+03	3.25E+02	-0.09	5.41E-01
Dync1li1	1.17E+03	4.10E+01	1.35E+03	1.61E+02	-0.20	3.35E-01	1.30E+03	5.56E+01	1.35E+03	1.61E+02	-0.05	7.95E-01	1.34E+03	1.25E+02	1.17E+03	4.10E+01	0.19	2.45E-01
Dync2h1	1.73E+03	1.36E+02	1.90E+03	9.16E+01	-0.14	3.13E-01	1.87E+03	7.66E+01	1.90E+03	9.16E+01	-0.03	7.82E-01	2.00E+03	1.59E+02	1.73E+03	1.36E+02	0.21	2.22E-01
Dync2li1	3.48E+0	1.79E+0	2.88E+0	1.51E+0	0.27	2.82E-	3.56E+0	1.74E+0	2.88E+0	1.51E+0	0.31	1.50E-	3.16E+0	2.80E+0	3.48E+0	1.79E+0	-0.14	3.59E-

	2	1	2	1		02	2	1	2	1		02	2	1	2	1		01
Dynll1	4.07E+03	2.63E+02	3.36E+03	1.30E+02	0.28	4.50E-02	3.84E+03	2.96E+02	3.36E+03	1.30E+02	0.19	1.84E-01	4.16E+03	2.45E+02	4.07E+03	2.63E+02	0.03	7.99E-01
Dynll2	1.09E+04	2.36E+02	1.37E+04	2.00E+03	-0.33	2.22E-01	1.36E+04	7.31E+02	1.37E+04	2.00E+03	-0.01	9.69E-01	1.35E+04	1.55E+03	1.09E+04	2.36E+02	0.31	1.58E-01
Dynlrb1	5.47E+03	3.81E+02	3.96E+03	2.47E+02	0.47	9.49E-03	5.53E+03	5.22E+02	3.96E+03	2.47E+02	0.48	2.92E-02	4.55E+03	2.63E+02	5.47E+03	3.81E+02	-0.26	8.01E-02
Dynlt1a	6.02E+01	9.27E+00	4.97E+01	1.33E+01	0.28	5.33E-01	6.86E+01	1.06E+01	4.97E+01	1.33E+01	0.47	2.93E-01	7.98E+01	1.19E+01	6.02E+01	9.27E+00	0.41	2.23E-01
Dynlt1b	2.69E+02	1.66E+01	1.71E+02	1.84E+01	0.65	2.75E-03	2.41E+02	3.27E+01	1.71E+02	1.84E+01	0.49	1.02E-01	1.89E+02	2.79E+01	2.69E+02	1.66E+01	-0.51	3.82E-02
Dynlt1c	1.24E+02	9.27E+00	9.85E+01	1.30E+01	0.33	1.50E-01	1.22E+02	1.45E+01	9.85E+01	1.30E+01	0.30	2.65E-01	8.94E+01	1.08E+01	1.24E+02	9.27E+00	-0.47	3.74E-02
Dynlt1f	1.48E+02	7.44E+00	8.35E+01	1.11E+01	0.83	9.83E-04	1.34E+02	2.08E+01	8.35E+01	1.11E+01	0.68	6.74E-02	1.03E+02	5.57E+00	1.48E+02	7.44E+00	-0.53	7.86E-04
Dynlt3	5.31E+03	2.72E+02	4.61E+03	2.30E+02	0.20	7.76E-02	5.53E+03	4.21E+02	4.61E+03	2.30E+02	0.26	9.14E-02	4.87E+03	2.45E+02	5.31E+03	2.72E+02	-0.12	2.63E-01
Dynlt4	3.07E+01	4.13E+00	2.17E+01	2.50E+00	0.50	9.58E-02	3.36E+01	4.04E+00	2.17E+01	2.50E+00	0.63	3.55E-02	3.16E+01	6.56E+00	3.07E+01	4.13E+00	0.04	9.13E-01
Dynlt5	1.54E+01	2.23E+00	1.15E+01	1.09E+00	0.42	1.56E-01	1.54E+01	1.26E+00	1.15E+01	1.09E+00	0.42	4.14E-02	1.39E+01	3.05E+00	1.54E+01	2.23E+00	-0.15	6.90E-01
Fdft1	3.91E+03	2.75E+02	3.29E+03	1.90E+02	0.25	9.46E-02	3.35E+03	2.45E+02	3.29E+03	1.90E+02	0.03	8.31E-01	3.94E+03	2.21E+02	3.91E+03	2.75E+02	0.01	9.25E-01
Gabarap	5.14E+0	3.44E+0	4.01E+0	1.59E+0	0.36	2.07E-	4.16E+0	4.87E+0	4.01E+0	1.59E+0	0.05	7.75E-	4.63E+0	2.43E+0	5.14E+0	3.44E+0	-0.15	2.62E-

	3	2	3	2		02	3	2	3	2		01	3	2	3	2		01
Gabarapl1	8.40E+03	2.80E+02	8.19E+03	5.47E+02	0.04	7.38E-01	8.27E+03	4.78E+02	8.19E+03	5.47E+02	0.01	9.14E-01	8.81E+03	7.02E+02	8.40E+03	2.80E+02	0.07	6.11E-01
Gabarapl2	4.21E+03	1.97E+02	3.38E+03	1.68E+02	0.32	9.82E-03	4.10E+03	3.18E+02	3.38E+03	1.68E+02	0.28	8.22E-02	3.96E+03	1.94E+02	4.21E+03	1.97E+02	-0.09	3.91E-01
Gabbr1	6.62E+03	6.53E+01	8.20E+03	1.13E+03	-0.31	2.20E-01	7.19E+03	4.09E+02	8.20E+03	1.13E+03	-0.19	4.30E-01	7.49E+03	8.05E+02	6.62E+03	6.53E+01	0.18	3.31E-01
Gabbr2	8.11E+02	3.45E+01	1.55E+03	4.07E+02	-0.93	1.31E-01	9.11E+02	8.93E+01	1.55E+03	4.07E+02	-0.76	1.83E-01	1.17E+03	1.93E+02	8.11E+02	3.45E+01	0.53	1.23E-01
Gabra1	2.20E+03	1.57E+02	2.03E+03	8.41E+01	0.12	3.68E-01	1.93E+03	1.47E+02	2.03E+03	8.41E+01	-0.08	5.44E-01	2.04E+03	8.33E+01	2.20E+03	1.57E+02	-0.11	3.72E-01
Gabra2	5.86E+03	5.91E+02	5.69E+03	1.78E+02	0.04	7.85E-01	5.76E+03	2.51E+02	5.69E+03	1.78E+02	0.02	8.22E-01	6.35E+03	4.20E+02	5.86E+03	5.91E+02	0.12	5.19E-01
Gabra3	4.50E+03	2.93E+02	4.14E+03	1.91E+02	0.12	3.37E-01	3.95E+03	2.60E+02	4.14E+03	1.91E+02	-0.07	5.65E-01	4.35E+03	2.68E+02	4.50E+03	2.93E+02	-0.05	7.23E-01
Gabra5	1.09E+03	8.44E+01	1.16E+03	7.31E+01	-0.09	5.39E-01	1.09E+03	5.58E+01	1.16E+03	7.31E+01	-0.10	4.26E-01	1.12E+03	7.23E+01	1.09E+03	8.44E+01	0.03	8.33E-01
Gabrb1	2.81E+03	1.88E+02	3.07E+03	1.73E+02	-0.13	3.34E-01	2.72E+03	1.39E+02	3.07E+03	1.73E+02	-0.17	1.47E-01	3.14E+03	2.20E+02	2.81E+03	1.88E+02	0.16	2.90E-01
Gabrb2	1.04E+03	4.92E+01	1.13E+03	6.55E+01	-0.12	3.18E-01	1.14E+03	5.15E+01	1.13E+03	6.55E+01	0.02	8.40E-01	1.17E+03	7.42E+01	1.04E+03	4.92E+01	0.17	1.73E-01
Gabrb3	3.37E+03	1.71E+02	3.65E+03	2.84E+02	-0.11	4.27E-01	3.58E+03	1.75E+02	3.65E+03	2.84E+02	-0.03	8.44E-01	3.61E+03	2.57E+02	3.37E+03	1.71E+02	0.10	4.64E-01
Gabrd	1.26E+0	1.25E+0	1.83E+0	2.36E+0	-0.54	6.71E-	1.53E+0	3.24E+0	1.83E+0	2.36E+0	-0.26	4.63E-	1.36E+0	3.49E+0	1.26E+0	1.25E+0	0.10	8.09E-

	1	0	1	0		02	1	0	1	0		01	1	0	1	0		01
Gabre	2.59E+01	3.85E+00	3.60E+01	6.58E+00	-0.47	2.23E-01	3.13E+01	3.93E+00	3.60E+01	6.58E+00	-0.20	5.53E-01	2.75E+01	5.15E+00	2.59E+01	3.85E+00	0.08	8.16E-01
Gabrg1	2.57E+03	1.29E+02	2.09E+03	9.37E+01	0.30	1.31E-02	2.12E+03	2.13E+02	2.09E+03	9.37E+01	0.02	8.95E-01	2.19E+03	7.83E+01	2.57E+03	1.29E+02	-0.23	3.38E-02
Gabrg2	4.11E+03	2.79E+02	3.43E+03	1.58E+02	0.26	7.04E-02	3.26E+03	3.48E+02	3.43E+03	1.58E+02	-0.08	6.59E-01	3.77E+03	1.66E+02	4.11E+03	2.79E+02	-0.12	3.27E-01
Gabrg3	3.22E+02	2.01E+01	4.02E+02	4.39E+01	-0.32	1.45E-01	3.38E+02	1.50E+01	4.02E+02	4.39E+01	-0.25	2.15E-01	3.37E+02	2.53E+01	3.22E+02	2.01E+01	0.07	6.54E-01
Gabrr1	2.49E+00	3.07E-01	4.33E+00	8.43E-01	-0.80	8.37E-02	2.66E+00	8.82E-01	4.33E+00	8.43E-01	-0.71	1.99E-01	3.68E+00	1.54E+00	2.49E+00	3.07E-01	0.56	4.79E-01
Gabrr2	2.32E+01	3.30E+00	1.83E+01	3.48E+00	0.34	3.32E-01	2.41E+01	6.72E+00	1.83E+01	3.48E+00	0.39	4.73E-01	2.31E+01	2.11E+00	2.32E+01	3.30E+00	-0.01	9.72E-01
Gria1	1.63E+03	7.25E+01	2.08E+03	3.33E+02	-0.35	2.39E-01	1.63E+03	1.54E+02	2.08E+03	3.33E+02	-0.35	2.57E-01	1.89E+03	3.28E+02	1.63E+03	7.25E+01	0.22	4.64E-01
Gria2	1.07E+04	6.88E+02	9.04E+03	5.49E+02	0.24	9.78E-02	9.48E+03	3.34E+02	9.04E+03	5.49E+02	0.07	5.12E-01	9.03E+03	6.18E+02	1.07E+04	6.88E+02	-0.24	1.09E-01
Gria3	3.33E+03	1.75E+02	2.91E+03	2.02E+02	0.20	1.44E-01	3.36E+03	1.18E+02	2.91E+03	2.02E+02	0.21	8.84E-02	3.48E+03	2.23E+02	3.33E+03	1.75E+02	0.06	6.18E-01
Gria4	6.02E+03	3.50E+02	5.40E+03	2.18E+02	0.15	1.74E-01	6.15E+03	2.00E+02	5.40E+03	2.18E+02	0.19	3.06E-02	6.18E+03	4.16E+02	6.02E+03	3.50E+02	0.04	7.75E-01
Grid1	7.35E+02	1.64E+01	5.57E+02	1.20E+02	0.40	2.00E-01	5.53E+02	3.83E+01	5.57E+02	1.20E+02	-0.01	9.74E-01	4.47E+02	6.68E+01	7.35E+02	1.64E+01	-0.72	6.72E-03
Grid2	4.64E+0	1.01E+0	4.26E+0	4.85E+0	0.12	4.79E-	4.03E+0	2.21E+0	4.26E+0	4.85E+0	-0.08	6.84E-	3.97E+0	4.42E+0	4.64E+0	1.01E+0	-0.22	1.96E-

	2	1	2	1		01	2	1	2	1		01	2	1	2	1		01
Grid2ip	1.80E+02	8.91E+00	1.71E+02	3.59E+01	0.07	8.15E-01	1.60E+02	1.43E+01	1.71E+02	3.59E+01	-0.10	7.79E-01	1.41E+02	2.38E+01	1.80E+02	8.91E+00	-0.35	1.74E-01
Grifin	1.74E+01	2.17E+00	1.18E+01	7.49E-01	0.56	5.03E-02	1.45E+01	3.32E+00	1.18E+01	7.49E-01	0.30	4.60E-01	2.07E+01	3.31E+00	1.74E+01	2.17E+00	0.25	4.28E-01
Grik1	3.09E+02	1.39E+01	2.99E+02	4.20E+01	0.05	8.26E-01	2.60E+02	1.93E+01	2.99E+02	4.20E+01	-0.20	4.36E-01	2.91E+02	3.62E+01	3.09E+02	1.39E+01	-0.08	6.67E-01
Grik2	1.92E+03	1.27E+02	1.58E+03	6.57E+01	0.28	4.71E-02	1.57E+03	8.20E+01	1.58E+03	6.57E+01	0.00	9.73E-01	1.67E+03	1.16E+02	1.92E+03	1.27E+02	-0.20	1.78E-01
Grik3	7.96E+02	2.28E+01	6.03E+02	1.37E+02	0.40	2.23E-01	6.03E+02	4.45E+01	6.03E+02	1.37E+02	0.00	9.99E-01	4.77E+02	7.31E+01	7.96E+02	2.28E+01	-0.74	6.00E-03
Grik4	4.03E+02	1.04E+01	3.58E+02	5.16E+01	0.17	4.29E-01	3.51E+02	2.66E+01	3.58E+02	5.16E+01	-0.03	9.15E-01	3.39E+02	4.86E+01	4.03E+02	1.04E+01	-0.25	2.53E-01
Grik5	1.33E+03	4.76E+01	1.25E+03	2.28E+02	0.09	7.41E-01	1.01E+03	9.60E+01	1.25E+03	2.28E+02	-0.30	3.70E-01	1.04E+03	1.49E+02	1.33E+03	4.76E+01	-0.35	1.14E-01
Grin1	2.05E+03	7.65E+01	2.77E+03	5.75E+02	-0.44	2.66E-01	2.02E+03	1.81E+02	2.77E+03	5.75E+02	-0.46	2.58E-01	2.34E+03	3.41E+02	2.05E+03	7.65E+01	0.19	4.43E-01
Grin2a	1.07E+03	5.77E+01	1.39E+03	1.84E+02	-0.37	1.53E-01	1.21E+03	7.82E+01	1.39E+03	1.84E+02	-0.19	4.14E-01	1.35E+03	1.36E+02	1.07E+03	5.77E+01	0.33	1.04E-01
Grin2b	6.07E+02	2.40E+01	9.12E+02	1.61E+02	-0.59	1.17E-01	7.13E+02	6.69E+01	9.12E+02	1.61E+02	-0.35	2.93E-01	8.34E+02	1.04E+02	6.07E+02	2.40E+01	0.46	8.03E-02
Grin2c	3.09E+02	1.98E+01	4.00E+02	5.35E+01	-0.38	1.56E-01	3.11E+02	2.73E+01	4.00E+02	5.35E+01	-0.37	1.77E-01	3.28E+02	6.95E+01	3.09E+02	1.98E+01	0.09	7.93E-01
Grin2d	7.51E+0	2.99E+0	7.16E+0	1.44E+0	0.07	8.24E-	6.49E+0	4.85E+0	7.16E+0	1.44E+0	-0.14	6.74E-	6.12E+0	7.83E+0	7.51E+0	2.99E+0	-0.29	1.47E-

	2	1	2	2		01	2	1	2	2		01	2	1	2	1		01
Grin3a	9.64E+02	1.66E+01	1.18E+03	1.66E+02	-0.30	2.46E-01	9.37E+02	5.85E+01	1.18E+03	1.66E+02	-0.34	2.10E-01	1.00E+03	1.04E+02	9.64E+02	1.66E+01	0.06	7.26E-01
Grin3b	4.27E+01	4.51E+00	7.28E+01	1.28E+01	-0.77	6.68E-02	4.34E+01	4.43E+00	7.28E+01	1.28E+01	-0.75	7.17E-02	6.72E+01	1.24E+01	4.27E+01	4.51E+00	0.66	1.11E-01
Grina	5.60E+03	1.32E+02	4.98E+03	9.39E+02	0.17	5.41E-01	5.18E+03	2.72E+02	4.98E+03	9.39E+02	0.06	8.43E-01	4.46E+03	5.97E+02	5.60E+03	1.32E+02	-0.33	1.16E-01
Grip1	4.56E+02	1.14E+01	3.41E+02	4.79E+01	0.42	6.18E-02	4.40E+02	2.15E+01	3.41E+02	4.79E+01	0.37	1.00E-01	4.19E+02	3.53E+01	4.56E+02	1.14E+01	-0.12	3.62E-01
Grm1	8.68E+02	2.33E+01	6.99E+02	1.15E+02	0.31	2.05E-01	6.70E+02	3.31E+01	6.99E+02	1.15E+02	-0.06	8.21E-01	6.15E+02	6.26E+01	8.68E+02	2.33E+01	-0.50	8.11E-03
Grm2	5.89E+01	2.91E+00	4.52E+01	1.07E+01	0.38	2.64E-01	4.18E+01	4.90E+00	4.52E+01	1.07E+01	-0.11	7.82E-01	3.65E+01	6.13E+00	5.89E+01	2.91E+00	-0.69	1.29E-02
Grm3	7.93E+02	3.29E+01	7.54E+02	4.55E+01	0.07	5.10E-01	7.51E+02	1.62E+01	7.54E+02	4.55E+01	-0.01	9.44E-01	7.52E+02	7.57E+01	7.93E+02	3.29E+01	-0.08	6.36E-01
Grm4	7.07E+02	2.81E+01	5.43E+02	1.34E+02	0.38	2.82E-01	4.59E+02	3.81E+01	5.43E+02	1.34E+02	-0.24	5.68E-01	3.98E+02	7.16E+01	7.07E+02	2.81E+01	-0.83	5.98E-03
Grm5	2.64E+03	1.23E+02	2.75E+03	2.77E+02	-0.06	7.26E-01	2.53E+03	1.38E+02	2.75E+03	2.77E+02	-0.12	4.98E-01	2.43E+03	2.18E+02	2.64E+03	1.23E+02	-0.12	4.11E-01
Grm7	8.04E+02	4.01E+01	7.81E+02	9.70E+01	0.04	8.33E-01	7.40E+02	3.59E+01	7.81E+02	9.70E+01	-0.08	7.06E-01	7.11E+02	6.03E+01	8.04E+02	4.01E+01	-0.18	2.33E-01
Grm8	1.80E+02	7.54E+00	1.60E+02	1.75E+01	0.17	3.20E-01	1.55E+02	9.84E+00	1.60E+02	1.75E+01	-0.04	8.26E-01	1.49E+02	1.30E+01	1.80E+02	7.54E+00	-0.27	7.19E-02
Hmgcs2	3.62E+0	4.88E+0	2.28E+0	3.47E+0	0.67	5.21E-	3.30E+0	6.73E+0	2.28E+0	3.47E+0	0.53	2.19E-	3.36E+0	1.65E+0	3.62E+0	4.88E+0	-0.11	6.33E-

	1	0	1	0		02	1	0	1	0		01	1	0	1	0		01
Kcna2	1.12E+04	3.21E+02	8.38E+03	1.21E+03	0.42	6.80E-02	8.40E+03	4.46E+02	8.38E+03	1.21E+03	0.00	9.89E-01	7.75E+03	9.34E+02	1.12E+04	3.21E+02	-0.53	1.25E-02
Kcna3	1.17E+02	5.88E+00	9.30E+01	8.94E+00	0.33	5.04E-02	1.05E+02	8.71E+00	9.30E+01	8.94E+00	0.18	3.49E-01	9.94E+01	7.22E+00	1.17E+02	5.88E+00	-0.24	8.50E-02
Kcna6	9.42E+02	2.44E+01	9.68E+02	1.51E+02	-0.04	8.75E-01	7.84E+02	5.07E+01	9.68E+02	1.51E+02	-0.30	2.94E-01	7.91E+02	1.01E+02	9.42E+02	2.44E+01	-0.25	2.00E-01
Kcnab1	2.90E+03	1.11E+02	2.48E+03	2.20E+02	0.23	1.30E-01	2.73E+03	1.39E+02	2.48E+03	2.20E+02	0.14	3.71E-01	2.52E+03	1.88E+02	2.90E+03	1.11E+02	-0.21	1.14E-01
Kcnab2	7.55E+03	2.10E+02	5.80E+03	1.28E+03	0.38	2.32E-01	5.30E+03	3.80E+02	5.80E+03	1.28E+03	-0.13	7.21E-01	4.66E+03	7.95E+02	7.55E+03	2.10E+02	-0.70	1.37E-02
Kcnab3	1.91E+03	2.22E+01	1.12E+03	2.90E+02	0.77	4.09E-02	1.12E+03	6.19E+01	1.12E+03	2.90E+02	0.00	9.92E-01	1.04E+03	1.25E+02	1.91E+03	2.22E+01	-0.88	7.56E-04
Kcnb2	6.04E+02	2.76E+01	6.21E+02	4.17E+01	-0.04	7.42E-01	6.31E+02	4.41E+01	6.21E+02	4.17E+01	0.02	8.78E-01	6.59E+02	3.83E+01	6.04E+02	2.76E+01	0.13	2.71E-01
Kcnc2	1.83E+03	7.89E+01	1.79E+03	1.85E+02	0.03	8.51E-01	1.62E+03	7.28E+01	1.79E+03	1.85E+02	-0.14	4.44E-01	1.59E+03	1.27E+02	1.83E+03	7.89E+01	-0.20	1.56E-01
Kcnc3	8.44E+03	2.40E+02	5.31E+03	1.41E+03	0.67	7.65E-02	4.64E+03	3.11E+02	5.31E+03	1.41E+03	-0.19	6.62E-01	4.09E+03	8.11E+02	8.44E+03	2.40E+02	-1.05	2.27E-03
Kcnc4	1.32E+03	3.24E+01	1.05E+03	2.26E+02	0.33	2.84E-01	9.32E+02	6.92E+01	1.05E+03	2.26E+02	-0.17	6.37E-01	8.53E+02	1.31E+02	1.32E+03	3.24E+01	-0.63	1.47E-02
Kcnd2	1.51E+03	3.07E+01	1.28E+03	1.54E+02	0.25	1.84E-01	1.23E+03	5.06E+01	1.28E+03	1.54E+02	-0.06	7.75E-01	1.17E+03	1.09E+02	1.51E+03	3.07E+01	-0.38	2.30E-02
Kcne1l	3.41E+0	2.98E+0	2.63E+0	1.57E+0	0.38	4.89E-	3.68E+0	2.81E+0	2.63E+0	1.57E+0	0.49	1.13E-	3.85E+0	2.76E+0	3.41E+0	2.98E+0	0.17	3.06E-

	2	1	2	1		02	2	1	2	1		02	2	1	2	1		01
Kcne2	4.11E+00	8.33E-01	2.83E+00	7.49E-01	0.54	2.83E-01	3.38E+00	1.18E+00	2.83E+00	7.49E-01	0.25	7.06E-01	3.27E+00	6.01E-01	4.11E+00	8.33E-01	-0.33	4.37E-01
Kcng1	9.30E+01	5.14E+00	6.63E+01	1.81E+01	0.49	2.08E-01	5.49E+01	4.99E+00	6.63E+01	1.81E+01	-0.27	5.67E-01	5.44E+01	8.28E+00	9.30E+01	5.14E+00	-0.77	3.81E-03
Kcng4	1.72E+03	5.39E+01	1.11E+03	2.05E+02	0.64	2.85E-02	9.68E+02	9.12E+01	1.11E+03	2.05E+02	-0.20	5.51E-01	1.02E+03	1.69E+02	1.72E+03	5.39E+01	-0.76	7.32E-03
Kcnh2	2.26E+03	8.74E+01	1.83E+03	3.67E+02	0.31	2.99E-01	1.63E+03	1.52E+02	1.83E+03	3.67E+02	-0.16	6.39E-01	1.53E+03	2.52E+02	2.26E+03	8.74E+01	-0.57	3.22E-02
Kcnh4	1.00E+01	8.33E-01	1.13E+01	1.65E+00	-0.17	5.07E-01	6.88E+00	1.63E+00	1.13E+01	1.65E+00	-0.72	8.30E-02	1.01E+01	2.22E+00	1.00E+01	8.33E-01	0.01	9.68E-01
Kcnh7	9.35E+02	9.25E+01	1.02E+03	5.63E+01	-0.12	4.73E-01	1.00E+03	4.09E+01	1.02E+03	5.63E+01	-0.02	8.24E-01	1.05E+03	6.33E+01	9.35E+02	9.25E+01	0.17	3.26E-01
Kcnip1	1.47E+03	9.60E+01	1.37E+03	6.71E+01	0.11	3.84E-01	1.37E+03	5.75E+01	1.37E+03	6.71E+01	0.01	9.30E-01	1.30E+03	7.45E+01	1.47E+03	9.60E+01	-0.18	1.91E-01
Kcnip3	5.67E+02	2.60E+01	6.15E+02	9.27E+01	-0.11	6.43E-01	4.66E+02	4.73E+01	6.15E+02	9.27E+01	-0.40	1.95E-01	5.66E+02	7.14E+01	5.67E+02	2.60E+01	0.00	9.80E-01
Kcnip4	1.23E+03	8.28E+01	1.10E+03	5.99E+01	0.17	2.19E-01	1.05E+03	7.06E+01	1.10E+03	5.99E+01	-0.06	6.16E-01	1.08E+03	6.81E+01	1.23E+03	8.28E+01	-0.19	1.97E-01
Kcnj10	1.86E+04	3.43E+02	1.55E+04	2.51E+03	0.26	2.81E-01	1.47E+04	1.05E+03	1.55E+04	2.51E+03	-0.08	7.65E-01	1.35E+04	1.79E+03	1.86E+04	3.43E+02	-0.46	3.52E-02
Kcnj11	4.61E+02	1.49E+01	3.76E+02	8.59E+01	0.29	3.74E-01	3.02E+02	2.91E+01	3.76E+02	8.59E+01	-0.31	4.47E-01	2.96E+02	3.83E+01	4.61E+02	1.49E+01	-0.64	6.13E-03
Kcnj12	2.22E+0	4.30E+0	1.51E+0	3.29E+0	0.56	8.04E-	1.48E+0	1.46E+0	1.51E+0	3.29E+0	-0.02	9.46E-	1.28E+0	1.92E+0	2.22E+0	4.30E+0	-0.80	3.75E-

	2	0	2	1		02	2	1	2	1		01	2	1	2	0		03
Kcnj13	2.36E+00	5.77E-01	1.33E+00	6.67E-01	0.82	2.71E-01	1.08E+00	3.33E-01	1.33E+00	6.67E-01	-0.31	7.41E-01	2.31E+00	1.15E+00	2.36E+00	5.77E-01	-0.03	9.71E-01
Kcnj14	4.41E+02	1.61E+01	2.94E+02	3.18E+01	0.59	3.88E-03	2.43E+02	1.44E+01	2.94E+02	3.18E+01	-0.28	1.88E-01	2.77E+02	4.61E+01	4.41E+02	1.61E+01	-0.67	1.46E-02
Kcnj2	3.96E+02	2.33E+01	3.74E+02	1.35E+01	0.09	4.22E-01	3.52E+02	2.06E+01	3.74E+02	1.35E+01	-0.09	3.95E-01	3.61E+02	2.65E+01	3.96E+02	2.33E+01	-0.13	3.41E-01
Kcnj3	1.52E+03	5.99E+01	1.45E+03	1.31E+02	0.07	6.53E-01	1.35E+03	6.52E+01	1.45E+03	1.31E+02	-0.10	5.42E-01	1.33E+03	1.23E+02	1.52E+03	5.99E+01	-0.19	2.20E-01
Kcnj4	4.11E+00	4.77E-01	2.83E+00	1.19E+00	0.54	3.58E-01	2.86E+00	1.38E+00	2.83E+00	1.19E+00	0.02	9.87E-01	3.82E+00	7.03E-01	4.11E+00	4.77E-01	-0.10	7.45E-01
Kcnk1	1.98E+03	6.28E+01	1.94E+03	1.49E+02	0.03	8.15E-01	1.90E+03	7.91E+01	1.94E+03	1.49E+02	-0.03	8.33E-01	1.91E+03	1.96E+02	1.98E+03	6.28E+01	-0.05	7.57E-01
Kcnk12	4.48E+02	1.08E+01	2.63E+02	6.32E+01	0.77	3.27E-02	2.12E+02	1.77E+01	2.63E+02	6.32E+01	-0.31	4.68E-01	2.11E+02	3.62E+01	4.48E+02	1.08E+01	-1.09	8.19E-04
Kcnk13	4.55E+02	1.70E+01	4.57E+02	5.11E+01	-0.01	9.68E-01	4.04E+02	1.63E+01	4.57E+02	5.11E+01	-0.18	3.58E-01	4.02E+02	4.71E+01	4.55E+02	1.70E+01	-0.18	3.33E-01
Kcnk7	1.83E+01	2.29E+00	1.12E+01	1.19E+00	0.72	2.54E-02	9.83E+00	2.73E+00	1.12E+01	1.19E+00	-0.18	6.68E-01	1.92E+01	2.75E+00	1.83E+01	2.29E+00	0.06	8.20E-01
Kcnk9	1.77E+03	6.71E+01	2.42E+03	4.00E+02	-0.45	1.69E-01	1.88E+03	1.52E+02	2.42E+03	4.00E+02	-0.37	2.47E-01	2.18E+03	2.83E+02	1.77E+03	6.71E+01	0.30	2.14E-01
Kcnma1	2.43E+03	6.66E+01	2.19E+03	2.12E+02	0.15	3.27E-01	2.15E+03	1.30E+02	2.19E+03	2.12E+02	-0.03	8.82E-01	2.09E+03	1.72E+02	2.43E+03	6.66E+01	-0.22	1.13E-01
Kcnn2	5.05E+0	1.02E+0	5.24E+0	4.84E+0	-0.05	7.19E-	4.22E+0	4.11E+0	5.24E+0	4.84E+0	-0.31	1.40E-	4.76E+0	5.82E+0	5.05E+0	1.02E+0	-0.09	6.44E-

	2	1	2	1		01	2	1	2	1		01	2	1	2	1		01
Kcnn3	5.30E+02	8.57E+00	4.88E+02	5.49E+01	0.12	4.88E-01	4.11E+02	3.09E+01	4.88E+02	5.49E+01	-0.25	2.56E-01	4.37E+02	3.72E+01	5.30E+02	8.57E+00	-0.28	5.54E-02
Kcnn4	1.13E+01	8.72E-01	6.50E+00	1.15E+00	0.79	8.62E-03	6.16E+00	2.39E+00	6.50E+00	1.15E+00	-0.08	9.01E-01	1.22E+01	2.35E+00	1.13E+01	8.72E-01	0.11	7.24E-01
Kcnq1	5.84E+01	4.82E+00	4.35E+01	3.97E+00	0.43	3.90E-02	4.18E+01	4.88E+00	4.35E+01	3.97E+00	-0.06	7.94E-01	4.27E+01	6.29E+00	5.84E+01	4.82E+00	-0.45	7.74E-02
Kcnq2	4.64E+03	1.66E+02	4.47E+03	7.57E+02	0.05	8.34E-01	3.62E+03	3.26E+02	4.47E+03	7.57E+02	-0.30	3.40E-01	3.68E+03	4.90E+02	4.64E+03	1.66E+02	-0.33	1.14E-01
Kcnq3	1.85E+03	2.59E+01	1.80E+03	2.77E+02	0.03	8.82E-01	1.55E+03	1.32E+02	1.80E+03	2.77E+02	-0.22	4.42E-01	1.47E+03	1.61E+02	1.85E+03	2.59E+01	-0.33	6.57E-02
Kcnq4	1.98E+02	9.50E+00	1.99E+02	3.56E+01	0.00	9.97E-01	1.61E+02	1.65E+01	1.99E+02	3.56E+01	-0.30	3.74E-01	1.49E+02	1.94E+01	1.98E+02	9.50E+00	-0.41	5.61E-02
Kcns1	7.69E+01	2.27E+00	6.82E+01	1.15E+01	0.17	4.87E-01	6.78E+01	6.08E+00	6.82E+01	1.15E+01	-0.01	9.79E-01	6.42E+01	7.68E+00	7.69E+01	2.27E+00	-0.26	1.66E-01
Kctd10	4.18E+02	1.25E+01	3.44E+02	4.32E+01	0.28	1.53E-01	3.08E+02	2.23E+01	3.44E+02	4.32E+01	-0.16	4.88E-01	3.54E+02	4.32E+01	4.18E+02	1.25E+01	-0.24	2.09E-01
Kctd12b	2.74E+02	1.42E+01	2.43E+02	1.25E+01	0.18	1.26E-01	2.35E+02	1.91E+01	2.43E+02	1.25E+01	-0.05	7.38E-01	2.47E+02	1.92E+01	2.74E+02	1.42E+01	-0.15	2.83E-01
Kctd13	8.61E+02	2.98E+01	8.45E+02	1.20E+02	0.03	9.06E-01	7.64E+02	2.85E+01	8.45E+02	1.20E+02	-0.15	5.39E-01	7.72E+02	8.73E+01	8.61E+02	2.98E+01	-0.16	3.74E-01
Kctd15	3.22E+02	7.56E+00	2.97E+02	3.32E+01	0.12	4.90E-01	2.49E+02	1.59E+01	2.97E+02	3.32E+01	-0.25	2.35E-01	2.53E+02	4.10E+01	3.22E+02	7.56E+00	-0.35	1.57E-01
Kctd16	1.41E+0	8.82E+0	1.39E+0	8.98E+0	0.02	8.75E-	1.35E+0	3.84E+0	1.39E+0	8.98E+0	-0.04	6.85E-	1.58E+0	1.14E+0	1.41E+0	8.82E+0	0.17	2.60E-

	2	0	2	0		01	2	0	2	0		01	2	1	2	0		01
Kctd17	1.34E+03	2.72E+01	1.51E+03	1.85E+02	-0.17	4.01E-01	1.38E+03	7.73E+01	1.51E+03	1.85E+02	-0.14	5.20E-01	1.46E+03	1.75E+02	1.34E+03	2.72E+01	0.12	5.45E-01
Kctd18	5.28E+02	2.46E+01	4.63E+02	3.40E+01	0.19	1.53E-01	5.03E+02	2.90E+01	4.63E+02	3.40E+01	0.12	3.96E-01	4.99E+02	2.44E+01	5.28E+02	2.46E+01	-0.08	4.23E-01
Kctd2	9.92E+02	3.53E+01	8.80E+02	9.07E+01	0.17	2.90E-01	8.80E+02	5.79E+01	8.80E+02	9.07E+01	0.00	9.96E-01	8.61E+02	1.28E+02	9.92E+02	3.53E+01	-0.20	3.66E-01
Kctd20	1.24E+03	2.13E+01	1.15E+03	1.25E+02	0.11	4.85E-01	1.13E+03	5.56E+01	1.15E+03	1.25E+02	-0.02	9.06E-01	1.07E+03	1.05E+02	1.24E+03	2.13E+01	-0.21	1.66E-01
Kctd3	1.89E+03	3.34E+01	1.81E+03	2.58E+02	0.07	7.53E-01	1.63E+03	1.02E+02	1.81E+03	2.58E+02	-0.15	5.35E-01	1.58E+03	2.16E+02	1.89E+03	3.34E+01	-0.26	2.06E-01
Kctd4	9.62E+02	8.37E+01	8.06E+02	4.87E+01	0.26	1.45E-01	7.82E+02	8.45E+01	8.06E+02	4.87E+01	-0.04	8.16E-01	8.17E+02	6.13E+01	9.62E+02	8.37E+01	-0.24	1.95E-01
Kctd5	6.11E+02	1.98E+01	5.96E+02	3.09E+01	0.03	7.06E-01	6.54E+02	2.96E+01	5.96E+02	3.09E+01	0.13	2.11E-01	6.83E+02	4.53E+01	6.11E+02	1.98E+01	0.16	1.89E-01
Kif11	3.96E+01	4.66E+00	3.35E+01	2.62E+00	0.24	2.89E-01	3.69E+01	3.89E+00	3.35E+01	2.62E+00	0.14	4.90E-01	3.48E+01	3.48E+00	3.96E+01	4.66E+00	-0.19	4.32E-01
Kif13a	8.64E+02	2.54E+01	8.45E+02	1.31E+02	0.03	8.96E-01	7.39E+02	7.11E+01	8.45E+02	1.31E+02	-0.19	4.97E-01	7.05E+02	9.43E+01	8.64E+02	2.54E+01	-0.29	1.59E-01
Kif14	9.50E+00	1.39E+00	8.33E+00	2.09E+00	0.19	6.55E-01	5.18E+00	6.01E-01	8.33E+00	2.09E+00	-0.69	1.99E-01	6.39E+00	9.10E-01	9.50E+00	1.39E+00	-0.57	9.57E-02
Kif15	5.73E+01	3.83E+00	4.92E+01	4.06E+00	0.22	1.74E-01	6.06E+01	8.95E+00	4.92E+01	4.06E+00	0.30	2.81E-01	5.05E+01	4.09E+00	5.73E+01	3.83E+00	-0.18	2.47E-01
Kif1a	1.84E+0	5.67E+0	1.56E+0	2.79E+0	0.24	3.64E-	1.44E+0	1.19E+0	1.56E+0	2.79E+0	-0.11	7.18E-	1.31E+0	2.03E+0	1.84E+0	5.67E+0	-0.49	4.80E-

	4	2	4	3		01	4	3	4	3		01	4	3	4	2		02
Kif1b	2.30E+04	4.73E+02	1.89E+04	2.93E+03	0.28	2.22E-01	1.86E+04	1.15E+03	1.89E+04	2.93E+03	-0.02	9.23E-01	1.63E+04	2.14E+03	2.30E+04	4.73E+02	-0.50	2.49E-02
Kif1c	2.89E+03	1.32E+02	2.44E+03	4.10E+02	0.24	3.37E-01	2.60E+03	2.18E+02	2.44E+03	4.10E+02	0.09	7.46E-01	2.17E+03	3.79E+02	2.89E+03	1.32E+02	-0.42	1.20E-01
Kif20b	3.66E+01	3.48E+00	3.28E+01	3.93E+00	0.16	4.90E-01	3.30E+01	2.42E+00	3.28E+01	3.93E+00	0.01	9.79E-01	3.24E+01	2.00E+00	3.66E+01	3.48E+00	-0.17	3.30E-01
Kif21a	4.60E+03	2.17E+02	5.54E+03	6.22E+02	-0.27	2.03E-01	4.45E+03	2.92E+02	5.54E+03	6.22E+02	-0.32	1.57E-01	4.98E+03	5.47E+02	4.60E+03	2.17E+02	0.12	5.35E-01
Kif21b	1.29E+03	5.28E+01	1.16E+03	1.81E+02	0.16	5.08E-01	1.11E+03	1.03E+02	1.16E+03	1.81E+02	-0.07	8.04E-01	1.10E+03	1.40E+02	1.29E+03	5.28E+01	-0.24	2.32E-01
Kif23	1.54E+01	2.43E+00	1.03E+01	1.73E+00	0.57	1.25E-01	1.14E+01	9.80E-01	1.03E+01	1.73E+00	0.15	5.92E-01	1.12E+01	2.23E+00	1.54E+01	2.43E+00	-0.45	2.38E-01
Kif26a	4.78E+01	4.72E+00	4.87E+01	5.53E+00	-0.03	9.03E-01	4.63E+01	6.83E+00	4.87E+01	5.53E+00	-0.07	7.95E-01	4.21E+01	7.22E+00	4.78E+01	4.72E+00	-0.18	5.30E-01
Kif26b	6.50E+02	2.58E+01	5.37E+02	1.11E+02	0.27	3.65E-01	5.49E+02	3.96E+01	5.37E+02	1.11E+02	0.03	9.22E-01	4.53E+02	6.72E+01	6.50E+02	2.58E+01	-0.52	3.17E-02
Kif2a	1.34E+03	6.84E+01	1.37E+03	1.21E+02	-0.03	8.45E-01	1.32E+03	6.96E+01	1.37E+03	1.21E+02	-0.05	7.34E-01	1.33E+03	1.03E+02	1.34E+03	6.84E+01	-0.01	9.55E-01
Kif2c	7.25E+00	1.20E+00	4.33E+00	1.05E+00	0.74	9.85E-02	5.74E+00	1.49E+00	4.33E+00	1.05E+00	0.41	4.61E-01	5.65E+00	8.47E-01	7.25E+00	1.20E+00	-0.36	3.06E-01
Kif3a	2.72E+03	1.40E+02	2.78E+03	1.82E+02	-0.03	7.88E-01	2.46E+03	9.36E+01	2.78E+03	1.82E+02	-0.18	1.54E-01	2.61E+03	2.29E+02	2.72E+03	1.40E+02	-0.06	6.99E-01
Kif3c	2.32E+0	6.07E+0	1.90E+0	3.78E+0	0.29	3.14E-	1.66E+0	1.30E+0	1.90E+0	3.78E+0	-0.19	5.70E-	1.51E+0	2.29E+0	2.32E+0	6.07E+0	-0.62	1.51E-

	3	1	3	2		01	3	2	3	2		01	3	2	3	1		02
Kif5a	1.28E+04	4.65E+02	1.85E+04	3.03E+03	-0.53	1.21E-01	1.38E+04	1.30E+03	1.85E+04	3.03E+03	-0.42	2.03E-01	1.51E+04	2.30E+03	1.28E+04	4.65E+02	0.24	3.59E-01
Kif5b	1.36E+04	8.91E+02	1.42E+04	6.97E+02	-0.07	5.82E-01	1.37E+04	4.53E+02	1.42E+04	6.97E+02	-0.06	5.33E-01	1.42E+04	1.06E+03	1.36E+04	8.91E+02	0.06	6.64E-01
Kif5c	9.22E+03	2.46E+02	1.24E+04	1.46E+03	-0.42	8.55E-02	1.03E+04	6.92E+02	1.24E+04	1.46E+03	-0.26	2.47E-01	1.12E+04	1.25E+03	9.22E+03	2.46E+02	0.28	1.72E-01
Kif6	6.04E+01	3.46E+00	5.37E+01	7.07E+00	0.17	4.22E-01	5.97E+01	4.57E+00	5.37E+01	7.07E+00	0.15	4.92E-01	6.19E+01	9.65E+00	6.04E+01	3.46E+00	0.04	8.84E-01
Kif9	1.45E+02	1.46E+01	1.35E+02	1.25E+01	0.11	6.00E-01	1.15E+02	1.15E+01	1.35E+02	1.25E+01	-0.23	2.67E-01	1.17E+02	1.79E+01	1.45E+02	1.46E+01	-0.31	2.55E-01
Kifap3	4.65E+03	2.44E+02	5.20E+03	4.81E+02	-0.16	3.35E-01	4.93E+03	2.26E+02	5.20E+03	4.81E+02	-0.08	6.29E-01	5.00E+03	3.96E+02	4.65E+03	2.44E+02	0.10	4.74E-01
Kifc2	1.79E+03	8.19E+01	1.93E+03	3.29E+02	-0.11	6.97E-01	1.62E+03	1.51E+02	1.93E+03	3.29E+02	-0.25	4.26E-01	1.66E+03	2.61E+02	1.79E+03	8.19E+01	-0.11	6.62E-01
Kifc3	5.24E+02	3.33E+01	6.90E+02	1.08E+02	-0.39	1.96E-01	5.23E+02	5.66E+01	6.90E+02	1.08E+02	-0.40	2.12E-01	5.92E+02	1.01E+02	5.24E+02	3.33E+01	0.17	5.47E-01
Myo10	3.96E+02	2.16E+01	5.42E+02	6.05E+01	-0.45	6.11E-02	5.47E+02	4.95E+01	5.42E+02	6.05E+01	0.01	9.53E-01	5.44E+02	7.12E+01	3.96E+02	2.16E+01	0.46	9.30E-02
Myo15	1.50E+01	2.62E+00	1.35E+01	2.92E+00	0.15	7.13E-01	1.58E+01	3.67E+00	1.35E+01	2.92E+00	0.23	6.34E-01	1.28E+01	2.57E+00	1.50E+01	2.62E+00	-0.23	5.57E-01
Myo18a	2.58E+03	9.80E+01	2.24E+03	4.49E+02	0.20	4.91E-01	1.83E+03	1.52E+02	2.24E+03	4.49E+02	-0.29	4.17E-01	1.80E+03	3.06E+02	2.58E+03	9.80E+01	-0.52	5.06E-02
Myo1a	2.26E+0	2.65E+0	1.80E+0	3.88E+0	0.33	3.52E-	1.96E+0	1.26E+0	1.80E+0	3.88E+0	0.12	7.07E-	2.19E+0	3.09E+0	2.26E+0	2.65E+0	-0.05	8.57E-

	1	0	1	0		01	1	0	1	0		01	1	0	1	0		01
Myo1b	2.84E+02	2.07E+01	2.78E+02	1.97E+01	0.03	8.44E-01	2.74E+02	8.70E+00	2.78E+02	1.97E+01	-0.02	8.36E-01	2.58E+02	1.35E+01	2.84E+02	2.07E+01	-0.14	3.19E-01
Myo1c	1.94E+02	9.77E+00	2.31E+02	3.23E+01	-0.25	3.13E-01	1.77E+02	1.65E+01	2.31E+02	3.23E+01	-0.38	1.78E-01	1.63E+02	2.86E+01	1.94E+02	9.77E+00	-0.25	3.41E-01
Myo1d	7.16E+02	1.53E+01	6.06E+02	1.13E+02	0.24	3.76E-01	5.23E+02	5.06E+01	6.06E+02	1.13E+02	-0.21	5.28E-01	4.76E+02	8.76E+01	7.16E+02	1.53E+01	-0.59	4.09E-02
Myo1e	3.69E+02	9.95E+00	3.78E+02	5.68E+01	-0.03	8.82E-01	2.65E+02	3.17E+01	3.78E+02	5.68E+01	-0.51	1.21E-01	2.84E+02	4.03E+01	3.69E+02	9.95E+00	-0.38	8.93E-02
Myo1g	1.10E+01	1.80E+00	1.05E+01	1.93E+00	0.07	8.54E-01	1.11E+01	2.65E+00	1.05E+01	1.93E+00	0.09	8.48E-01	1.36E+01	1.90E+00	1.10E+01	1.80E+00	0.31	3.45E-01
Myo5c	1.23E+01	1.98E+00	1.05E+01	2.73E+00	0.23	6.11E-01	1.25E+01	1.92E+00	1.05E+01	2.73E+00	0.25	5.59E-01	1.16E+01	2.65E+00	1.23E+01	1.98E+00	-0.08	8.45E-01
Myo6	5.38E+03	1.91E+02	5.23E+03	3.74E+02	0.04	7.16E-01	5.21E+03	2.57E+02	5.23E+03	3.74E+02	0.00	9.73E-01	5.32E+03	4.29E+02	5.38E+03	1.91E+02	-0.02	8.95E-01
Myo7a	4.47E+01	5.35E+00	5.13E+01	1.12E+01	-0.20	6.10E-01	3.38E+01	4.31E+00	5.13E+01	1.12E+01	-0.60	1.91E-01	4.05E+01	6.73E+00	4.47E+01	5.35E+00	-0.14	6.34E-01
Myo9a	4.23E+03	2.63E+02	4.20E+03	1.83E+02	0.01	9.13E-01	4.07E+03	1.57E+02	4.20E+03	1.83E+02	-0.04	6.24E-01	4.24E+03	2.61E+02	4.23E+03	2.63E+02	0.00	9.81E-01
Pemt	1.07E+02	7.86E+00	7.80E+01	5.26E+00	0.46	1.37E-02	7.97E+01	1.08E+01	7.80E+01	5.26E+00	0.03	8.92E-01	1.04E+02	6.84E+00	1.07E+02	7.86E+00	-0.05	7.47E-01
Scn1a	1.25E+04	5.85E+02	1.06E+04	6.92E+02	0.23	7.48E-02	1.14E+04	3.58E+02	1.06E+04	6.92E+02	0.10	3.35E-01	1.11E+04	8.64E+02	1.25E+04	5.85E+02	-0.17	2.19E-01
Scn1b	4.66E+0	1.53E+0	3.66E+0	7.98E+0	0.35	2.68E-	2.87E+0	2.40E+0	3.66E+0	7.98E+0	-0.35	3.79E-	3.09E+0	4.78E+0	4.66E+0	1.53E+0	-0.59	2.00E-

	3	2	3	2		01	3	2	3	2		01	3	2	3	2		02
Scn2a	2.91E+03	1.77E+02	2.73E+03	1.93E+02	0.09	4.96E-01	2.72E+03	1.06E+02	2.73E+03	1.93E+02	-0.01	9.65E-01	2.78E+03	2.09E+02	2.91E+03	1.77E+02	-0.06	6.54E-01
Scn2b	2.02E+03	4.07E+01	1.70E+03	3.26E+02	0.26	3.60E-01	1.35E+03	9.26E+01	1.70E+03	3.26E+02	-0.33	3.51E-01	1.34E+03	2.04E+02	2.02E+03	4.07E+01	-0.60	1.93E-02
Scn3a	9.58E+02	7.36E+01	8.94E+02	5.74E+01	0.10	5.10E-01	9.00E+02	3.68E+01	8.94E+02	5.74E+01	0.01	9.26E-01	8.94E+02	5.21E+01	9.58E+02	7.36E+01	-0.10	5.00E-01
Scn3b	5.72E+02	4.20E+01	6.67E+02	6.47E+01	-0.22	2.50E-01	5.54E+02	2.27E+01	6.67E+02	6.47E+01	-0.27	1.48E-01	6.17E+02	5.74E+01	5.72E+02	4.20E+01	0.11	5.43E-01
Scn4a	1.70E+00	9.57E-01	1.33E+00	4.22E-01	0.35	7.39E-01	1.28E+00	7.19E-01	1.33E+00	4.22E-01	-0.06	9.47E-01	1.60E+00	5.63E-01	1.70E+00	9.57E-01	-0.08	9.35E-01
Scn7a	2.47E+02	1.42E+01	2.07E+02	1.27E+01	0.26	5.81E-02	2.27E+02	1.77E+01	2.07E+02	1.27E+01	0.14	3.71E-01	2.24E+02	9.95E+00	2.47E+02	1.42E+01	-0.14	2.21E-01
Scn8a	2.64E+03	9.66E+01	4.19E+03	9.05E+02	-0.67	1.47E-01	2.93E+03	2.59E+02	4.19E+03	9.05E+02	-0.51	2.33E-01	3.53E+03	4.85E+02	2.64E+03	9.66E+01	0.42	1.27E-01
Scn9a	5.26E+02	3.20E+01	4.64E+02	3.20E+01	0.18	1.98E-01	4.99E+02	3.37E+01	4.64E+02	3.20E+01	0.11	4.64E-01	5.42E+02	3.73E+01	5.26E+02	3.20E+01	0.04	7.42E-01
Scnm1	4.97E+02	3.87E+01	3.67E+02	1.39E+01	0.44	1.83E-02	4.85E+02	4.23E+01	3.67E+02	1.39E+01	0.40	3.82E-02	4.51E+02	2.62E+01	4.97E+02	3.87E+01	-0.14	3.45E-01
