# Supplementary material for: Metabolic reprogramming in the spinal cord drives the transition to pain chronicity
Source: bioRxiv. 2025 Feb 1:2025.01.30.635746. Preprint. [Version 1] doi: 10.1101/2025.01.30.635746 (PMC11838349; doi:10.1101/2025.01.30.635746)
Supplement: Supplementary file 1 [file NIHPP2025.01.30.635746V1-supplement-1.pdf]

**Supplemental Fig. S1**

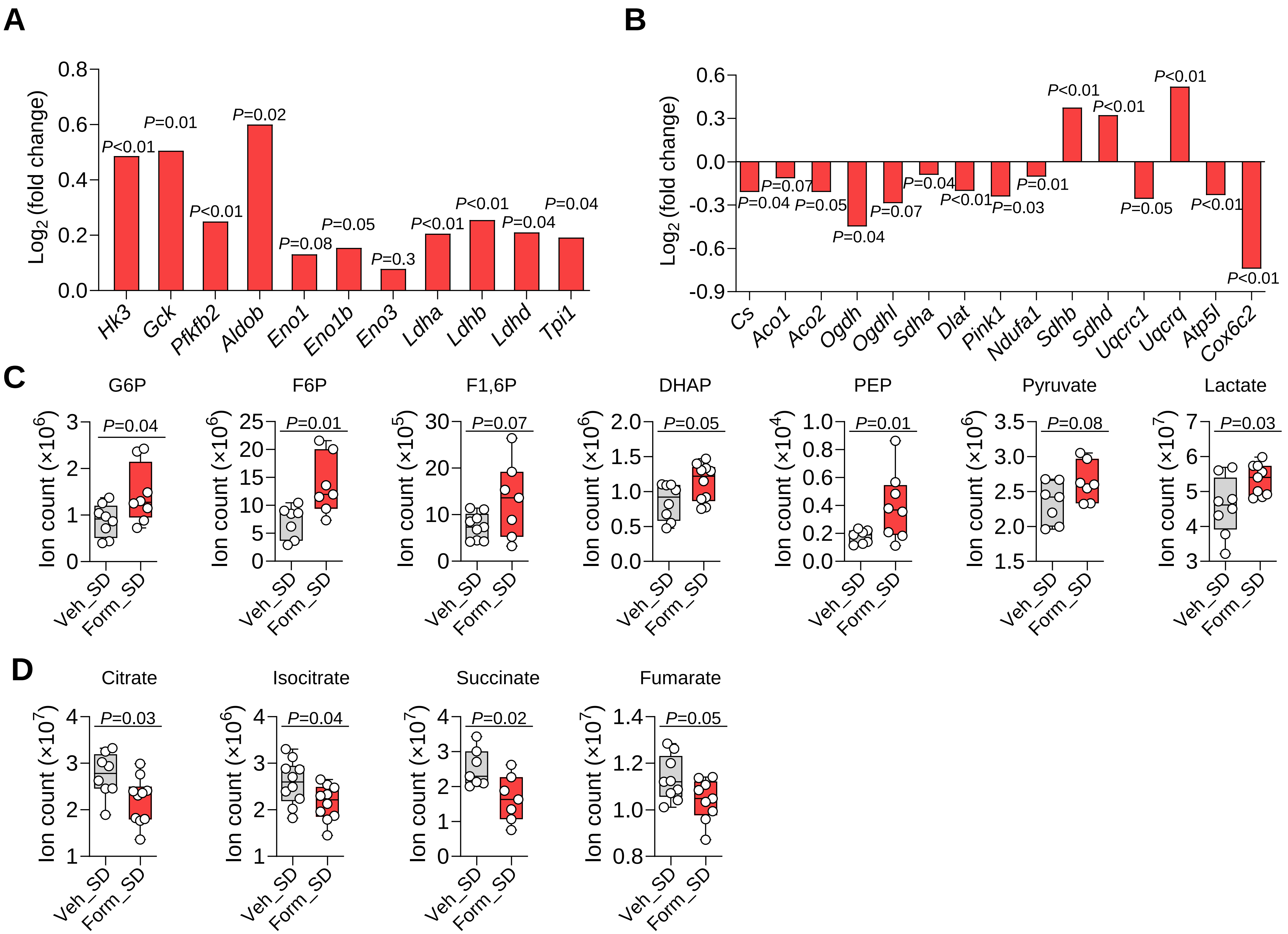

# Supplemental Fig. S2

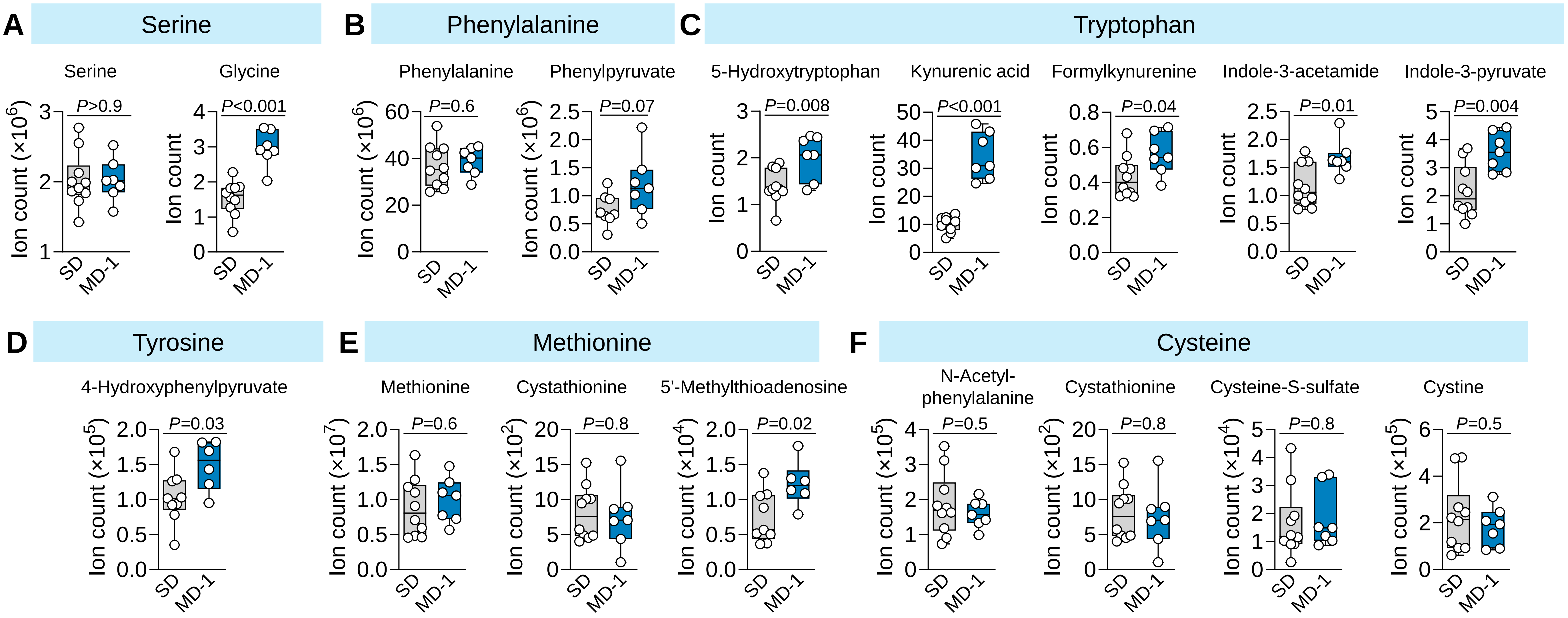

**Supplemental Fig. S3**

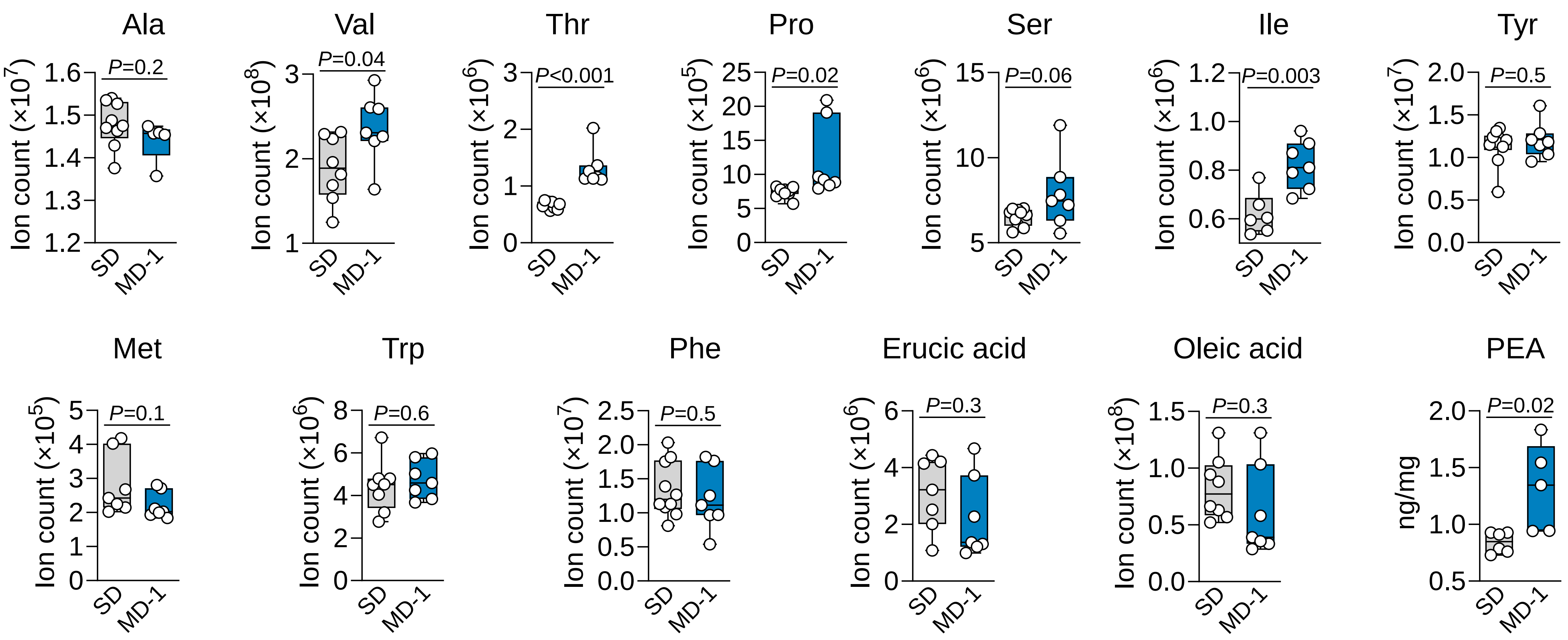

# Supplemental Fig. S4

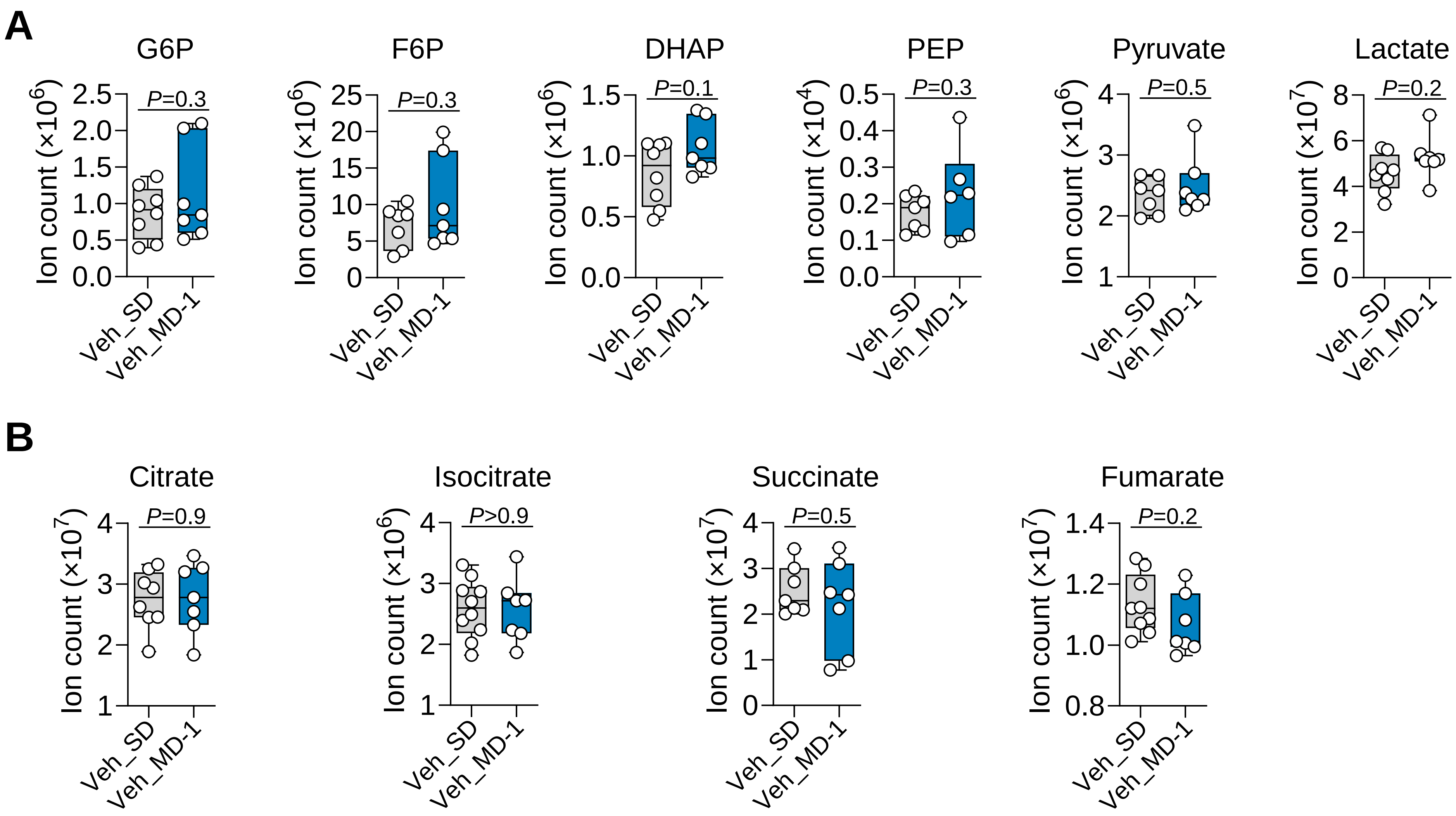

# Supplemental Fig. S5

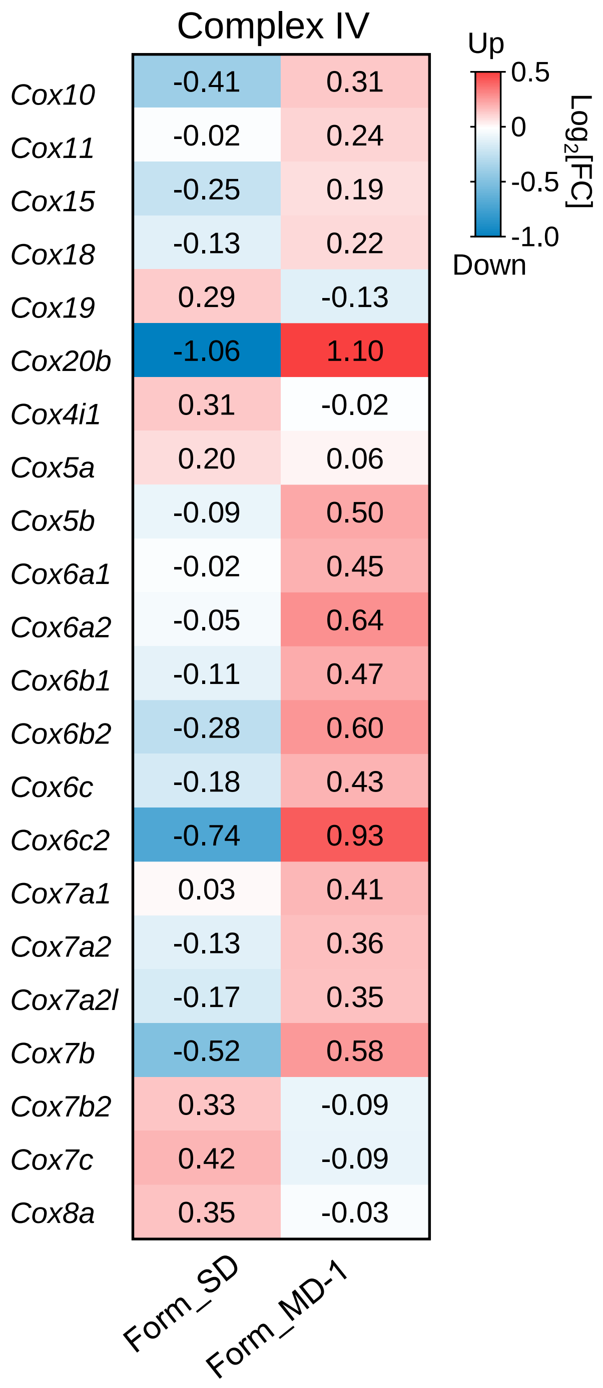

## Supplemental Fig. S6

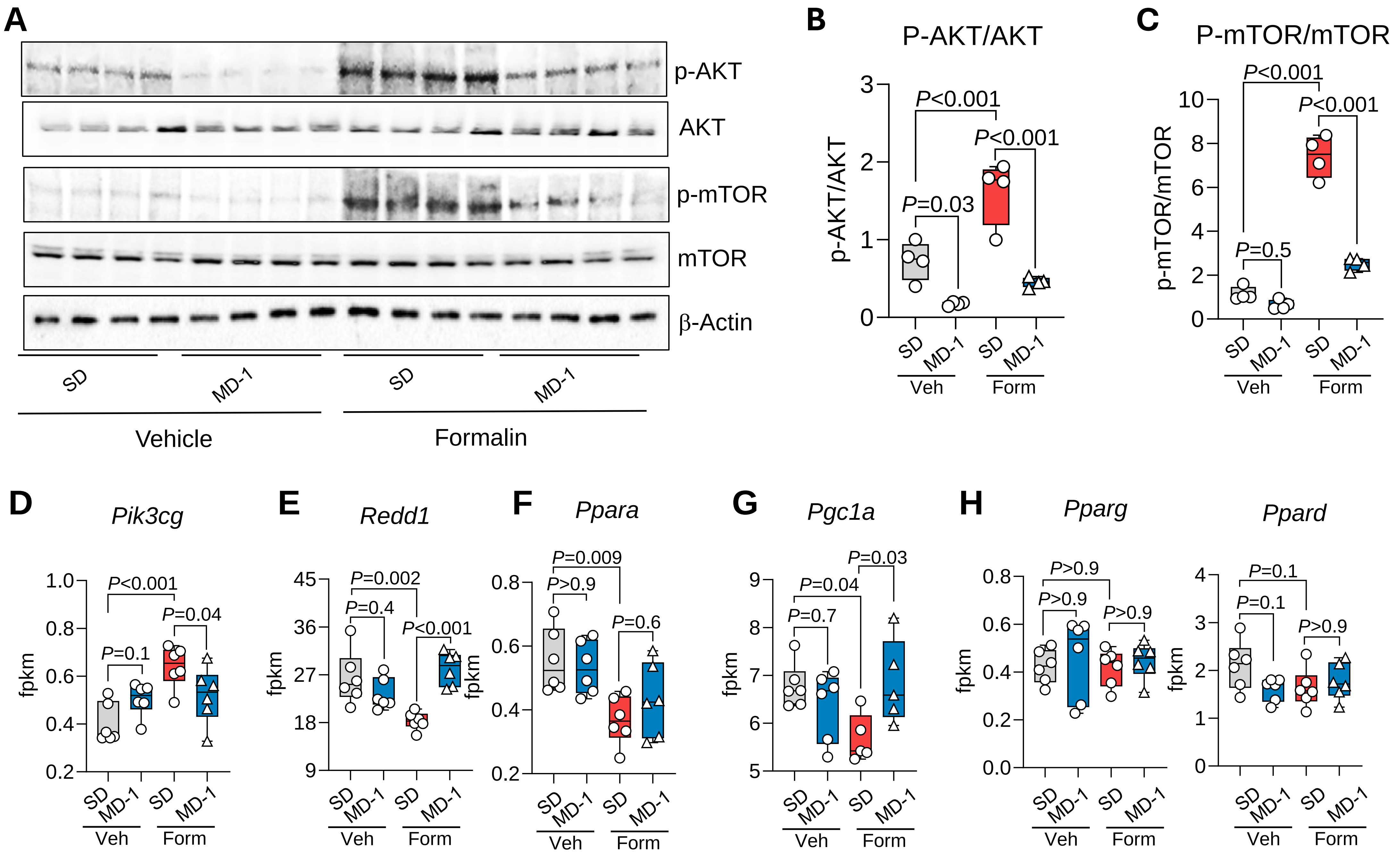

# Supplemental Fig. S7

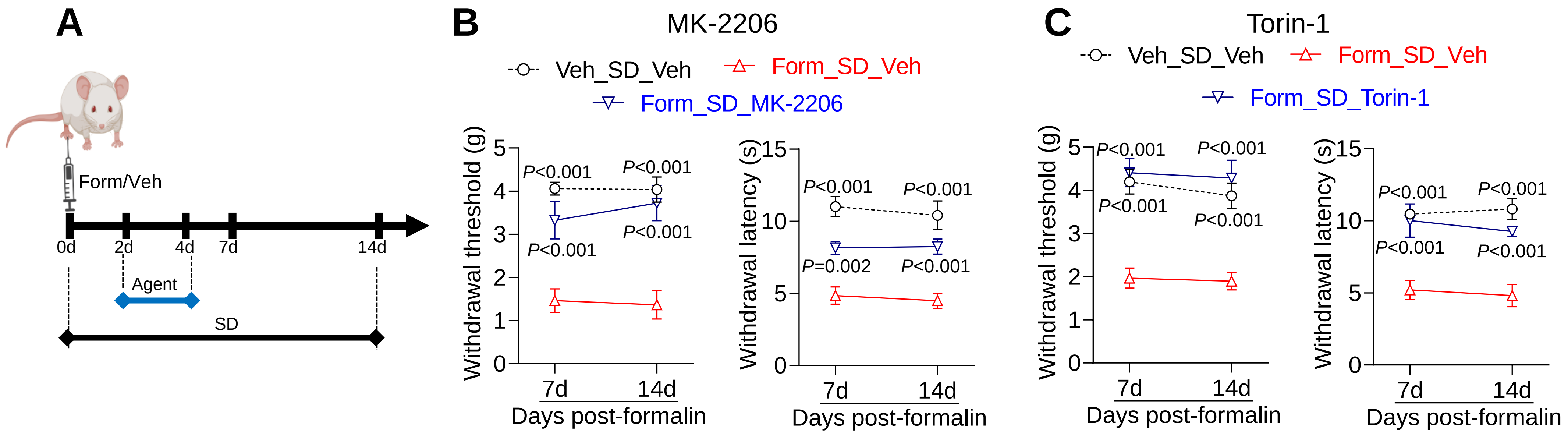

# Supplemental Fig. S8

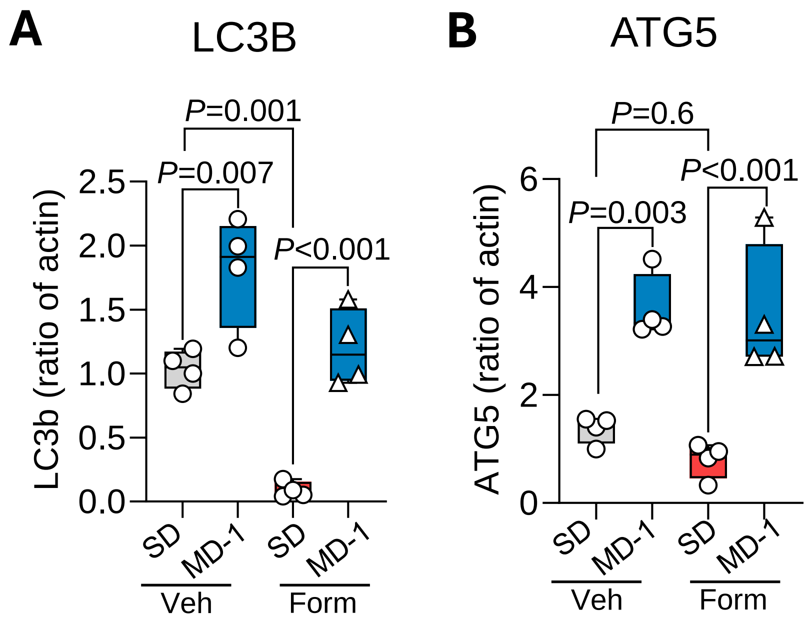

# Supplemental Fig. S9

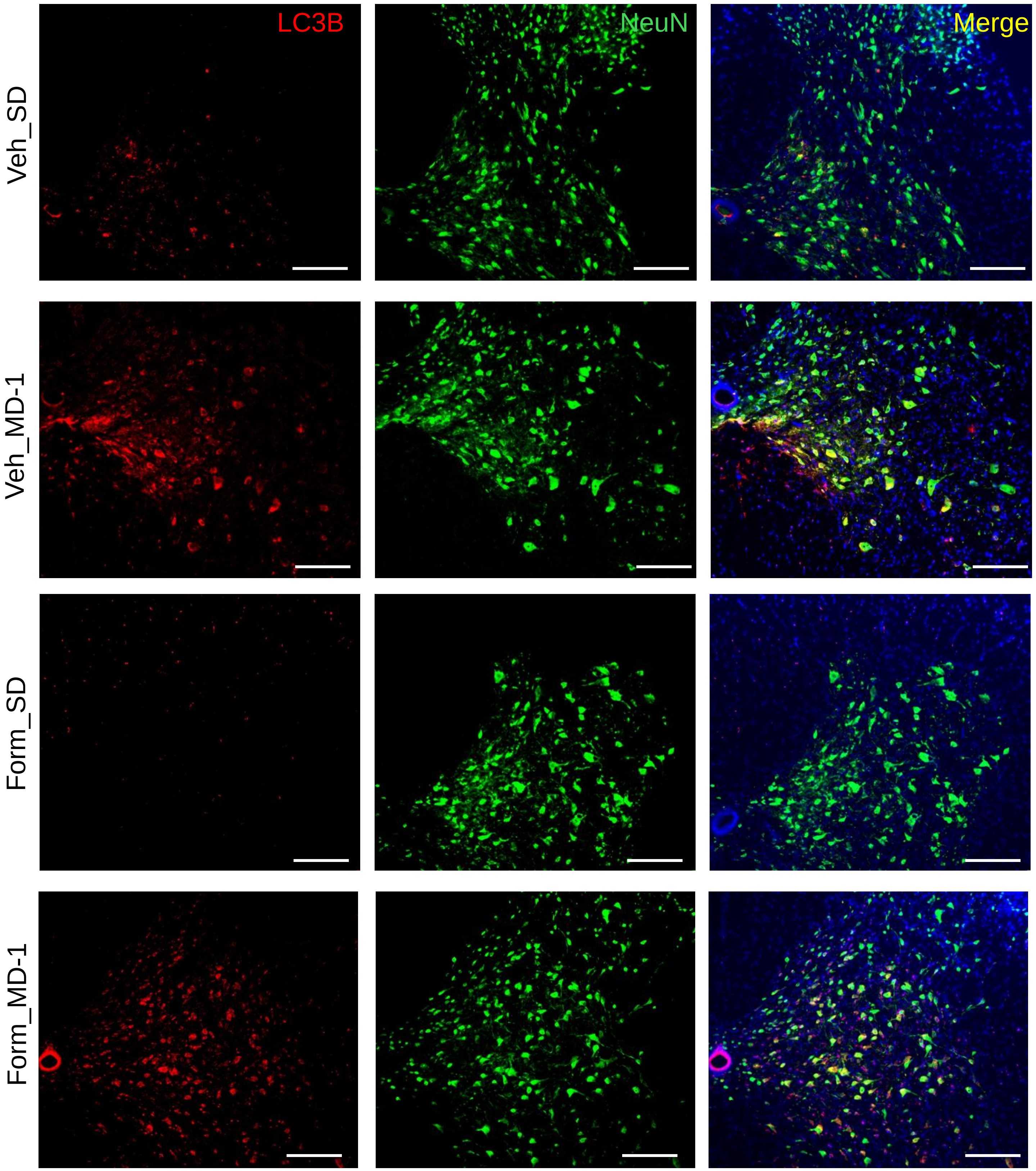

# Supplemental Fig. S10

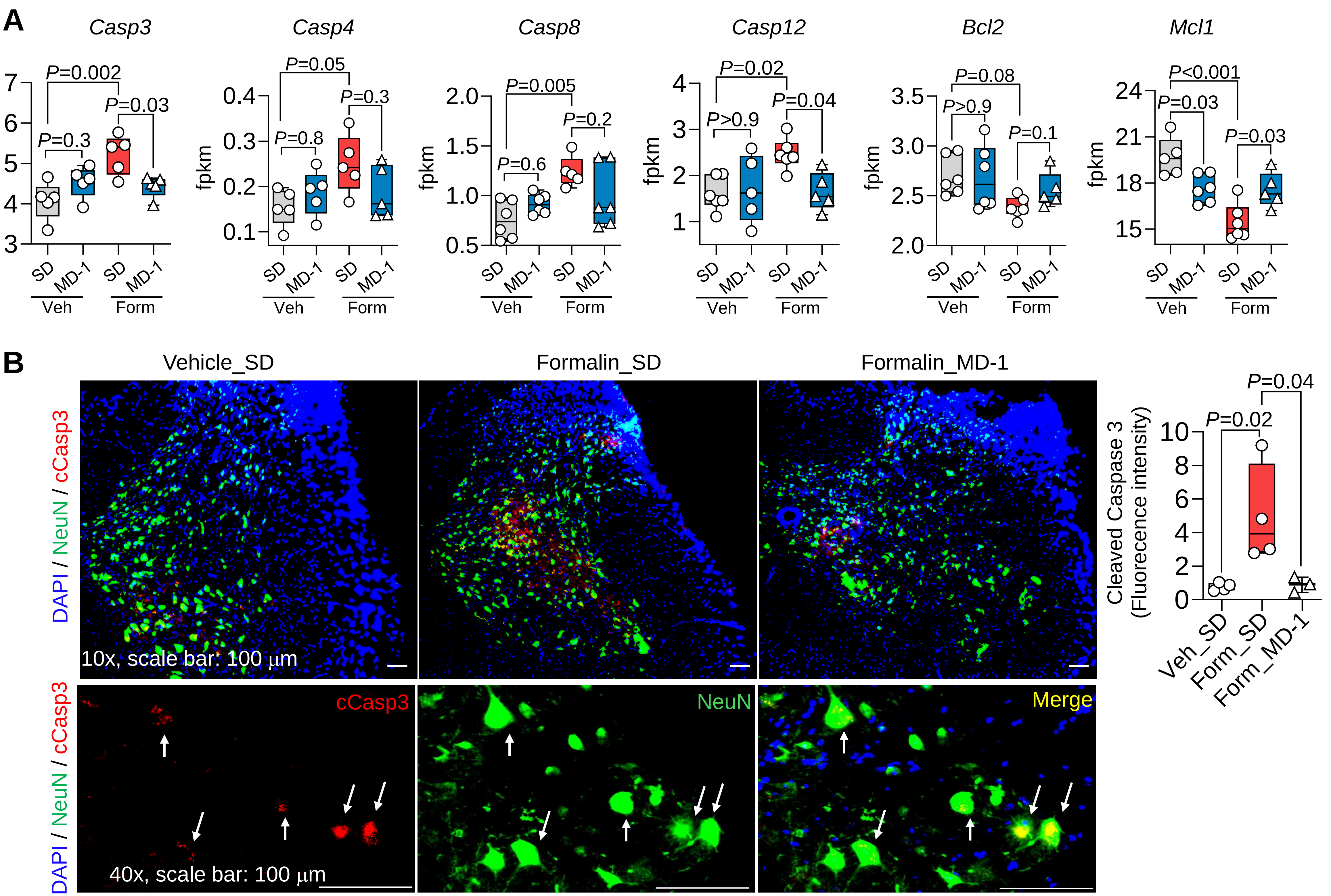

# Supplemental Fig. S11

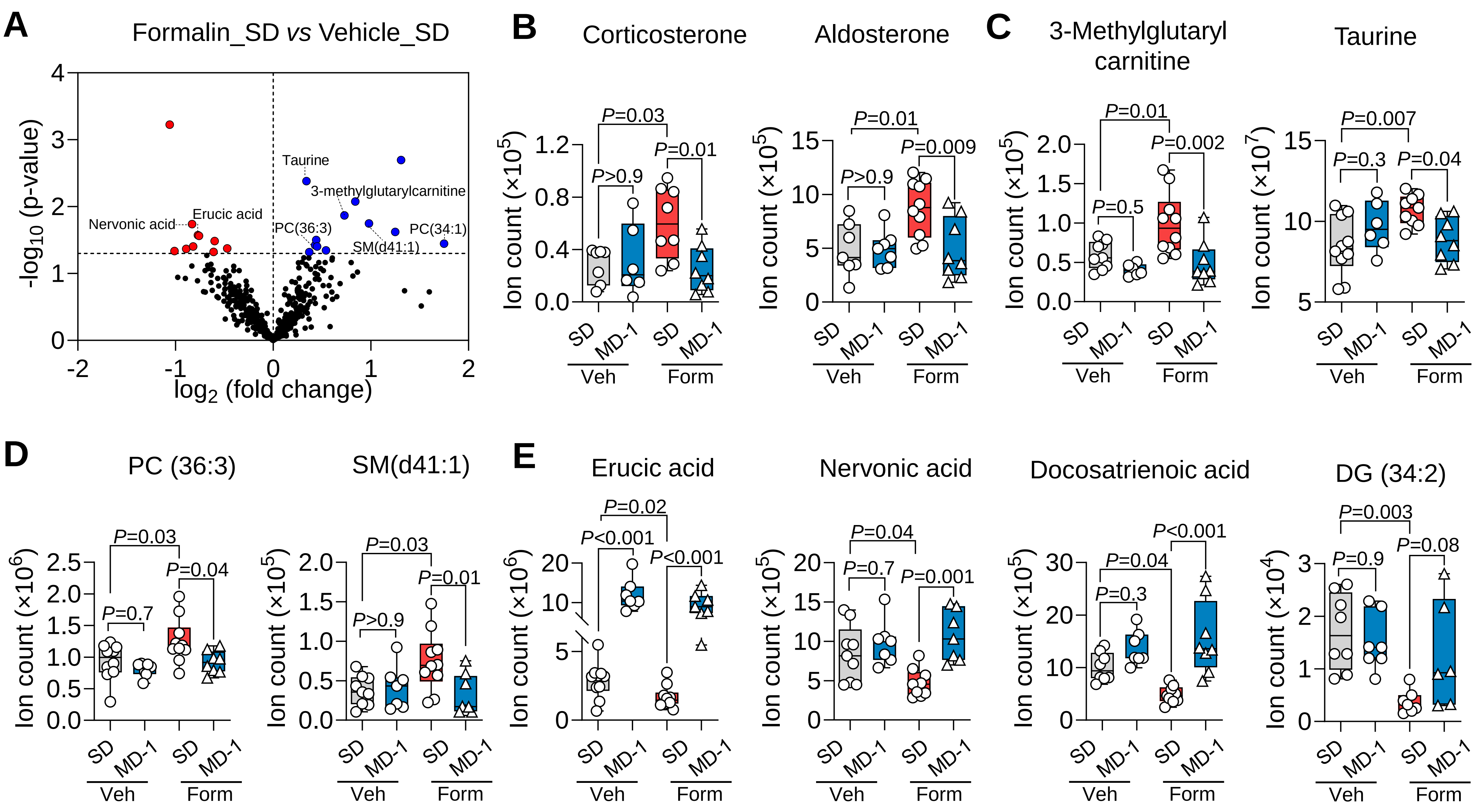

# Supplemental Fig. S12

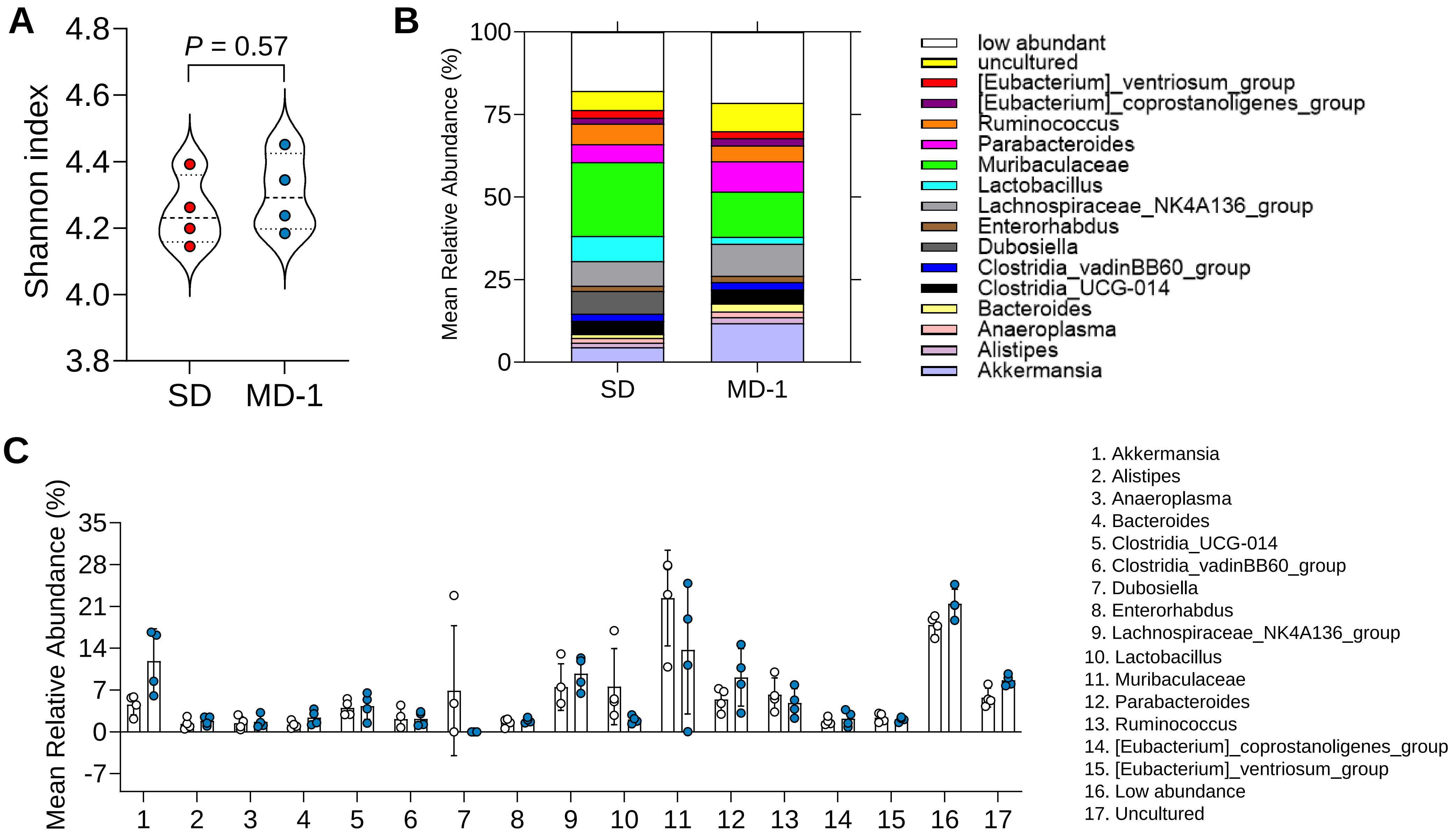

# Supplemental Fig. S13

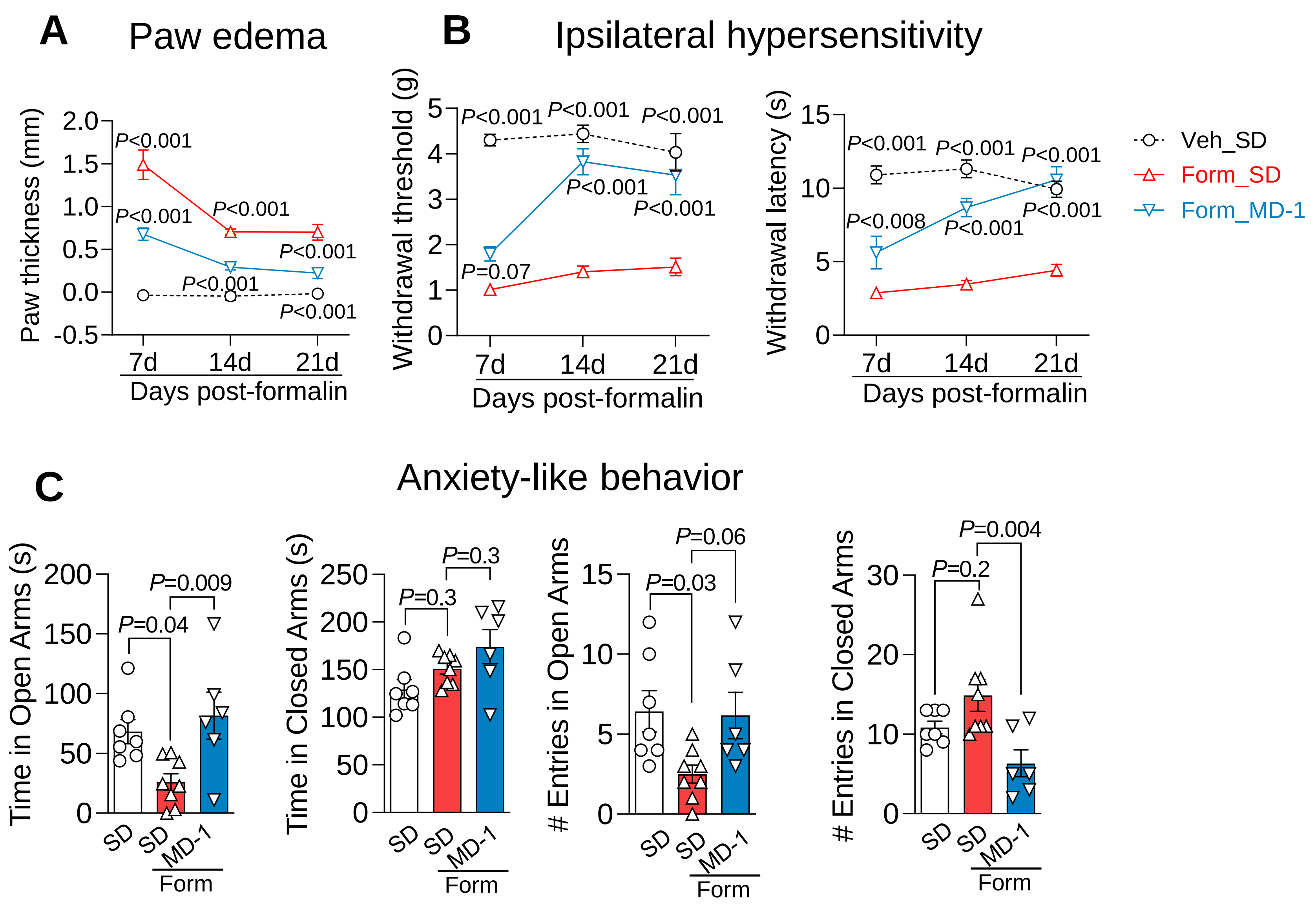

# Supplemental Fig. S14

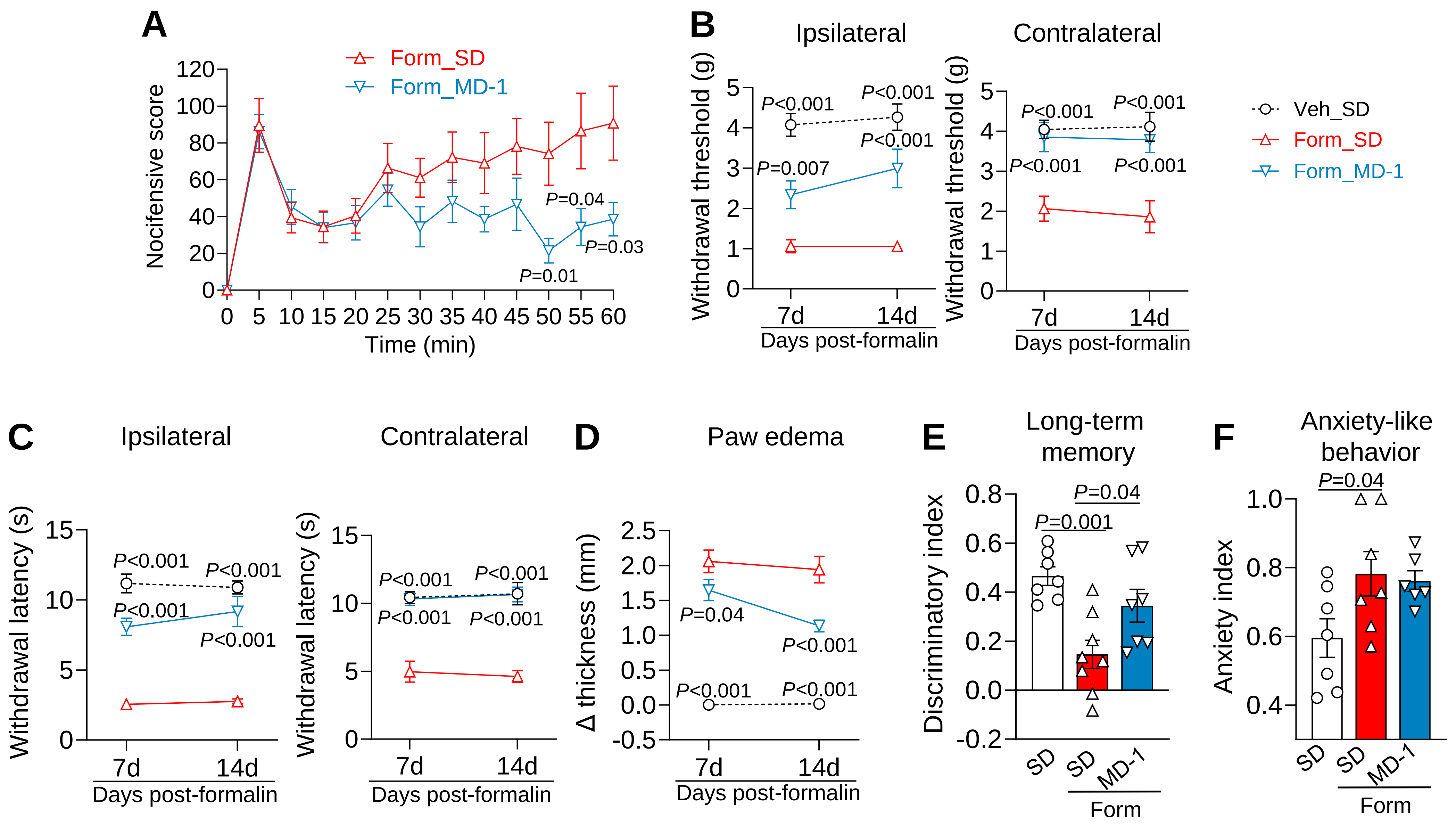

# Supplemental Fig. S15

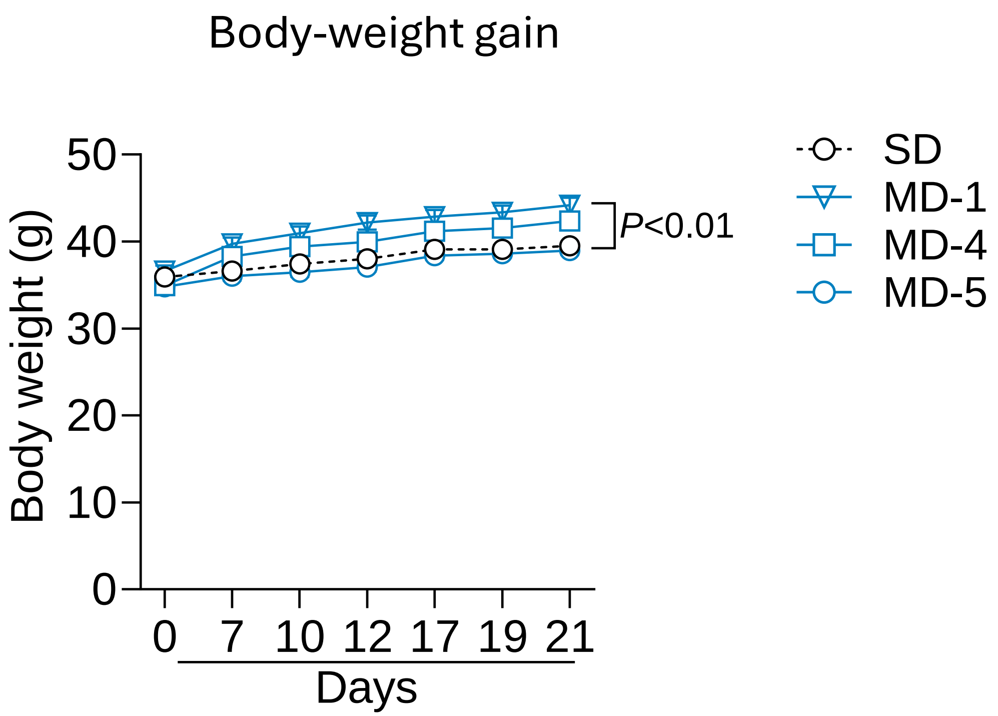

# Supplemental Fig. S16

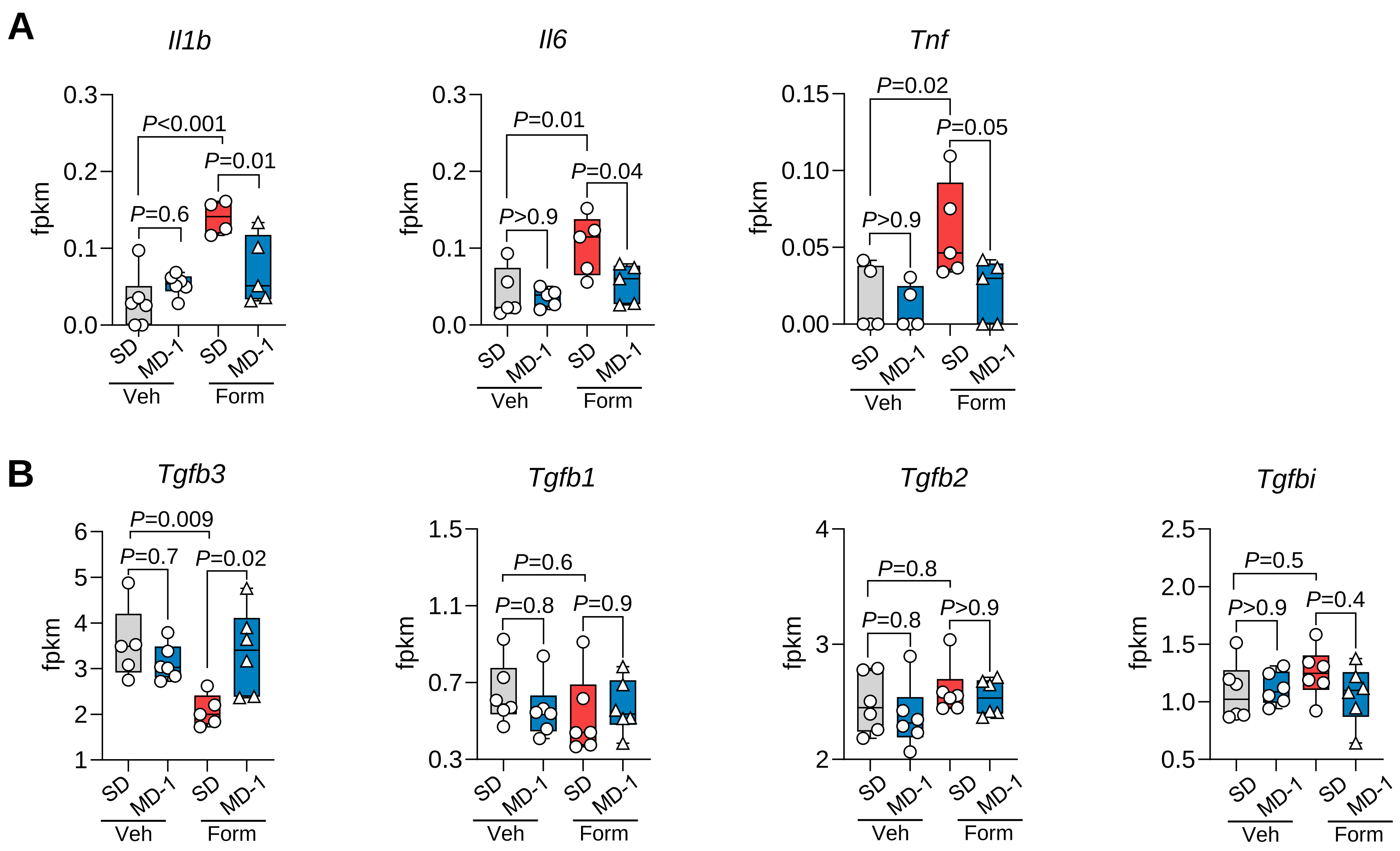

# Supplemental Fig. S17

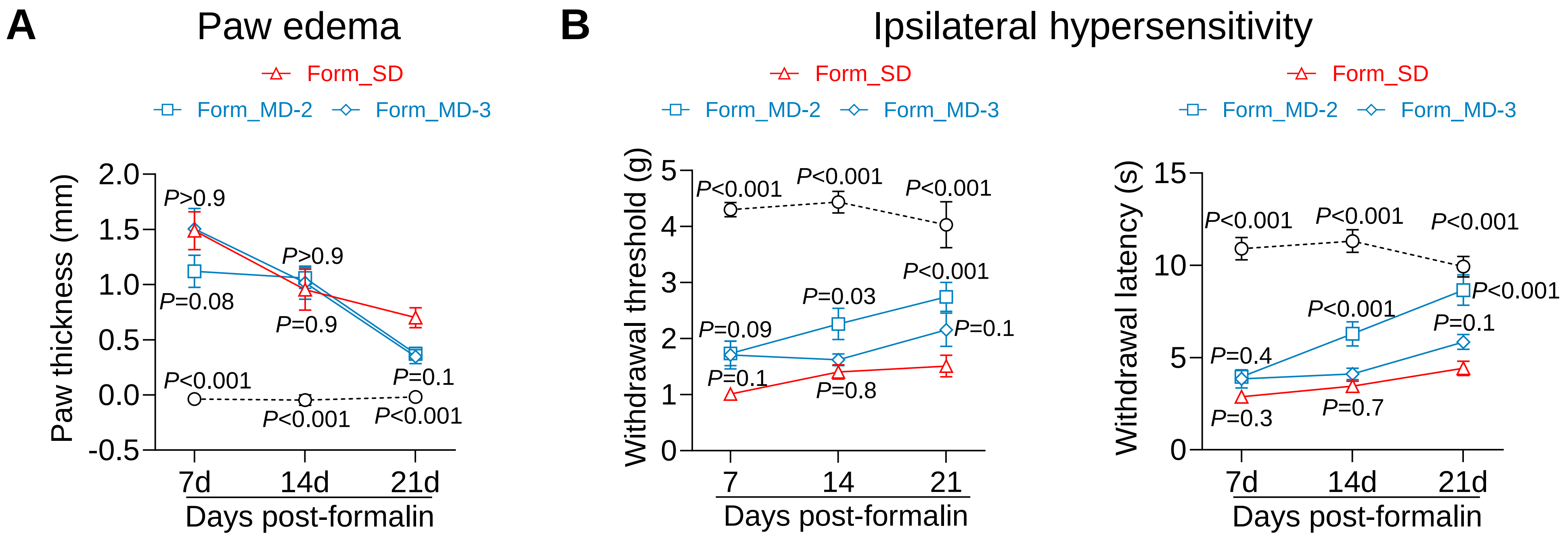

**Supplemental Fig. S18**

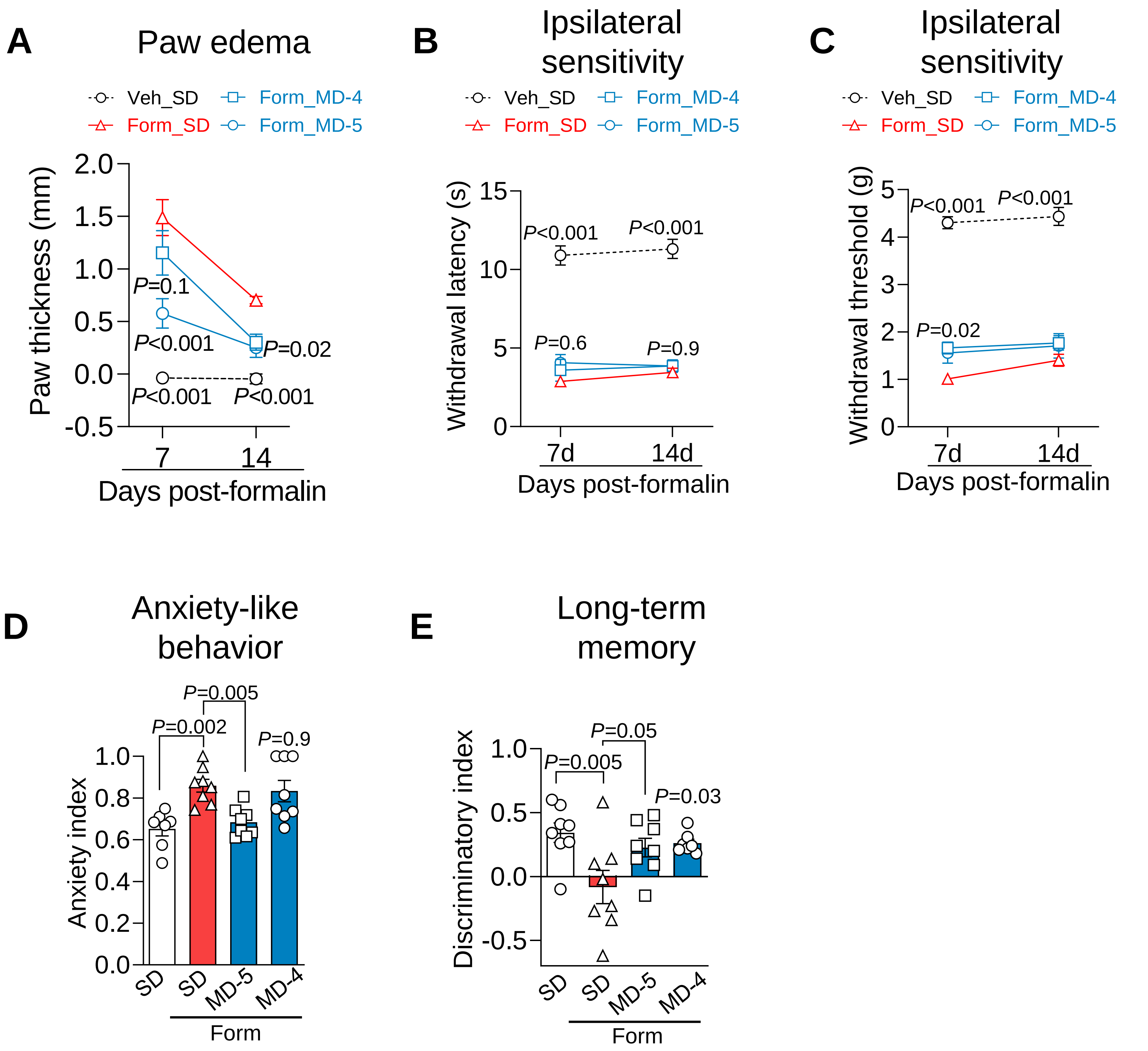

# Supplemental Fig. S19

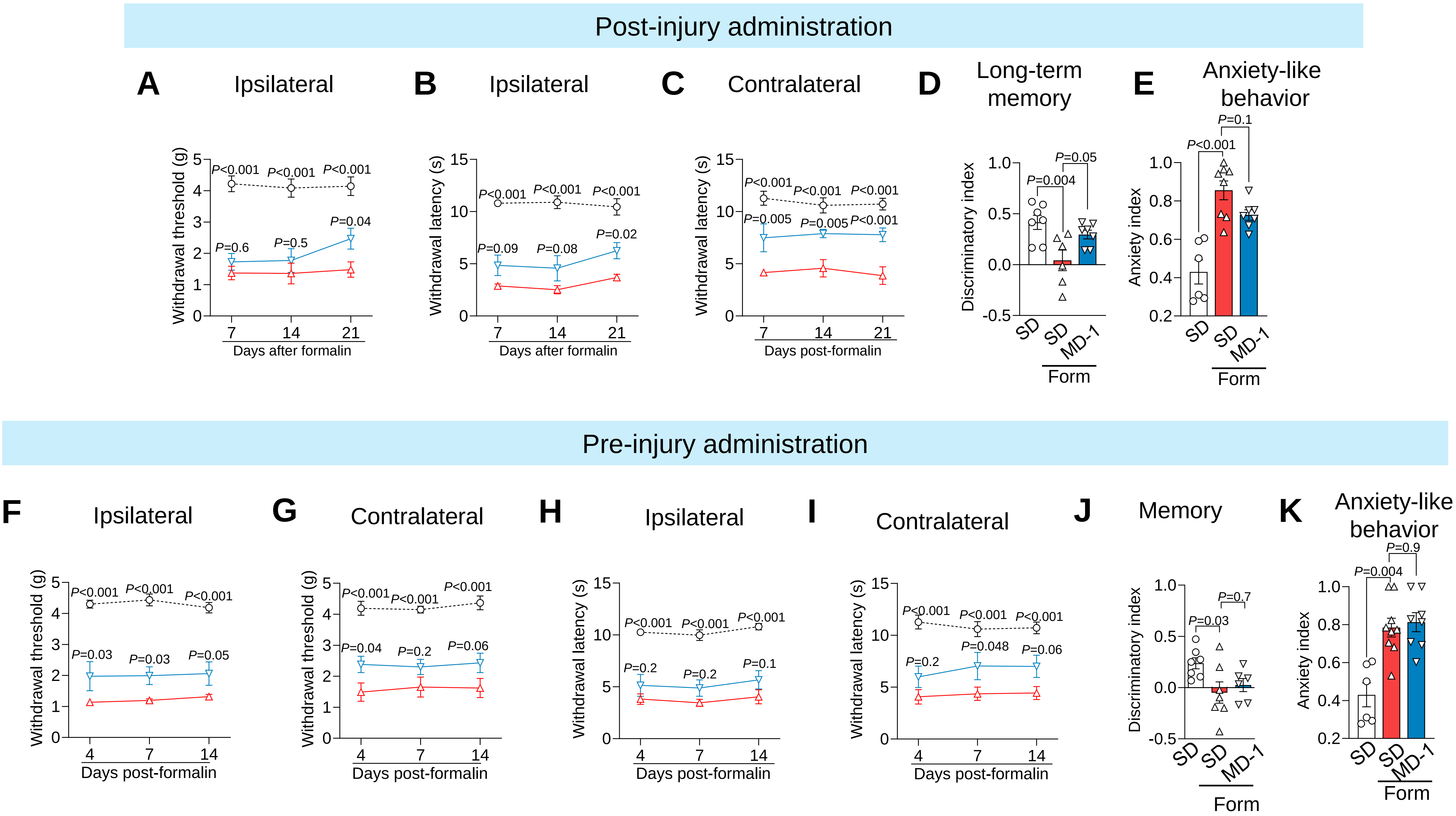

## Supplemental Fig. S20

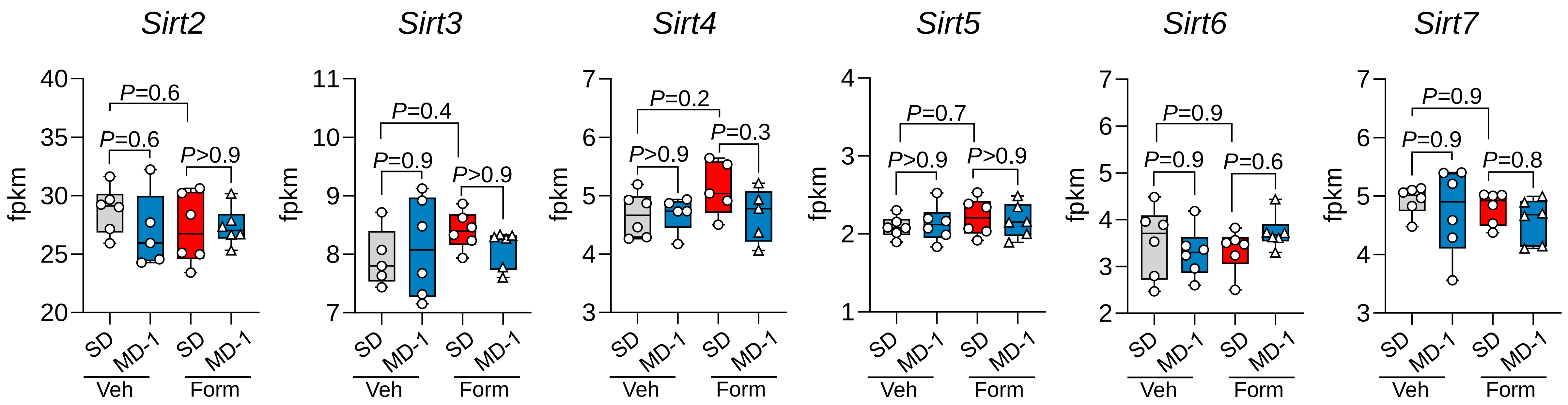

# Supplemental Fig. S21

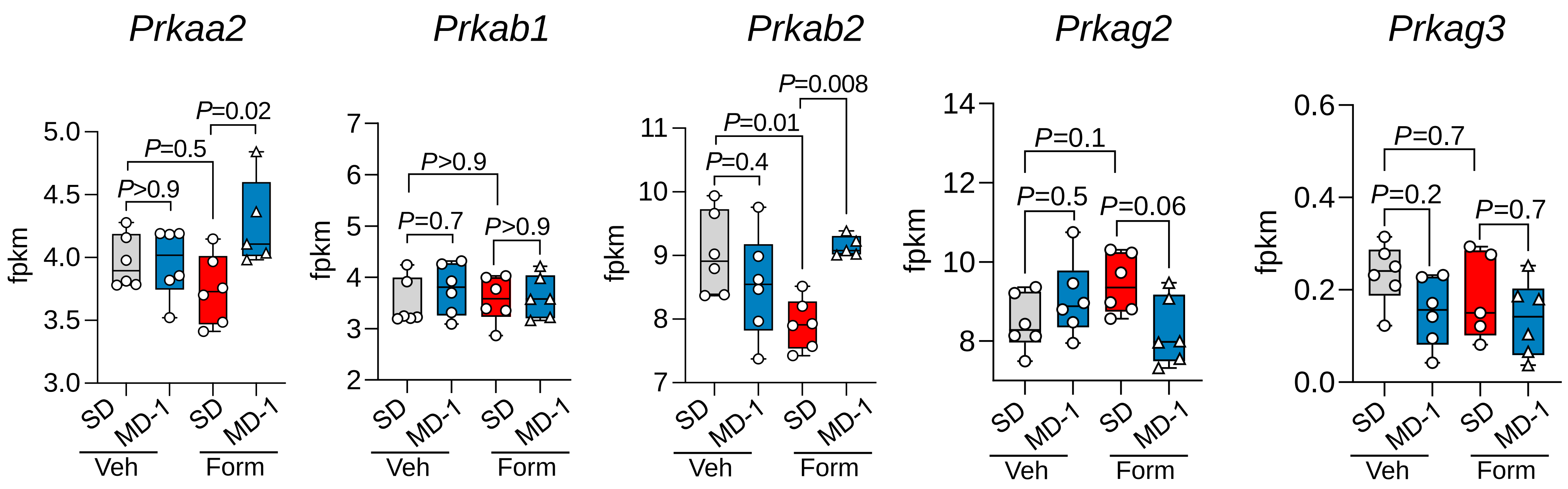

# Supplemental Fig. S22

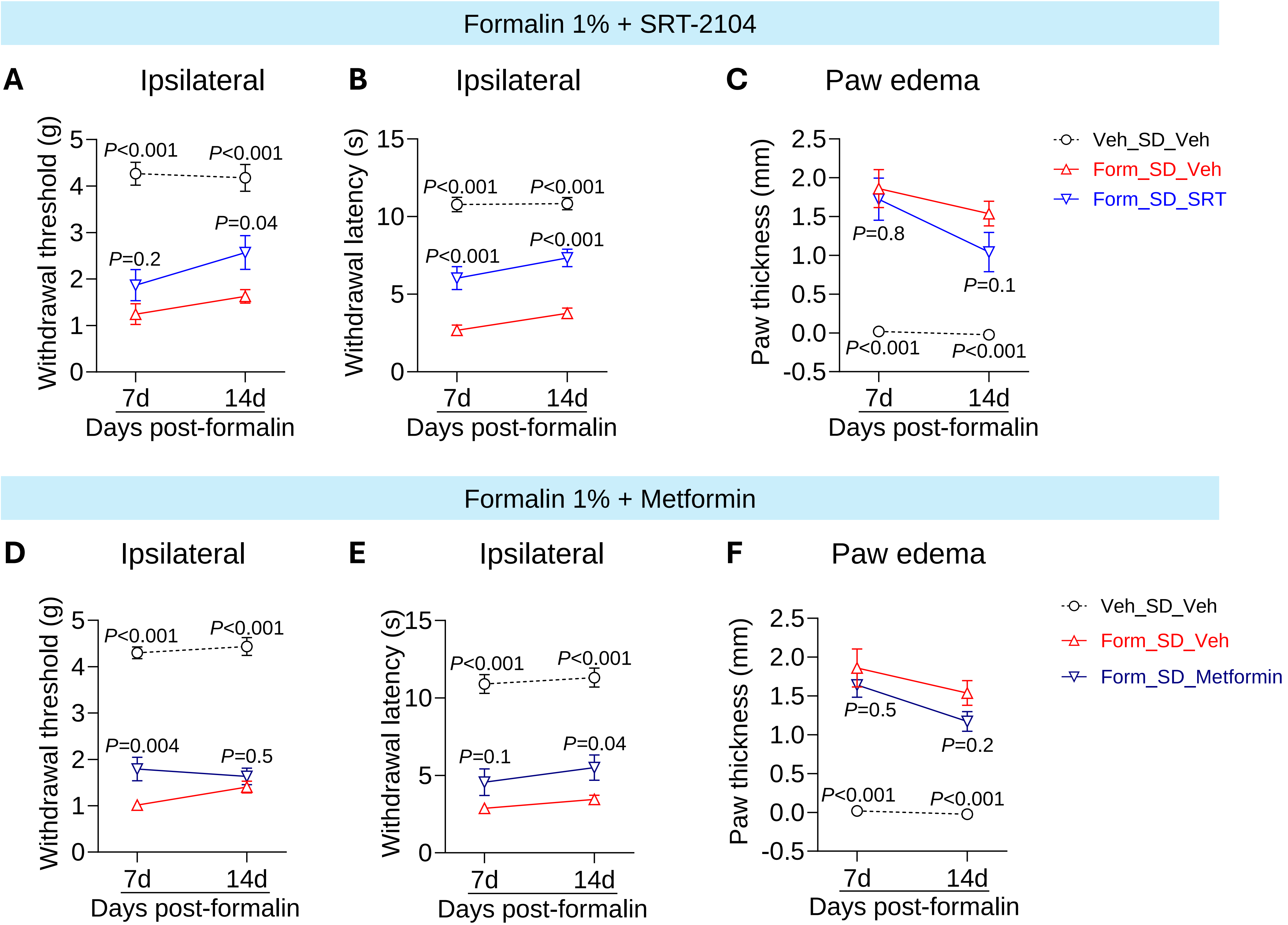

# Supplemental Fig. S23

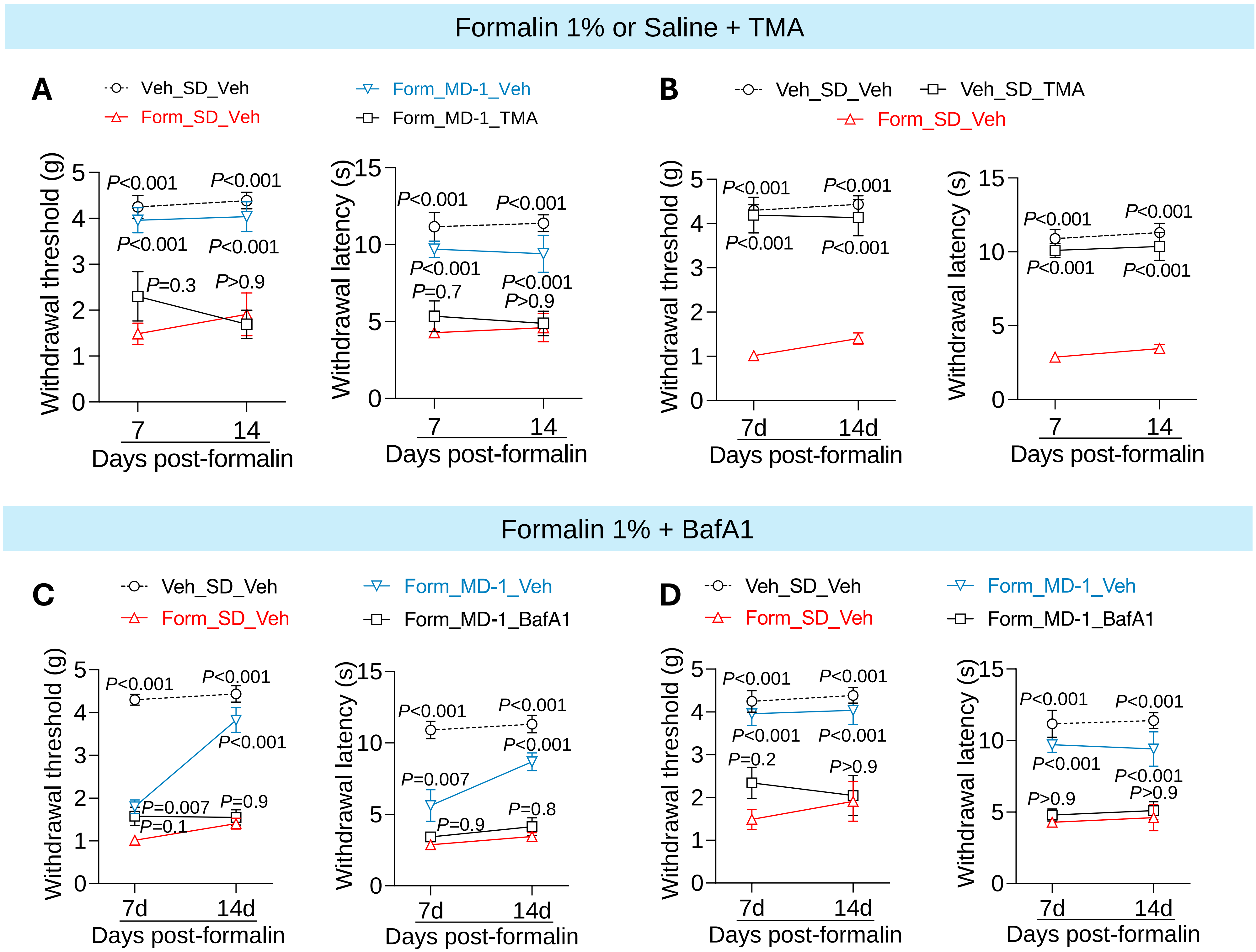

## Supplemental Fig. S24

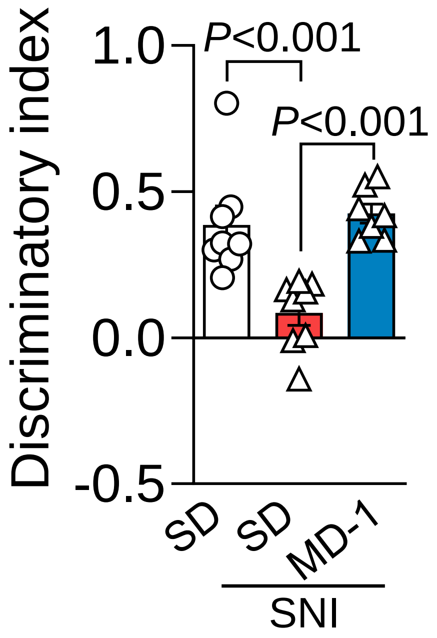

# Supplemental Fig. S25

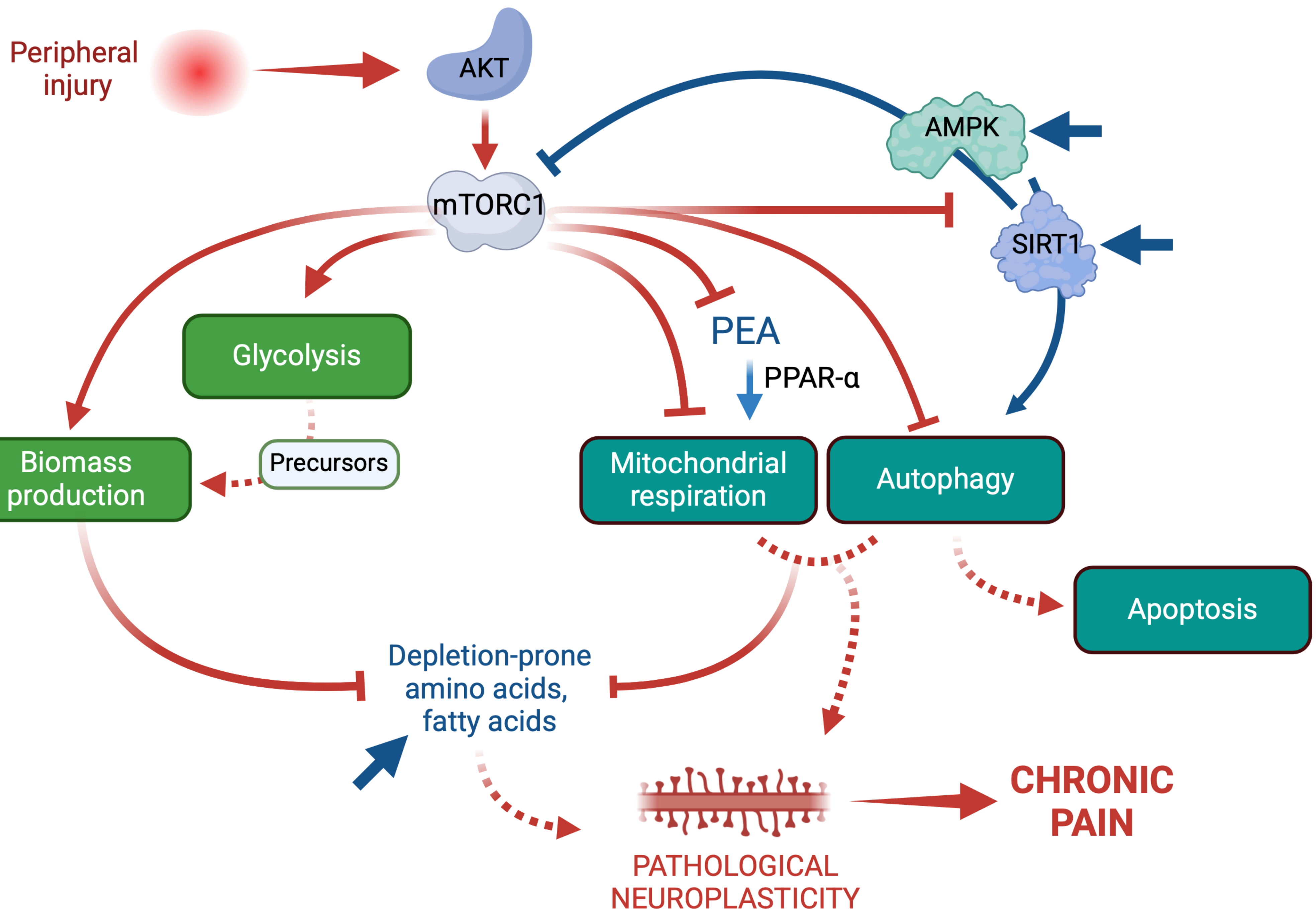

bioRxiv preprint doi: https://doi.org/10.1101/2025.01.30.635746; this version posted February 1, 2025. The copyright holder for this preprint (which was not certified by peer review) is the author/funder, who has granted bioRxiv a license to display the preprint in perpetuity. It is made available under aCC-BY 4.0 International license.

**Supplemental Table S1**.

**A.**

| Ingredient | SD | MD-1 | MD-2 | MD-3 | MD-4 | MD-5 |
|---|---|---|---|---|---|---|
| Ala | 1.20 | 2.80 | 1.40 | 0.70 | 2.80 | 1.20 |
| Thr | 0.60 | 1.76 | 0.88 | 0.44 | 1.76 | 0.60 |
| Pro | 1.90 | 3.84 | 1.92 | 0.96 | 3.84 | 1.90 |
| Ser | 0.90 | 1.68 | 0.84 | 0.42 | 1.68 | 0.90 |
| Leu | 2.30 | 4.85 | 2.43 | 1.21 | 4.85 | 2.30 |
| Ile | 0.70 | 2.98 | 1.49 | 0.75 | 2.98 | 0.70 |
| Val | 0.90 | 2.98 | 1.49 | 0.75 | 2.98 | 0.90 |
| Phe | 1.00 | 2.26 | 1.13 | 0.57 | 2.26 | 1.00 |
| Tyr | 0.50 | 1.28 | 0.64 | 0.32 | 1.28 | 0.50 |
| Met | 0.50 | 0.98 | 0.49 | 0.25 | 0.98 | 0.50 |
| Cys | 0.30 | 0.68 | 0.34 | 0.17 | 0.68 | 0.30 |
| Trp | 0.20 | 0.76 | 0.38 | 0.19 | 0.76 | 0.20 |
| Oleic acid | 1.10 | 6.06 | 3.03 | 1.52 | 6.06 | 1.10 |
| Erucic acid | 0.00 | 0.25 | 0.13 | 0.06 | 0.25 | 0.00 |
| PEA | 0.00 | 0.08 | 0.04 | 0.02 | 0.00 | 0.08 |

**B.**

| | Carbohydrate | Fat | Protein | Energy density | Daily intake (g/100g) |
|---|---|---|---|---|---|
| SD | 47.0 | 6.5 | 19.1 | 3.2 | $13.5 \pm 0.8$ |
| MD-1 | 35.7 | 10.2 | 33.0 | 3.7 | $11.8 \pm 1.9$ |
| MD-2 | 41.4 | 9.1 | 28.4 | 3.6 | ND |
| MD-3 | 44.2 | 7.8 | 23.7 | 3.4 | ND |
| MD-4 | 35.7 | 10.2 | 33.0 | 3.7 | ND |
| MD-5 | 47.0 | 6.6 | 19.1 | 3.2 | ND |

**Supplemental Table S2**.

| | Veh_SD | | Veh_MD-1 | | | |
|---|---|---|---|---|---|---|
| Compound Id | Mean | SEM | Mean | SEM | $Log_2$ (FC) | *P*-value |
| DG(18:1(11Z)/20:5(5Z,8Z,11Z,14Z,17Z)/0:0) | 2.50E+04 | 6.79E+03 | 8.09E+03 | 1.65E+03 | -1.63 | 3.63E-02 |
| 5-Nonadecyl-1,3-benzenediol | 2.56E+05 | 4.86E+04 | 9.10E+04 | 1.82E+04 | -1.49 | 8.48E-03 |
| Isocaucalol isomer | 1.17E+06 | 2.95E+05 | 4.27E+05 | 7.37E+04 | -1.45 | 3.46E-02 |
| (S)-Homostachydrine | 1.29E+06 | 1.10E+05 | 4.87E+05 | 4.26E+04 | -1.41 | 2.29E-05 |
| Perillic acid | 1.14E+06 | 1.61E+05 | 4.45E+05 | 7.99E+04 | -1.35 | 2.06E-03 |
| Palmitoylcarnitine | 1.29E+06 | 1.50E+05 | 5.10E+05 | 5.39E+04 | -1.34 | 4.40E-04 |
| 9-Decenoylcarnitine | 1.18E+04 | 1.85E+03 | 5.13E+03 | 3.62E+02 | -1.20 | 5.62E-03 |
| Indole-3-methyl acetate | 3.36E+05 | 7.15E+04 | 1.51E+05 | 1.63E+04 | -1.16 | 3.03E-02 |
| (5Z,8Z)-Tetradecadienoylcarnitine | 1.06E+05 | 1.67E+04 | 4.76E+04 | 4.65E+03 | -1.15 | 7.16E-03 |
| 3',4',5'-Trimethoxycinnamyl alcohol acetate | 6.00E+05 | 6.64E+04 | 2.78E+05 | 3.94E+04 | -1.11 | 9.66E-04 |
| Myristoylcarnitine | 2.39E+05 | 3.02E+04 | 1.13E+05 | 1.75E+04 | -1.08 | 2.90E-03 |
| Isolinderanolide | 4.65E+05 | 7.74E+04 | 2.19E+05 | 1.67E+04 | -1.08 | 1.16E-02 |
| DL-2-Aminooctanoic acid | 1.17E+05 | 1.43E+04 | 5.55E+04 | 5.35E+03 | -1.07 | 1.95E-03 |
| PI(16:0/18:2(9Z,12Z)) | 1.55E+05 | 2.50E+04 | 7.36E+04 | 1.05E+04 | -1.07 | 1.15E-02 |
| 3-Oxododecanoic acid | 1.66E+06 | 2.75E+05 | 7.98E+05 | 1.11E+05 | -1.05 | 1.37E-02 |
| Linoleic acid | 6.64E+08 | 1.28E+08 | 3.26E+08 | 3.54E+07 | -1.03 | 2.84E-02 |
| Tetradecanedioic acid | 4.21E+04 | 6.59E+03 | 2.08E+04 | 2.62E+03 | -1.02 | 1.13E-02 |
| (2R,3R,4R)-2-Amino-4-hydroxy-3-methylpentanoic acid | 3.79E+05 | 5.56E+04 | 1.91E+05 | 2.52E+04 | -0.99 | 9.27E-03 |
| (10Z,12Z)-octadeca-10,12-dienoylcarnitine | 2.90E+05 | 4.10E+04 | 1.47E+05 | 1.45E+04 | -0.98 | 7.20E-03 |
| Podecdysone b isomer | 4.62E+05 | 6.58E+04 | 2.38E+05 | 3.77E+04 | -0.95 | 1.08E-02 |
| Stearoylcarnitine | 1.45E+05 | 1.25E+04 | 7.68E+04 | 9.41E+03 | -0.92 | 5.50E-04 |
| SM(d16:1/24:1(15Z)) | 7.62E+05 | 1.13E+05 | 4.05E+05 | 6.65E+04 | -0.91 | 1.68E-02 |
| 2-Cyclohexen-1-one, 3-(15-hydroxypentadecyl)-2,4,4-trimethyl- | 3.23E+04 | 5.56E+03 | 1.72E+04 | 2.44E+03 | -0.91 | 2.81E-02 |
| PG(16:0/18:2(9Z,12Z)) | 3.87E+04 | 5.38E+03 | 2.06E+04 | 2.38E+03 | -0.91 | 9.65E-03 |

| | | | | | | |
|---|---|---|---|---|---|---|
| N2-Acetylornithine | 6.95E+03 | 1.13E+03 | 3.73E+03 | 4.20E+02 | -0.90 | 2.16E-02 |
| DL-Stachydrine | 2.02E+07 | 1.89E+06 | 1.09E+07 | 7.67E+05 | -0.89 | 6.85E-04 |
| Proline betaine | 2.20E+06 | 1.46E+05 | 1.23E+06 | 6.22E+04 | -0.84 | 5.31E-05 |
| 5-Dodecenoylcarnitine | 4.75E+04 | 7.27E+03 | 2.68E+04 | 4.01E+03 | -0.83 | 2.59E-02 |
| Octadeca-2,4,6,8-tetraenoic acid | 2.05E+05 | 3.71E+04 | 1.19E+05 | 1.00E+04 | -0.79 | 4.80E-02 |
| PE(P-18:0/22:4(7Z,10Z,13Z,16Z)) | 3.35E+04 | 5.74E+03 | 1.94E+04 | 3.07E+03 | -0.79 | 4.94E-02 |
| PE(20:2(11Z,14Z)/15:0) | 3.88E+05 | 3.22E+04 | 2.31E+05 | 1.93E+04 | -0.75 | 9.10E-04 |
| Adrenic acid | 9.05E+06 | 1.51E+06 | 5.46E+06 | 3.98E+05 | -0.73 | 4.39E-02 |
| Tetracosahexaenoic acid | 1.66E+06 | 2.17E+05 | 1.03E+06 | 1.37E+05 | -0.69 | 2.74E-02 |
| 4-Guanidinobutanoic acid | 2.32E+05 | 2.88E+04 | 1.46E+05 | 2.04E+04 | -0.68 | 2.68E-02 |
| 9-Hexadecenoylcarnitine | 1.85E+05 | 2.89E+04 | 1.16E+05 | 1.29E+04 | -0.67 | 4.96E-02 |
| Ferulic acid 4-O-sulfate | 1.83E+06 | 1.75E+05 | 1.18E+06 | 1.57E+05 | -0.63 | 1.51E-02 |
| 2-Methoxyestradiol-3-methylether | 2.31E+04 | 2.47E+03 | 1.54E+04 | 1.80E+03 | -0.58 | 2.44E-02 |
| PG(18:0/16:0) | 4.60E+04 | 5.31E+03 | 3.14E+04 | 3.48E+03 | -0.55 | 3.71E-02 |
| PC(18:2(9Z,12Z)/14:0) | 1.98E+06 | 2.10E+05 | 1.41E+06 | 8.64E+04 | -0.49 | 2.83E-02 |
| Argininosuccinic acid | 4.79E+03 | 5.09E+02 | 3.47E+03 | 3.42E+02 | -0.46 | 4.96E-02 |
| L-Carnitine | 2.22E+08 | 1.40E+07 | 1.61E+08 | 1.06E+07 | -0.46 | 3.52E-03 |
| PC(18:2(9Z,12Z)/16:1(9Z)) | 4.59E+06 | 4.18E+05 | 3.54E+06 | 2.36E+05 | -0.37 | 4.74E-02 |
| Indoleacrylic acid | 3.01E+07 | 2.46E+06 | 3.69E+07 | 1.98E+06 | 0.29 | 4.91E-02 |
| 8-Hydroxyquinoline | 2.74E+06 | 2.64E+05 | 3.44E+06 | 1.99E+05 | 0.33 | 4.84E-02 |
| 1,5-Naphthalenediamine | 2.02E+06 | 1.84E+05 | 2.61E+06 | 1.30E+05 | 0.37 | 1.89E-02 |
| Methylindole isomer | 5.97E+05 | 5.26E+04 | 8.06E+05 | 5.22E+04 | 0.43 | 1.34E-02 |
| 3-Indoleacetonitrile | 6.23E+05 | 6.05E+04 | 8.62E+05 | 5.16E+04 | 0.47 | 8.84E-03 |
| PC(22:5(4Z,7Z,10Z,13Z,16Z)/16:0) | 1.26E+07 | 1.67E+06 | 1.77E+07 | 9.69E+05 | 0.48 | 2.11E-02 |
| D-(+)-Proline | 2.47E+07 | 1.60E+06 | 3.46E+07 | 2.53E+06 | 0.49 | 7.33E-03 |
| PE(22:2(13Z,16Z)/18:1(11Z)) | 3.62E+05 | 3.49E+04 | 5.22E+05 | 4.13E+04 | 0.53 | 1.09E-02 |
| LysoPC(24:1(15Z)/0:0) | 1.55E+05 | 2.13E+04 | 2.27E+05 | 2.11E+04 | 0.56 | 2.87E-02 |
| PE(18:2(9Z,12Z)/18:1(9Z)) | 1.61E+05 | 2.21E+04 | 2.40E+05 | 2.47E+04 | 0.58 | 3.07E-02 |

| | | | | | | |
|---|---|---|---|---|---|---|
| Galactitol | 8.91E+05 | 6.94E+04 | 1.36E+06 | 1.62E+05 | 0.62 | 2.67E-02 |
| Cer(d18:1/24:1(15Z)) | 1.12E+05 | 1.54E+04 | 1.74E+05 | 1.47E+04 | 0.64 | 1.06E-02 |
| Elaidic acid | 5.63E+08 | 9.07E+07 | 8.87E+08 | 1.09E+08 | 0.66 | 3.93E-02 |
| Deoxyadenosine monophosphate | 2.89E+03 | 3.87E+02 | 4.60E+03 | 4.51E+02 | 0.67 | 1.25E-02 |
| 3-Methylcrotonylglycine | 5.48E+04 | 8.34E+03 | 8.80E+04 | 8.73E+03 | 0.68 | 1.54E-02 |
| Docosatrienoic acid | 8.51E+05 | 1.31E+05 | 1.37E+06 | 1.22E+05 | 0.69 | 1.10E-02 |
| PC(18:2(9Z,12Z)/18:1(11Z)) | 3.13E+07 | 4.65E+06 | 5.08E+07 | 2.63E+06 | 0.70 | 2.66E-03 |
| Indole-3-carboxilic acid-sulphate isomer | 7.13E+05 | 1.08E+05 | 1.19E+06 | 1.62E+05 | 0.74 | 3.07E-02 |
| L-Allothreonine | 6.40E+06 | 4.48E+05 | 1.09E+07 | 7.04E+05 | 0.76 | 2.61E-04 |
| LysoPC(P-18:0/0:0) | 3.06E+05 | 3.74E+04 | 5.39E+05 | 4.85E+04 | 0.82 | 2.40E-03 |
| lysops(22:0) | 5.58E+06 | 8.13E+05 | 9.83E+06 | 7.56E+05 | 0.82 | 1.68E-03 |
| LysoPC(18:1(9Z)/0:0) | 5.45E+07 | 7.92E+06 | 9.64E+07 | 1.01E+07 | 0.82 | 6.45E-03 |
| Annoglabasin F | 1.22E+06 | 1.82E+05 | 2.15E+06 | 2.31E+05 | 0.82 | 7.46E-03 |
| 3-Hydroxybutyrylcarnitine | 2.18E+05 | 1.70E+04 | 3.95E+05 | 4.51E+04 | 0.86 | 6.60E-03 |
| PC(18:1(9Z)/15:0) | 2.47E+05 | 2.87E+04 | 4.53E+05 | 4.49E+04 | 0.87 | 2.75E-03 |
| DG(18:2(9Z,12Z)/18:3(6Z,9Z,12Z)/0:0) | 4.21E+04 | 8.44E+03 | 7.72E+04 | 1.29E+04 | 0.88 | 4.36E-02 |
| Indoxyl sulfate | 6.87E+06 | 8.78E+05 | 1.26E+07 | 1.39E+06 | 0.88 | 5.39E-03 |
| LysoPC(22:1(13Z)/0:0) | 3.00E+05 | 4.43E+04 | 5.62E+05 | 5.74E+04 | 0.91 | 3.39E-03 |
| 5-Aminovaleric acid | 5.37E+07 | 4.68E+06 | 1.01E+08 | 1.17E+07 | 0.91 | 5.58E-03 |
| PI(20:4(8Z,11Z,14Z,17Z)/18:1(9Z)) | 8.97E+04 | 1.72E+04 | 1.71E+05 | 2.43E+04 | 0.93 | 1.90E-02 |
| PE(18:0/18:1(9Z)) | 3.06E+05 | 2.98E+04 | 5.95E+05 | 3.31E+04 | 0.96 | 1.62E-05 |
| LysoPE(0:0/18:1(11Z)) | 1.01E+06 | 1.29E+05 | 2.02E+06 | 1.99E+05 | 1.01 | 1.32E-03 |
| 5-Hydroxyindole | 1.15E+05 | 1.32E+04 | 2.31E+05 | 2.58E+04 | 1.01 | 2.90E-03 |
| N-Acetyltryptophan | 3.22E+05 | 1.61E+04 | 6.60E+05 | 6.54E+04 | 1.04 | 1.71E-03 |
| Leu asp isomer | 1.23E+05 | 1.05E+04 | 2.73E+05 | 3.14E+04 | 1.15 | 2.34E-03 |
| Pemoline | 5.26E+03 | 5.88E+02 | 1.29E+04 | 1.15E+03 | 1.29 | 2.19E-04 |
| Tiglylcarnitine | 6.70E+03 | 7.17E+02 | 1.75E+04 | 1.44E+03 | 1.39 | 8.75E-05 |
| PE(18:2(9Z,12Z)/24:1(15Z)) | 4.39E+04 | 8.23E+03 | 1.19E+05 | 2.54E+04 | 1.44 | 2.53E-02 |

| | | | | | | |
|---|---|---|---|---|---|---|
| PE(16:0/18:1(9Z)) | 2.64E+04 | 3.78E+03 | 7.42E+04 | 1.44E+04 | 1.49 | 1.54E-02 |
| PC(18:1(9Z)/20:2(11Z,14Z)) | 1.75E+06 | 4.36E+05 | 5.63E+06 | 4.73E+05 | 1.69 | 3.15E-05 |
| PE(16:1(9Z)/22:0) | 2.59E+05 | 2.69E+04 | 8.78E+05 | 7.72E+04 | 1.76 | 9.25E-05 |
| Cresol sulfate isomer | 3.84E+06 | 1.15E+06 | 1.41E+07 | 1.56E+06 | 1.87 | 2.01E-04 |
| Guanosine | 2.44E+04 | 7.51E+03 | 1.07E+05 | 2.73E+04 | 2.14 | 2.26E-02 |
| 1-Pentanesulfenothioic acid | 1.77E+06 | 4.19E+05 | 1.39E+07 | 2.77E+06 | 2.98 | 4.45E-03 |
| Inosine | 1.31E+06 | 3.22E+05 | 1.16E+07 | 2.32E+06 | 3.15 | 4.24E-03 |
| 2-(3,4-Dihydroxybenzoyloxy)-4,6-dihydroxybenzoate | 3.22E+04 | 8.81E+03 | 3.96E+05 | 9.37E+04 | 3.62 | 8.00E-03 |
| PC(18:1(9Z)/16:0) | 7.02E+05 | 2.85E+05 | 1.59E+07 | 2.17E+06 | 4.50 | 3.76E-04 |
| Xanthine | 1.07E+06 | 4.62E+05 | 3.17E+07 | 1.17E+07 | 4.89 | 4.04E-02 |
| PE(18:0/22:1(13Z)) | 4.41E+03 | 9.38E+02 | 2.48E+05 | 3.72E+04 | 5.81 | 6.14E-04 |

**Supplemental Table S3**.

| | Veh_SD | | Veh_MD-1 | | | |
|---|---|---|---|---|---|---|
| Compound Id | Mean | SEM | Mean | SEM | $Log_2$ (FC) | *P*-value |
| Methionine sulfoxide | 3.87E+03 | 8.83E+02 | 8.62E+02 | 1.14E+02 | -2.17 | 7.78E-03 |
| Glutarylcarnitine | 5.12E+05 | 6.26E+04 | 2.76E+05 | 3.53E+04 | -0.89 | 5.71E-03 |
| Saccharopine | 3.66E+06 | 4.28E+05 | 2.01E+06 | 3.21E+05 | -0.87 | 7.40E-03 |
| N2-Acetylornithine | 4.03E+03 | 3.38E+02 | 2.31E+03 | 5.67E+02 | -0.80 | 2.60E-02 |
| Proline betaine | 1.18E+05 | 7.84E+03 | 7.30E+04 | 7.45E+03 | -0.69 | 9.38E-04 |
| PS(18:0/18:2(9Z,12Z)) | 2.22E+06 | 2.13E+05 | 1.39E+06 | 1.00E+05 | -0.67 | 3.88E-03 |
| DL-2-Aminooctanoic acid | 1.10E+05 | 5.12E+03 | 6.96E+04 | 5.14E+03 | -0.67 | 5.66E-05 |
| LysoPE(0:0/20:2(11Z,14Z)) | 4.02E+04 | 3.34E+03 | 2.73E+04 | 3.35E+03 | -0.56 | 1.65E-02 |
| (10Z,12Z)-octadeca-10,12-dienoylcarnitine | 4.79E+05 | 1.99E+04 | 3.26E+05 | 2.50E+04 | -0.55 | 3.88E-04 |
| L-Pipecolic acid | 4.22E+04 | 1.18E+03 | 2.88E+04 | 1.26E+03 | -0.55 | 2.04E-06 |
| 3,4-Dihydroxyhydrocinnamic acid | 1.42E+06 | 1.33E+05 | 9.90E+05 | 1.08E+05 | -0.52 | 2.52E-02 |
| D-Glucuronic acid | 7.82E+03 | 3.99E+02 | 5.49E+03 | 5.94E+02 | -0.51 | 7.41E-03 |
| PE(18:0/18:3(9Z,12Z,15Z)) | 3.05E+05 | 2.05E+04 | 2.14E+05 | 1.36E+04 | -0.51 | 2.27E-03 |
| Taurodeoxycholic acid | 1.68E+04 | 1.72E+03 | 1.19E+04 | 1.35E+03 | -0.50 | 4.08E-02 |
| Isolinderanolide | 2.17E+06 | 1.35E+05 | 1.56E+06 | 1.17E+05 | -0.48 | 3.47E-03 |
| 4-Hydroxyproline | 2.71E+05 | 2.03E+04 | 2.00E+05 | 1.86E+04 | -0.44 | 2.17E-02 |
| (Ã‚Â±)-2-Hydroxy-4-(methylthio)butanoic acid | 1.56E+04 | 1.09E+03 | 1.16E+04 | 1.01E+03 | -0.43 | 1.63E-02 |
| Allantoin | 8.62E+05 | 4.91E+04 | 6.47E+05 | 3.12E+04 | -0.41 | 2.38E-03 |
| Stoloniferone c isomer | 7.10E+05 | 5.60E+04 | 5.44E+05 | 3.14E+04 | -0.38 | 2.26E-02 |
| LysoPE(20:1(11Z)/0:0) | 2.96E+06 | 1.82E+05 | 2.38E+06 | 1.89E+05 | -0.32 | 4.29E-02 |
| Normetanephrine | 8.02E+03 | 5.12E+02 | 6.46E+03 | 4.92E+02 | -0.31 | 4.43E-02 |
| L-Fucose | 1.00E+05 | 7.60E+03 | 8.11E+04 | 3.89E+03 | -0.31 | 4.18E-02 |
| Pantothenic acid | 2.41E+05 | 8.26E+03 | 1.96E+05 | 1.46E+04 | -0.30 | 2.30E-02 |
| Saringosterol isomer | 4.04E+06 | 1.97E+05 | 3.30E+06 | 2.20E+05 | -0.29 | 2.68E-02 |
| Uridine diphosphate glucuronic acid | 9.93E+04 | 3.54E+03 | 8.13E+04 | 4.74E+03 | -0.29 | 1.02E-02 |

| | | | | | | |
|---|---|---|---|---|---|---|
| (2R,3R,4R)-2-Amino-4-hydroxy-3-methylpentanoic acid | 4.16E+05 | 2.12E+04 | 3.41E+05 | 1.88E+04 | -0.28 | 1.91E-02 |
| LysoPC(20:2(11Z,14Z)/0:0) | 1.56E+05 | 6.07E+03 | 1.29E+05 | 6.25E+03 | -0.27 | 8.87E-03 |
| Uridine diphosphate-N-acetylgalactosamine | 3.22E+06 | 9.54E+04 | 2.84E+06 | 1.29E+05 | -0.18 | 3.80E-02 |
| N-Acetyl-1-aspartylglutamic acid | 2.14E+08 | 6.50E+06 | 1.91E+08 | 6.66E+06 | -0.16 | 2.65E-02 |
| PE(20:4(6E,8Z,11Z,14Z)+=O(5)/P-18:1(9Z)) | 2.34E+06 | 8.05E+04 | 2.68E+06 | 1.09E+05 | 0.20 | 2.72E-02 |
| Acetylcholine | 6.14E+07 | 2.38E+06 | 7.05E+07 | 3.27E+06 | 0.20 | 4.32E-02 |
| PE(41:7) | 3.75E+05 | 2.59E+04 | 4.41E+05 | 1.17E+04 | 0.23 | 3.95E-02 |
| PE(18:0/18:1(9Z)) | 7.36E+05 | 3.38E+04 | 8.67E+05 | 3.07E+04 | 0.24 | 1.17E-02 |
| PE(16:1(9Z)/22:0) | 7.03E+05 | 3.05E+04 | 8.43E+05 | 2.90E+04 | 0.26 | 4.77E-03 |
| 2-Arachidonoyl glycerol | 5.19E+06 | 2.01E+05 | 6.27E+06 | 4.05E+05 | 0.27 | 4.13E-02 |
| PE(20:4(8Z,11Z,14Z,17Z)/22:6(4Z,7Z,10Z,13Z,16Z,19Z)) | 1.06E+05 | 7.78E+03 | 1.29E+05 | 5.71E+03 | 0.28 | 3.23E-02 |
| 2-Methylglutaric acid | 2.71E+04 | 1.10E+03 | 3.31E+04 | 1.65E+03 | 0.29 | 1.20E-02 |
| Butyric acid | 1.95E+04 | 1.11E+03 | 2.38E+04 | 7.97E+02 | 0.29 | 6.87E-03 |
| SM(d18:1/18:0) | 4.22E+06 | 3.48E+05 | 5.28E+06 | 2.16E+05 | 0.33 | 2.05E-02 |
| trans-Aconitic acid | 2.32E+06 | 1.62E+05 | 3.01E+06 | 1.61E+05 | 0.38 | 8.56E-03 |
| PC(22:5(4Z,7Z,10Z,13Z,16Z)/16:0) | 1.11E+06 | 7.14E+04 | 1.46E+06 | 1.08E+05 | 0.40 | 1.91E-02 |
| N-Acetylglutamine | 2.15E+06 | 1.02E+05 | 2.96E+06 | 2.70E+05 | 0.46 | 2.33E-02 |
| PC(16:0/15:0) | 4.78E+04 | 5.67E+03 | 6.62E+04 | 4.85E+03 | 0.47 | 2.64E-02 |
| Guanidinosuccinic acid | 3.22E+03 | 2.19E+02 | 4.60E+03 | 4.17E+02 | 0.51 | 1.66E-02 |
| 3-Hydroxybutyric acid | 1.60E+05 | 8.45E+03 | 2.31E+05 | 2.22E+04 | 0.52 | 1.89E-02 |
| Adenine | 1.25E+08 | 1.49E+07 | 1.87E+08 | 2.24E+07 | 0.59 | 3.99E-02 |
| PE(22:2(13Z,16Z)/20:4(8Z,11Z,14Z,17Z)) | 1.77E+05 | 2.36E+04 | 2.69E+05 | 1.55E+04 | 0.60 | 5.49E-03 |
| Tiglylcarnitine | 1.48E+04 | 1.17E+03 | 2.39E+04 | 3.65E+03 | 0.69 | 4.80E-02 |
| Deoxycytidine | 3.07E+03 | 4.17E+02 | 5.06E+03 | 7.56E+02 | 0.72 | 4.49E-02 |
| Diaminopimelic acid | 1.37E+03 | 1.74E+02 | 2.36E+03 | 2.57E+02 | 0.79 | 8.36E-03 |
| Biotin amide | 5.47E+05 | 3.13E+04 | 1.01E+06 | 8.86E+04 | 0.88 | 1.46E-03 |
| (S,E)-Zearalenone | 1.60E+05 | 9.83E+03 | 3.06E+05 | 3.21E+04 | 0.93 | 3.31E-03 |
| Alanylproline | 4.12E+03 | 5.71E+02 | 7.89E+03 | 1.17E+03 | 0.94 | 1.78E-02 |

| | | | | | | |
|---|---|---|---|---|---|---|
| N,N-Dimethylformamide | 2.31E+06 | 8.78E+04 | 4.60E+06 | 4.89E+05 | 1.00 | 3.07E-03 |
| Enterodiol | 1.44E+06 | 9.31E+04 | 2.92E+06 | 2.99E+05 | 1.02 | 1.93E-03 |
| Melleolide M | 1.92E+05 | 2.50E+04 | 4.11E+05 | 6.67E+04 | 1.09 | 1.62E-02 |
| N-alpha-Acetyl-L-citrulline | 3.70E+05 | 1.74E+04 | 7.91E+05 | 7.20E+04 | 1.10 | 8.66E-04 |
| L-Allothreonine | 8.33E+06 | 4.88E+05 | 1.92E+07 | 2.06E+06 | 1.20 | 1.58E-03 |
| L-Ergothioneine | 1.55E+06 | 4.48E+04 | 3.74E+06 | 5.13E+05 | 1.27 | 5.23E-03 |
| 2-Hydroxybutyric acid | 8.46E+03 | 1.06E+03 | 2.20E+04 | 3.57E+03 | 1.38 | 8.15E-03 |
| N1-Methyl-4-pyridone-3-carboxamide | 9.73E+04 | 1.53E+04 | 2.61E+05 | 2.13E+04 | 1.43 | 4.69E-05 |
| Val asp  isomer | 2.00E+05 | 5.67E+03 | 5.56E+05 | 4.15E+04 | 1.48 | 1.18E-04 |

**Supplemental Table S6**.

| | Veh_SD | | Form_SD | | | |
|---|---|---|---|---|---|---|
| Compound Id | Mean | SEM | Mean | SEM | $Log_2$ FC | *P*-value |
| DG(16:0/18:2(9Z,12Z)/0:0) | 1.70E+04 | 2.56E+03 | 3.75E+03 | 8.34E+02 | -2.18 | 9.79E-04 |
| Docosatrienoic acid | 1.01E+06 | 9.66E+04 | 4.85E+05 | 5.40E+04 | -1.06 | 5.94E-04 |
| Indole-3-methyl acetate | 3.36E+05 | 7.15E+04 | 1.67E+05 | 2.28E+04 | -1.01 | 4.62E-02 |
| 5-Nonadecyl-1,3-benzenediol | 2.56E+05 | 4.86E+04 | 1.38E+05 | 1.80E+04 | -0.89 | 4.28E-02 |
| Nervonic acid | 8.40E+05 | 1.20E+05 | 4.72E+05 | 5.96E+04 | -0.83 | 1.82E-02 |
| Docosadienoate (22:2n6) | 6.16E+05 | 1.06E+05 | 3.48E+05 | 4.68E+04 | -0.82 | 3.93E-02 |
| Erucic acid | 2.88E+06 | 4.13E+05 | 1.69E+06 | 2.53E+05 | -0.77 | 2.68E-02 |
| Arachidic acid | 1.39E+06 | 2.03E+05 | 8.19E+05 | 1.13E+05 | -0.76 | 2.73E-02 |
| 10Z-Pentadecenoic acid | 1.41E+05 | 2.00E+04 | 9.23E+04 | 9.83E+03 | -0.61 | 4.77E-02 |
| Tetracosahexaenoic acid | 1.66E+06 | 2.17E+05 | 1.10E+06 | 8.29E+04 | -0.6 | 3.28E-02 |
| PG(18:0/16:0) | 4.60E+04 | 5.31E+03 | 3.33E+04 | 1.46E+03 | -0.47 | 4.22E-02 |
| Taurine | 8.47E+07 | 5.72E+06 | 1.07E+08 | 3.13E+06 | 0.34 | 4.15E-03 |
| Butyric acid | 4.34E+04 | 4.60E+03 | 5.61E+04 | 3.83E+03 | 0.37 | 4.79E-02 |
| PC(18:0/18:3(9Z,12Z,15Z)) | 9.29E+05 | 9.06E+04 | 1.25E+06 | 1.13E+05 | 0.43 | 3.77E-02 |
| Pseudouridine | 3.61E+06 | 3.52E+05 | 4.90E+06 | 4.28E+05 | 0.44 | 3.16E-02 |
| Glutarylcarnitine | 2.53E+04 | 2.08E+03 | 3.46E+04 | 3.52E+03 | 0.45 | 3.93E-02 |
| Capryloylglycine | 1.89E+05 | 2.31E+04 | 2.74E+05 | 3.18E+04 | 0.54 | 4.50E-02 |
| 3-methylglutarylcarnitine | 5.94E+04 | 5.87E+03 | 9.89E+04 | 1.25E+04 | 0.73 | 1.35E-02 |
| Aldosterone | 4.87E+05 | 8.77E+04 | 8.71E+05 | 8.22E+04 | 0.84 | 8.42E-03 |
| SM(d18:1/23:0) | 3.78E+04 | 6.33E+03 | 7.48E+04 | 1.22E+04 | 0.98 | 1.78E-02 |
| Corticosterone | 2.81E+04 | 5.11E+03 | 6.68E+04 | 1.39E+04 | 1.25 | 2.39E-02 |
| Indole-3-carboxilic acid-sulphate isomer | 7.13E+05 | 1.08E+05 | 1.77E+06 | 2.47E+05 | 1.31 | 2.01E-03 |
| PC(18:1(9Z)/16:0) | 7.02E+05 | 2.85E+05 | 2.36E+06 | 6.46E+05 | 1.75 | 3.59E-02 |

bioRxiv preprint doi: https://doi.org/10.1101/2025.01.30.635746; this version posted February 1, 2025. The copyright holder for this preprint (which was not certified by peer review) is the author/funder, who has granted bioRxiv a license to display the preprint in perpetuity. It is made available under aCC-BY 4.0 International license.

**Supplemental Table S7.**

| Dependent Variable | Source | Type III Sum of Squares | df | Mean Square | F | Sig. | Partial Eta Squared |
|---|---|---|---|---|---|---|---|
| Thermal (Ipsilateral) | Corrected Model | 554.178 | 6 | 92.363 | 29.654 | <.001 | 0.813 |
| | Intercept | 2358.363 | 1 | 2358.363 | 757.175 | <.001 | 0.949 |
| | BW | 10.658 | 1 | 10.658 | 3.422 | 0.072 | 0.077 |
| | MD-1 | 481.944 | 2 | 240.972 | 77.366 | <.001 | 0.791 |
| | PFD | 22.214 | 1 | 22.214 | 7.132 | 0.011 | 0.148 |
| | MD-1 * PFD | 17.788 | 2 | 8.894 | 2.856 | 0.069 | 0.122 |
| | Error | 127.702 | 41 | 3.115 | | | |
| | Total | 3129.472 | 48 | | | | |
| | Corrected Total | 681.88 | 47 | | | | |
| Thermal (Contralateral) | Corrected Model | 410.877 | 6 | 68.479 | 13.637 | <.001 | 0.666 |
| | Intercept | 3381.238 | 1 | 3381.238 | 673.337 | <.001 | 0.943 |
| | BW | 5.594 | 1 | 5.594 | 1.114 | 0.297 | 0.026 |
| | MD-1 | 336.72 | 2 | 168.36 | 33.527 | <.001 | 0.621 |
| | PFD | 0.068 | 1 | 0.068 | 0.014 | 0.908 | 0 |
| | MD-1 * PFD | 0.919 | 2 | 0.459 | 0.091 | 0.913 | 0.004 |
| | Error | 205.886 | 41 | 5.022 | | | |
| | Total | 4024.143 | 48 | | | | |
| | Corrected Total | 616.763 | 47 | | | | |
| Mechanical (Ipsilateral) | Corrected Model | 96.994 | 6 | 16.166 | 65.436 | <.001 | 0.905 |
| | Intercept | 367.227 | 1 | 367.227 | 1486.476 | <.001 | 0.973 |
| | BW | 0.027 | 1 | 0.027 | 0.109 | 0.744 | 0.003 |
| | MD-1 | 69.051 | 2 | 34.526 | 139.754 | <.001 | 0.872 |
| | PFD | 8.695 | 1 | 8.695 | 35.198 | <.001 | 0.462 |
| | MD-1 * PFD | 8.428 | 2 | 4.214 | 17.058 | <.001 | 0.454 |
| | Error | 10.129 | 41 | 0.247 | | | |
| | Total | 482.211 | 48 | | | | |
| | Corrected Total | 107.122 | 47 | | | | |
| Mechanical (Contralateral) | Corrected Model | 67.359 | 6 | 11.227 | 15.798 | <.001 | 0.698 |
| | Intercept | 517.257 | 1 | 517.257 | 727.874 | <.001 | 0.947 |
| | BW | 1.272 | 1 | 1.272 | 1.79 | 0.188 | 0.042 |
| | MD-1 | 65.028 | 2 | 32.514 | 45.753 | <.001 | 0.691 |
| | PFD | 0.562 | 1 | 0.562 | 0.791 | 0.379 | 0.019 |
| | MD-1 * PFD | 0.261 | 2 | 0.13 | 0.184 | 0.833 | 0.009 |
| | Error | 29.136 | 41 | 0.711 | | | |
| | Total | 630.762 | 48 | | | | |
| | Corrected Total | 96.495 | 47 | | | | |

bioRxiv preprint doi: https://doi.org/10.1101/2025.01.30.635746; this version posted February 1, 2025. The copyright holder for this preprint (which was not certified by peer review) is the author/funder, who has granted bioRxiv a license to display the preprint in perpetuity. It is made available under aCC-BY 4.0 International license.

**Supplemental Table S8.**

| | MD-1 | MD-2 | MD-3 | MD-4 | MD-5 |
|---|---|---|---|---|---|
| Phase I | — | — | — | — | — |
| Phase II | ↓↓ | — | — | ↓↓ | — |
| Edema | ↓↓ | — | — | — | ↓↓ |
| IPSI T | ↓↓↓ | ↓↓↓ | — | — | — |
| M | ↓↓↓ | ↓↓ | — | — | — |
| CON T | ↓↓↓ | ↓↓↓ | — | — | — |
| M | ↓↓↓ | ↓↓↓ | — | — | — |
| EPM | ↓↓↓ | ND | ND | — | ↓↓ |
| NOR | ↓↓↓ | ND | ND | ↓↓↓ | — |
